# Generation of a Predictive Melphalan Resistance Index by Drug Screen of B-Cell Cancer Cell Lines

**DOI:** 10.1371/journal.pone.0019322

**Published:** 2011-04-29

**Authors:** Martin Boegsted, Johanne M. Holst, Kirsten Fogd, Steffen Falgreen, Suzette Sørensen, Alexander Schmitz, Anne Bukh, Hans E. Johnsen, Mette Nyegaard, Karen Dybkaer

**Affiliations:** Department of Haematology, Aalborg Hospital Science and Innovation Center, Aarhus University Hospital, Aalborg, Denmark; Cleveland Clinic, United States of America

## Abstract

**Background:**

Recent reports indicate that *in vitro* drug screens combined with gene expression profiles (GEP) of cancer cell lines may generate informative signatures predicting the clinical outcome of chemotherapy. In multiple myeloma (MM) a range of new drugs have been introduced and now challenge conventional therapy including high dose melphalan. Consequently, the generation of predictive signatures for response to melphalan may have a clinical impact. The hypothesis is that melphalan screens and GEPs of B-cell cancer cell lines combined with multivariate statistics may provide predictive clinical information.

**Materials and Methods:**

Microarray based GEPs and a melphalan growth inhibition screen of 59 cancer cell lines were downloaded from the National Cancer Institute database. Equivalent data were generated for 18 B-cell cancer cell lines. Linear discriminant analyses (LDA), sparse partial least squares (SPLS) and pairwise comparisons of cell line data were used to build resistance signatures from both cell line panels. A melphalan resistance index was defined and estimated for each MM patient in a publicly available clinical data set and evaluated retrospectively by Cox proportional hazards and Kaplan-Meier survival analysis.

**Principal Findings:**

Both cell line panels performed well with respect to internal validation of the SPLS approach but only the B-cell panel was able to predict a significantly higher risk of relapse and death with increasing resistance index in the clinical data sets. The most sensitive and resistant cell lines, MOLP-2 and RPMI-8226 LR5, respectively, had high leverage, which suggests their differentially expressed genes to possess important predictive value.

**Conclusion:**

The present study presents a melphalan resistance index generated by analysis of a B-cell panel of cancer cell lines. However, the resistance index needs to be functionally validated and correlated to known MM biomarkers in independent data sets in order to better understand the mechanism underlying the preparedness to melphalan resistance.

## Introduction

The alkylating agent, melphalan, is the backbone of current therapy in MM. Since the 1990s, melphalan has been used in high dose therapy (HDT) followed by autologous stem cell transplantation (ASCT) [1] and has as such improved the response rate, as well as prolonged event free survival (EFS) and overall survival (OS) [2]. Even though the last years have seen considerable improvements, the overall survival remains dismal and the disease is considered incurable – mainly due to an initial refractory disease or induced resistance resulting in disease relapse. Refractory disease and early relapse is considered associated with the development of melphalan resistance which is a complex phenomenon not completely understood [3]. One possible strategy for improving the knowledge about drug resistance is the combined use of novel technologies including GEP and drug screen in a preclinical malignant B-cell cancer cell line model [4].

The fundamental idea of recent studies on drug resistance has been to categorize cell lines into sensitive, resistant and intermediate groups based on drug dose response experiments and subsequently to generate a genetic classifier or signature based on microarray analysis. Publicly available data from the NCI60 cell line panel generated by the National Cancer Institute (NCI) have been used extensively in such studies for various cancer types and treatment regimes. However, the approach remains controversial [5], [6]. Several authors have argued that the performance could be improved by a specific cell line panel. Such an approach was used by Lee et al. [7] and Liedtke et al. [8] for bladder and breast cancer tumors, respectively. The successful approach of Lee et al. [7] was based on the selection of gene expressions for the organ specific cell lines which correlate with gene expressions in patient material before developing their classifier by a misclassification-penalized posterior algorithm. However, Liedtke et al. [8] were unable to predict the outcome of chemotherapy response with an approach based on diagonal linear discriminant analysis (DLDA) for classification.

The concept of the present study is that melphalan resistance in MM can be studied in a preclinical model of malignant B-cell cancer cell lines by combining drug screens and GEPs and generate a gene signature for resistance, which clinically can be validated by predicting the outcome for tumors analysed before high dose melphalan and ASCT. Such a strategy involves intensive data generation in the laboratory and is succeeded by use of data management and advanced statistical analysis [5], [6]. In the present study, we have implemented reproducibility by scripting the entire data analysis flow in R and Bioconductor.

In summary, the specific aims of this study were to develop a melphalan resistance gene index by use of 1) the publicly available cell line panel NCI60 or 2) a panel of B-cell cancer cell lines and 3) to support the concept though available “on-line” microarrays and clinical data set from MM patients treated with double high dose melphalan [9].

## Materials and Methods

### The NCI60 Cell Line Panel

The NCI60 cell line screen method is developed by NCI and serves to screen a large number of substances for cytotoxic activity. The panel consists of 59 cell lines derived from distinct cancer types [10], [11]. The gene expression data and chemotherapy sensitivity data are publicly available. For more information, see the Online Information Section below. In the present study we used the GI_50_ value as defined by NCI [12].

### B-Cell Cancer Cell Lines and Culturing Conditions

The BCell panel consisted of 13 MM cell lines, 1 plasmacytoma (PC) cell line and 4 diffuse large B-cell lymphoma (DLBCL) cell lines. The cell lines were cultured under standard conditions at 37°C; in a humidified atmosphere of 95% air/5% CO_2_ with the appropriate medium, fetal bovine serum (FBS) and 1% penicillin/streptomycin addition. See Table S1. The cell lines were maintained for a maximum of 20 passages to minimize any long-term culturing effects. Penicillin/streptomycin 1%, RPMI1640, IMDM and FBS were purchased from Invitrogen. The cell lines KMM-1 and KMS-11 were obtained from JCRB (Japanese Collection of Research Bioresources), and KMS-12-PE, KMS-12-BM, LP-1, MM1S, MOLP-2, MOLP-8, NCI-H929, OPM-2, RPMI-8226, U-266, AMO-1, DB, HT and SU-DHL-4 from DSMZ (Deutsche Sammlung von Mikroorganismen und Zellkulturen). The cell line MM1S was provided by Steven T. Rosen [13], RPMI-8226 LR5 by William S. Dalton [14] and OCI-Ly7 by Hans Messner [15].

### Melphalan Dose Response Experiments

The cell number in the culture was determined by absorbance measurements (CellTiter 96 Aqueous One Solution Reagent, Promega) as described by the manufacturer. The linear relationship between absorbance and cell number was obtained by seeding cells in 96-well plates with the appropriate medium at concentrations ranging between 15–60000 cells/well. The 18 cell lines were incubated for 24 hours before the addition of 18 increasing concentrations of melphalan in triplicates. All wells were seeded with cells but border effects were circumvented by including only non-border wells for analysis. The melphalan was resolved in ethanol resulting in a final ethanol concentration of 0.06% in the medium. The relative cell number was measured 48 hours after the addition of melphalan using the CellTiter reagent and the Optima-Fluostar (BMG LABTECH) at 492 nm. To achieve high reproducibility, the whole experiment was repeated at least twice utilizing new freeze stocks of the individual cell lines.

### RNA Microarray Analysis

All GEPs were performed using the Affymetrix microarray platform and standard procedures. Total RNA was extracted using Invitrogen TRIzol Reagent combined with Qiagen RNeasy Mini kit. The quality was checked by Agilent 2100 Bioanalyzer. The samples were prepared for hybridization to Affymetrix GeneChip HG-U133 Plus 2.0 arrays after the manufacturer's instruction and .CEL-files were generated by Affymetrix GeneChip Command Console Software (AGCC) and deposited at the NCBI Gene Expression Omnibus (GEO) repository. The data fulfil the requirements of being MIAME compliant. For more information, see the Online Information Section.

### Arkansas and Hummel Cohorts of MM and DLBCL Patients

Gene expression data, EFS, and OS data for 565 patients diagnosed with progressive or symptomatic MM are publicly available. For more information, see the Online Information Section. The data set is known as the “Arkansas data” [16]. The patients were enrolled by The Myeloma Institute for Research and Therapy, University of Arkansas, School of Medical Sciences, and they were part of a larger study with the purpose to investigate whether thalidomide in combination with HDT can prolong survival among patients with MM [9]. The 565 patients were treated according to the total therapy two (TT2) or total therapy three (TT3) protocol including double high dose melphalan and ASCT.

The data set known as the “Hummel data” [17] was used in the present study to test the specificity of the identified resistance index. The 87 patients were diagnosed with DLBCL and received a CHOP-like (cyclophosphamide, doxorubicin, vincristine, and prednisone) induction treatment. Gene expression and OS data are publicly available as well (for more information, see the Online Information Section).

### Statistical Analysis

Full documentation of the statistical analysis is provided by a Sweave document, see Text S1. Sweave is a feature in the statistical programming language R that enables the integration of R code into LaTex and thereby it provides reproducible data analysis and research [18]. All statistical analyses were done with R [19] version 2.12.1 and a number of Bioconductor [20] packages. Detailed session information is contained in Text S1.

#### Melphalan Dose Response Analysis

The absorbance values originating from the dose response experiments were background corrected and averaged over replicates. Eventual outliers among the triplicated cell concentrations were removed by Grubbs' test [21] (approximately 0.5%, see Text S1). Relative growth inhibition curves were calculated for each concentration relative to the untreated control, whereafter a piecewise linear growth curve was modelled. Through visual inspection, five extreme values were removed (Figure S1). The GI_50_ values of the cell lines in the BCell panel were defined as the first point at which the growth curve drops below the 50% level. Data were averaged over the replicated cell line measurements to perform this analysis. The uncertainty of the GI_50_ values was assessed by sub-sampling the replicated wells with replacement and a re-calculation of all the GI_50_ values 200 times. The 10-fold logarithm of the GI_50_ values was transformed to the log_10_ µM-scale for both cell line panels and used as a melphalan resistance index – in the following denoted the NCI60 index and BCell index, respectively. As a means to distinguish between sensitive, intermediate and resistant subjects (cell lines or individuals) in a population, we chose the criterion of Havaleshko et al. [22], where a subject is resistant if its resistance index exceeds the 75 percentile of the population. Similarly, we defined a subject to be sensitive if its resistance index was less than the 25 percentile of the population. The remaining subjects were characterized as having intermediate resistance.

#### Microarray Pre-processing

The BCell .CEL-files and the downloaded NCI60 .CEL-files were background corrected and normalized by the just.rma function from the affy package. All RMA-normalized arrays passed the statistical quality control provided by the function arrayQualityMetrics in the R-package arrayQualityMetrics [23]. As the NCI60 panel was analyzed on the HG-U133a array and BCell on the HG-U133 Plus 2.0 array, focus was on probes only present on the HG-U133A array. The Arkansas data were also background corrected and normalized with just.rma.

#### Differential Expressions

Following the unspecific filtering of the gene expression data, the cell lines were ranked as resistant, intermediate or sensitive according to their GI_50_ values. Transcripts that expressed significant differences between the groups of the most sensitive and most resistant cell lines were determined using moderated F-tests as implemented in the Bioconductor package limma [24]. Genes with a P-value below 0.05 were considered to have predictive value. The P-values were deliberately chosen instead of false discovery rates as the purpose was to construct a resistance classifier and not to detect differentially expressed genes. The differentially expressed genes were scaled to have zero mean and standard deviation one. A classifier was built by the scaled genes and linear discriminant analysis (LDA) as implemented in the R-package sda [25]. To avoid difficulties inverting large covariance matrices, a maximum of 400 genes in sda was chosen.

#### Multivariate Regression

The genes were filtered according to sure independence screening (SIS), i.e. all genes were ranked according to the Pearson correlation coefficient between its gene expression and resistance index. All genes, for which the P-value of the test for zero correlation was above 0.05, were considered for dimensionality reduction by SPLS [26]. To obtain sparsity, SPLS penalizes the transformed input vectors by forcing small coefficients to be zero. The pure SPLS formulation contains four tuning parameters, however, according to Chun et al. [26], a simple SPLS regression formulation, which only depends on one parameter *η*, is controlling the sparsity of the solution and the number of hidden components *K*. For particular choices of the regularization parameter *η* and the hidden components *K* the performance was evaluated by leave-one-out cross-validations. The optimal configuration of the parameters was chosen to be the set minimizing the mean squared prediction error (MSPE). Once the optimal parameters have been chosen internally from the cell lines, the resistance index can be predicted for the subjects through a linear combination of the scaled gene expressions with the coefficients estimated by SPLS [27]. The SPLS analysis and predictions are performed with the R-package spls provided by Chun et al. [26].

#### Independent Filtering

It is well-known that independent filtering increases detection power for high-throughput experiments [28]. To investigate whether independent filtering would increase accuracy and prediction error, an unspecific filtering, leaving out genes with low variation over the NCI60 and BCell gene expressions, were carried out with the function nsFilter from the Bioconductor package genefilter. The cut-off values varied between 0% and 100% and we chose the cut-off value which performed best with respect to cross-validated accuracy for the LDA and MSPE for SPLS. In order to investigate whether any predictive power remained after filtering, cross-validation was performed for the chosen parameters.

#### Survival Analysis

Kaplan-Meier survival analysis, logrank test and Cox proportional hazards models were calculated with functions from the R-package survfit. A nonlinear relationship between the predicted response to treatment and the resistance index was noticed and the relationship was estimated by restricted cubic splines (RCS) by means of the R-package Design [29]. The significance level is set to 0.05 and the hazard ratios (HR) are given with 95% confidence intervals.

### Online Information

Details on the required and deposited on-line information are described below.

#### The BCell Gene Expression Data

.CEL files for the 18 cell line microarrays have been deposited at http://www.ncbi.nlm.nih.gov/geo/ under GEO accession number GSE22759. The data fulfil the requirements to be MIAME compliant.

#### The NCI60 Gene Expression Data

.CEL-files for the NCI60 cell line microarrays were downloaded from http://www.ncbi.nlm.nih.gov/geo/ under GEO accession number GSE5720 by selecting the subset of data originating from the HG-U133A array. The cell line IGROV1 is provided in dublicates – in the present study replicate A21 is used. The data fulfil the requirements to be MIAME compliant. Notice that we have renormalized the .CEL files as described in the Materials and Methods Section.

#### The NCI60 DTP Data

The DTP human tumor cell line screening data (August 2008 release) were obtained by downloading the file cancer60gi50.lis from the website: http://dtp.nci.nih.gov/docs/cancer/cancer_data.html. Parts of the script for extracting NCI60 drug response have been developed by Kevin Coombes and Keith Baggerly and can be downloaded from the website http://bioinformatics.mdanderson.org/Supplements/ReproRsch-Chemo/.

#### The Arkansas Gene Expression and Clinical Data

.CEL files for the gene expression data and clinical information are available at http://www.ncbi.nlm.nih.gov/geo/ under GEO accession number GSE24080. The data fulfil the requirements to be MIAME compliant. The .CEL files are renormalized as described in the Materials and Methods Section.

#### The Hummel Gene Expression and Clinical Data

.CEL files and clinical information are available at http://www.ncbi.nlm.nih.gov/geo/ under GEO accession number GSE4475. The data fulfil the requirements to be MIAME compliant. The .CEL files are renormalized as described in the Materials and Methods Section.

## Results

### The NCI60 Panel Resistance Index

In brief summary, dose response data for melphalan were downloaded. A plot of the data is seen in Figure S2. The 59 cell lines showed GI_50_ values ranging from −5.77 to −3.99 on the log_10_ µM/ml scale - the most sensitive cell line being SR and the most resistant cell line being A498.

### Developing the B-Cell Resistance Index

**Figure 1:**
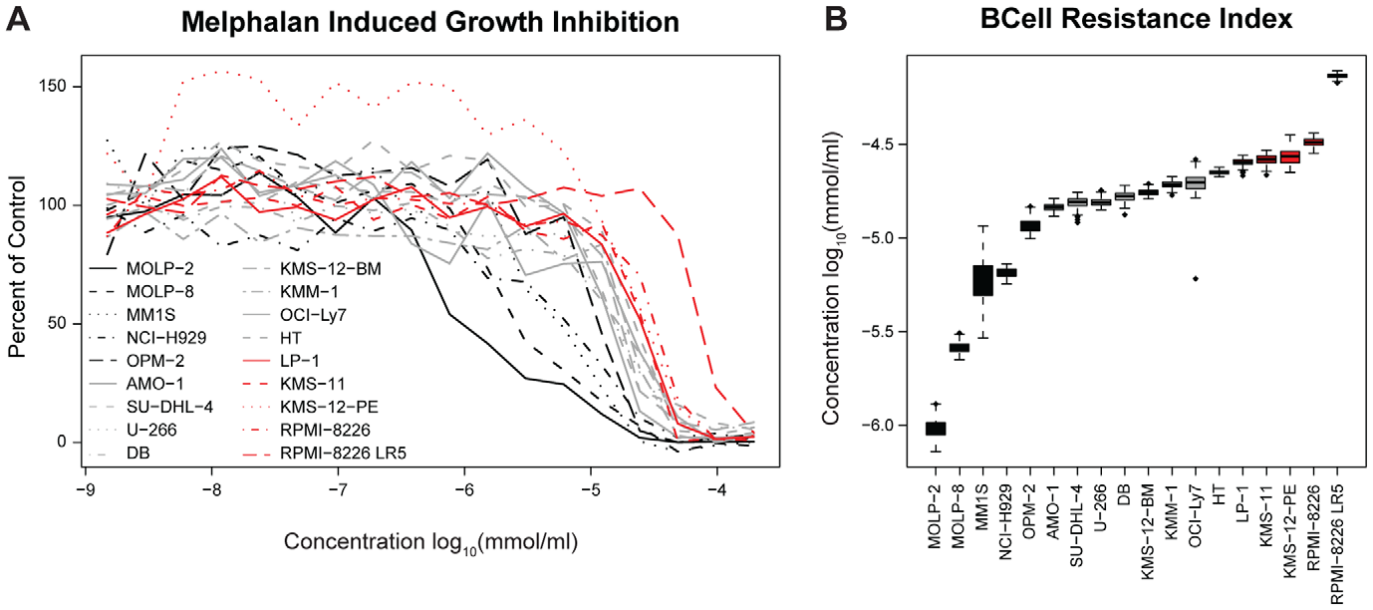
Melphalan dose-response summary. A) Averaged dose-response curves for each cell line. B) Box plot of 200 resampled GI_50_ values for each cell line. The cell lines are ranked according to their estimated GI_50_ value.

Dose response experiments were carried out, and plots of the data as well as fitted curves are illustrated in Figure 1A. The 18 cell lines showed GI_50_ values ranging from −6.02 to −4.13 on the log_10_ µM/ml scale - the most sensitive cell line being MOLP-2 and the most resistant cell line being RPMI-8226 LR5. Figure 1B shows box plots of the mean GI_50_ value from re-sampled dose-response curves for all 18 B-cell cancer cell lines. As no clear distinction between a resistant and sensitive group of cell lines was detected, the 25%/50%/25% split described in the Materials and Methods Section was chosen, i.e. the five cell lines with the lowest GI_50_ values were denoted sensitive and the five cell lines with the highest GI_50_ values were denoted resistant.

### Classifier Based on LDA

For the NCI60 panel, an LDA based classifier was built as outlined in the Materials and Methods section, for details see Text S1. The LDA based classifier showed poor internal validation (Figure S3). The optimal accuracy (determined by leave-one-out cross-validation) of 0.6 was obtained for the BCell panel at a filtering rate of 0.95, in which case the moderated F-test gave 159 genes (Table S2). LDA was used to combine the 159 genes to develop a classifier. The classifier showed 60% overall leave-one-out-cross-validation accuracy for the cell lines from which it was developed.

### Cross-Validating the SPLS Model

After the unspecific filtering steps were attained, SPLS was used to achieve specific filtering. In order to avoid over-fitting and noise contributing genes, the number of hidden components and probesets were chosen by leave-one-out cross-validation. The optimal number of probesets and components were found at the values where the minimal MSPE was attained. For the NCI60 panel, a reasonable internal validation was observed (Figure S4). For the BCell panel, two hidden components and 19 probesets provided the best MSPE (Figure S5). The leverage of a single cell line on the prediction model was investigated by plotting the predicted resistance value originating from the leave-one-out-cross-validation versus the measured resistance index (Figure S6). The most sensitive and resistant cell lines MOLP-2 and RPMI-8226 LR5, respectively, turned out to be high leverage points.

### Stability Evaluation

To see how SPLS regression copes with noise, the BCell panel was used to select 20 probesets randomly among the 100 probesets with the highest marginal association (absolute value of the Pearson correlation coefficient) with the resistance index. In order to keep the dependence structure between the probesets intact, these were all randomly perturbed, except for the 20 probesets. The coefficients of the probesets are shown as a function of the regularization parameter *η* in Figure S7. For this example, the optimal number of sparse partial least squares components was *K = *3 and the optimal regularization parameter was *η = *0.83. Eleven probesets were chosen, which demonstrates an average sensitivity of 55%, a specificity of 99% and a false discovery rate of 63%. The experiment was repeated 100 times and gave in average a sensitivity of 54%, a specificity of 99% and a false discovery rate of 67%.

### Comparison of the most Sensitive and Resistant Cell Lines

Due to the high influence of the most sensitive cell line, MOLP-2, and the most resistant cell line, RPMI-8226 LR5, a direct comparison of these two cell lines was made. This was done by sorting the genes according to their absolute difference in gene expression and choosing (quite arbitrarily) the 100 genes with the highest absolute differential expressions. A predictive resistance index was constructed by taking the difference in gene expressions as weight. The genes and their weights are shown in the supporting information (Table S3).

### External Validation on Clinical Samples

EFS and OS were chosen as end points with the hypothesis that melphalan resistance is correlated to these end points. For the NCI60 and BCell panels, the LDA and SPLS models as well as the model consisting of the two influential cell lines in the BCell panel were used to estimate the melphalan resistance index for each of the Arkansas patients.

**Figure 2:**
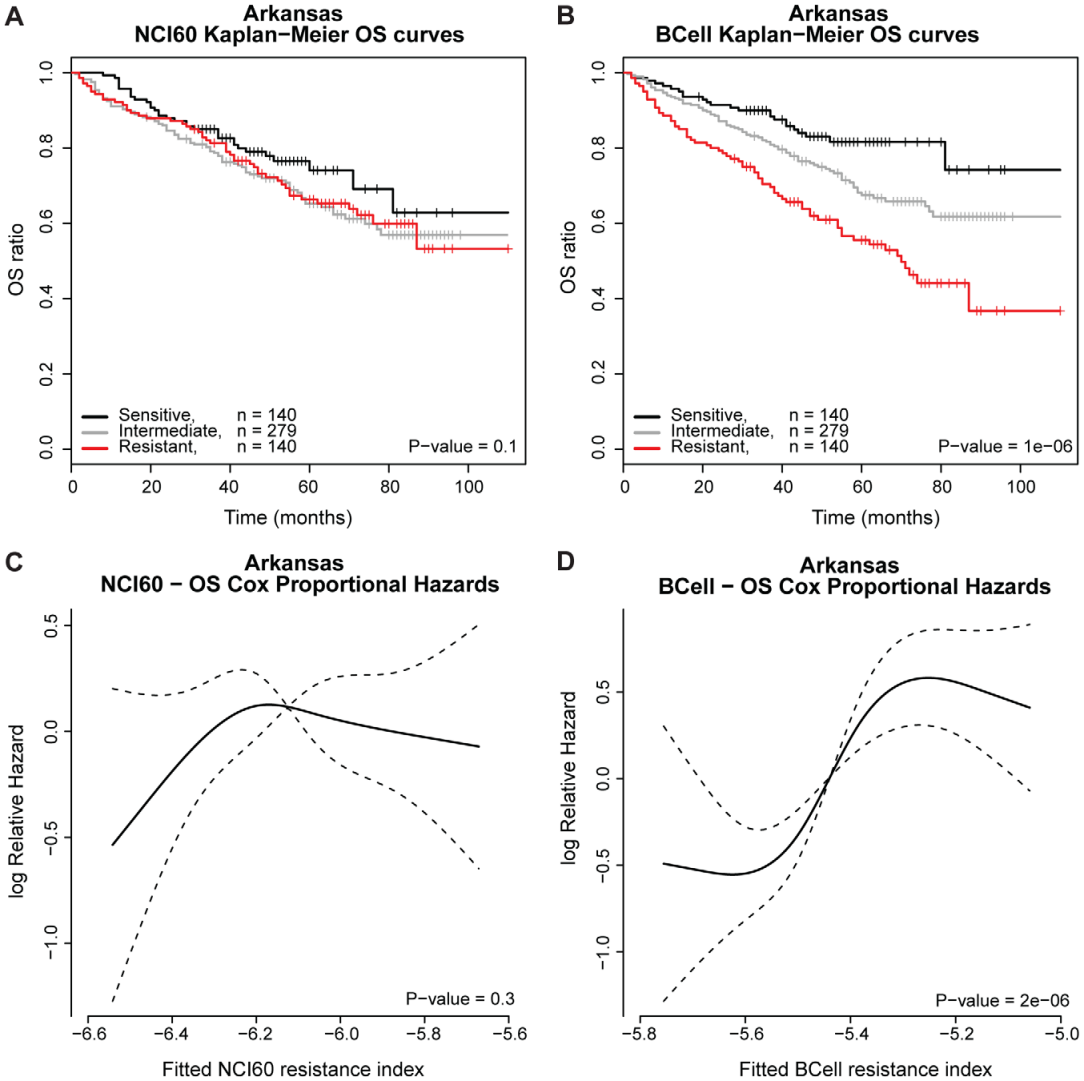
OS analysis for the Arkansas data. A) Kaplan Meier survival curves based on NCI60. B) Kaplan Meier survival curves based on BCell. The samples are categorized into a 25% most sensitive risk group, an intermediate risk group of 50% and a 25% high risk group, based on the melphalan resistance index. The P-value is the logrank test for no difference in survival curves. C) Log relative hazard as function of the NCI60 resistance index. D) Log relative hazard as a function of the BCell resistance index. The P-value is the maximum likelihood test for no RCS-association between log relative hazard and resistance index and the dashed lines represent 95% confidence intervals.

**Figure 3:**
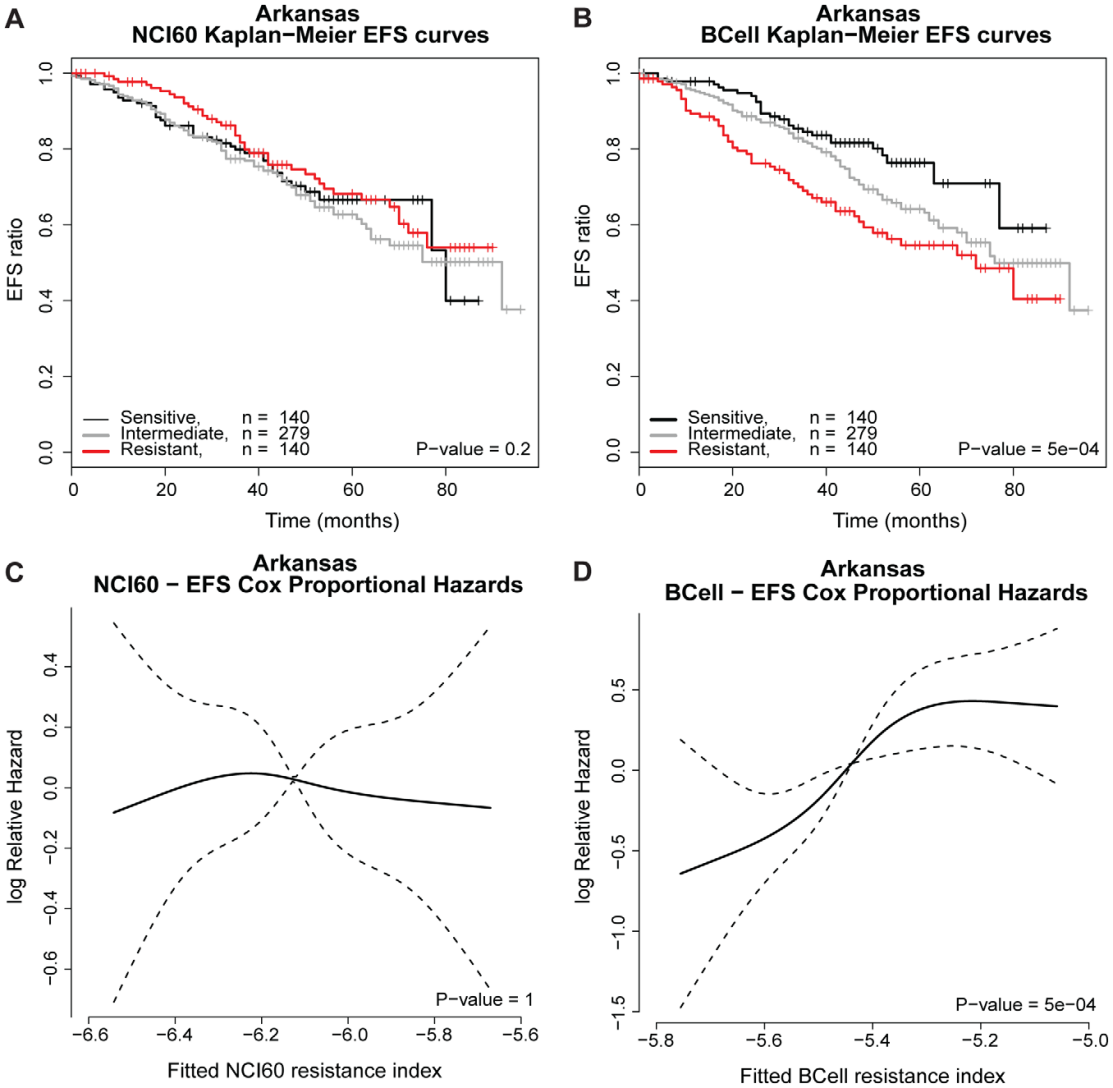
EFS analysis for the Arkansas data. A) Kaplan Meier survival curves based on NCI60. B) Kaplan Meier survival curves based on BCell. The samples are categorized into a 25% most sensitive risk group, an intermediate risk group of 50% and a 25% high risk group, based on the melphalan resistance index. The P-value is the logrank test for no difference in survival curves. C) Log relative hazard as function of the NCI60 resistance index. D) Log relative hazard as a function of the BCell resistance index. The P-value is the maximum likelihood test for no RCS-association between log relative hazard and resistance index and the dashed lines represent 95% confidence intervals.

For the LDA based predictions based on the NCI60 panel no significant difference was observed with respect to OS and EFS for the predicted sensitive, intermediate and resistant groups of patients (Figure S8 and S9). For the NCI60 and SPLS based predictions no significant difference was found for the predicted sensitive, intermediate and resistant groups as well as the predicted log relative hazard of OS and EFS for the Arkansas patient data. See Figure 2A and C and Figure 3A and C, respectively.

For the BCell panel based SPLS model, the Kaplan-Meier survival analysis is shown in Figure 2B and Figure 3B to illustrate the distinction between the predicted resistant, intermediate and sensitive groups for the Arkansas data. We detected a significant difference in OS (P-value<0.001) and EFS (P-value<0.001) for the three groups of patients. A Cox proportional hazards model was used to detect that patients predicted melphalan sensitive have significantly superior survival (HR = 2.9 [2.41: 3.35]) and longer time to relapse (HR = 2.2 [1.75: 2.67]) compared to resistant patients for the BCell panel. The log relative hazards versus a RCS-model for the resistance index are depicted in Figure 2D and Figure 3D for the Arkansas OS and EFS data, respectively. There is a significant tendency of shorter time to death (P-value<0.001) and relapse (P-value<0.001) with increasing resistance index for the BCell panel.

The LDA-classifier was used to predict whether the patients in the Arkansas cohort of patients were sensitive or resistant towards melphalan. A significant difference for both the OS (P-value = 0.006) and EFS (P-value<.001), with respect to the BCell panel derived LDA classifier, was detected (Figure S10 and S11).

A significant difference in OS (P-value = 0.004) and EFS (P-value<0.001) between the patients categorized with respect to the two influential cell lines were also shown (Figures S12 and S13).

### Potential Marker Transcripts

SPLS with the optimal choices *η = *0.82 and *K = *2 identified 19 probesets with non-zero coefficients. Probesets, gene symbols and names, biological function as well as chromosome locations and regression weights are listed in Table 1.

**Table 1:** The generated probesets predicting melphalan resistance.

U133 ID	Gene Symbol	Name	Location	Weight
205990_s_at	*WNT5A*	Wingless-type MMTV integration site family, member 5A	3p21-p14	−0.065
203708_at	*PDE4B*	Phosphodiesterase 4B, cAMP-specific	1p31	−0.053
201990_a_at	*CREBL2*	cAMP responsive element binding protein-like 2	12p13	−0.046
218751_s_at	*FBXW7*	F-box and WD repeat domain containing 7	4q31.3	−0.044
201889_at	*FAM3C*	family with sequence similarity 3, member C	7q31	−0.039
206405_x_at	*USP6*	USP6 N-terminal like	17p13	−0.038
219049_at	*CSGALNACT1*	Chondroitin sulfate N- acetylgalactosaminyltransferase 1	8p21.3	−0.037
205862_at	*CREB1*	cAMP responsive element binding protein 1	2p25.1	−0.034
219748_at	*TREML2*	Triggering receptor expressed on myeloid cells-like 2	6p21.1	−0.033
204786_s_at	*IFNAR2*	Interferon (alpha, beta and omega) receptor 2	21q22.1,21q22.11	−0.033
204204_at	*SLC31A2*	Solute carrier family 31 (copper transporters), member 2	9q31-q32	−0.025
217825_s_at	*UBE2J1*	Ubiquitin-conjugating enzyme E2, J1 (UBC6 homolog, yeast)	6q15	−0.020
213555_at	*RWDD2A*	RWD domain-containing protein 2A-like	6q14.2	−0.019
212122_at	*RHOQ*	Ras homolog gene family, member Q	2p21	−0.016
203895_at	*PLCB4*	Phospholipase C, beta 4	20p12	−0.015
202043_s_at	*SMS*	Spermine synthase	Xp22.1	0.011
217104_at	*ST20*	Suppressor of tumorigenicity 20	15q25.1	0.012
212055_at	*C18orf10*	Chromosome 18 open reading frame 10	18q12.2	0.025
221210_s_at	*NPL*	N-acetylneuraminate pyruvate lyase	1q25	0.032

### Melphalan Resistance Index in DLBCL

For the BCell panel, no significant association between the SPLS based resistance index and OS was found for the Hummel data set (Figure S14 and S15).

## Discussion

Motivated by the clinical importance of melphalan therapy, we have combined *in vitro* drug screens and microarray data of B-cell cancer cell lines and identified a melphalan resistance index comprised of 19 genes which may be related to tumor biology. In order to validate the resistance index it was tested in a publicly available retrospective data set consisting of GEP data from the myeloma tumor of MM patients receiving treatment including high dose melphalan and a DLBCL trial, where patients never received melphalan treatment.

Several reports have used publicly available GEPs and *in vitro* drug response information from the NCI to develop drug-specific pharmacogenomics response predictors. However, the idea of cell line derived predictors is controversial and has been criticized. Despite methodologically and conceptually difficult factors involved in this strategy, it has not discouraged us to explore similar avenues for molecular predictor discovery in MM and multivariate bioinformatics tools. During the implementation of the present strategy, we have identified several of such factors related to the drug screen assay, the statistical approach, the function of the identified genes, and most importantly, the clinical validation as discussed below.

Firstly, the use of other toxicity measures than GI_50_ might as well be relevant and reflect other biological mechanisms. Moreover, the drug screen assay depends on the inhibition of cell proliferation which is a central player in the efficacy of the selected drug. However, other biological functions like apoptosis, cell differentiation and DNA repair may also be involved in the drug effects.

Secondly, in high dimensional classification and regression techniques it is unavoidable that some of the genes contribute with noise to the clinical predictions. The noise was expected to be minimized using a sparse version of PLS where the number of hidden components and transcripts were selected by leave-one-out cross-validation. A reasonable sensitivity and specificity were attained by stability evaluation. However, also a high false discovery rate was achieved, but one should notice that the simulation example was designed for marginal association and not for optimal performance with respect to SPLS. An important by-product of the multivariate statistical analysis was the emphasizing of influential observations.

As described below, further elimination of false positive genes may be pursued by gene enrichments and functional studies. It is important to note that during the development of the resistance index signature, we made several selections with regards to the employed statistical methods and other decisions may have resulted in similar or better results for the cell lines in general and NCI60 in particular.

Thirdly, melphalan is an alkylating agent that introduces inter-strand cross-links in DNA, and it could therefore be expected that some of the genes involved in the melphalan resistance index would be linked to DNA damage response by the Fanconi anaemia (FA)/BRCA pathway as described by e.g. Yarde et al. [3] and Chen et al. [30], or by other DNA damage repair pathways. The resistance index, however, was based upon gene expression levels prior to drug treatment, and a drug-induced activation of the DNA repair response would therefore not be detected. In general, the genes in Table 1 encode a functionally diverse group of genes coding for proteins which are involved in numerous key pathways. This indicates that several factors are involved in determining the level of preparedness of a malignant cell to resist melphalan. Interestingly, however, three of the genes in Table 1 (*FBXW7*, *USP6*, and *UBE2J1*) are involved in ubiquitin regulated pathways [31]–[33] and DNA damage responses have been shown to be highly dependent on ubiquitin signalling (reviewed by Messick and Greenberg [34]). Kimura et al. [35] have shown that DNA damage can induce *FBXW7* expression via a p53-dependent pathway, wherefore it would be interesting to investigate if *FBXW7* expression is linked to melphalan sensitivity through a DNA damage response pathway. In addition, the function of Wnt-5a is highly dependent upon ubiquitin proteasome pathways [36] and the gene is significant in cancer development and is active during embryogenesis, hematopoietic stem cell growth, cell differentiation and tissue development and has been documented to be of biological relevance in MM [37].

Other of the genes in Table 1 represent interesting candidates for further investigation, including *CSGALNACT1* which at high expression levels previously has been shown to be associated with improved prognosis for MM patients treated with melphalan [38]. *CSGALNACT1* encodes for a protein involved in the synthesis of chondroitin sulphate [39] – a component of Syndecan-1 (CD138) [40] which is known to have a major impact in MM pathogenesis.

Finally, this study introduces a melphalan resistance index predicting EFS and OS of MM patients treated with the double high dose melphalan in the transplantation strategies described as TT2 and TT3. A number of endpoints define the response to melphalan treatment, e.g. immediate response, EFS or OS [41] – each of these reflecting an effect on the biology of the malignant clone. In the study of melphalan it is important to recognize that the remission status is a difficult end point for evaluation as it is also influenced by an effect of the induction therapy and maintenance.

In summary, a gene expression signature capable of predicting response to melphalan therapy in a focused cell line panel has been established by use of SPLS. The utility of the predictor was retrospectively validated on data sets from patients diagnosed with MM and treated with high dose melphalan as well as on a control study of patients with DLBCL never treated with melphalan. The lack of association between predicted melphalan resistance and OS in the DLBCL study suggests that the resistance index is melphalan specific and our future studies will address this for MM patients in specific European clinical trial data sets.

## Supporting Information

Figure S1 The results of the replicated dose response runs of the cell lines in the BCell panel are plotted in separate panels.
(PDF)

Figure S2 Barplot of the GI_50_-values for the melphalan treatment of the 59 cell lines in the NCI60 panel.
(PDF)

Figure S3 Cross-validated accuracy for the NCI60 LDA analysis at various values of the parameter var.cutoff in nsFilter. The maximum accuracy is achieved when var.cutoff is equal to 0.38.
(PDF)

Figure S4 The minimum NCI60 MSPE achieved through cross-validating on *K* and *η* in SPLS for a variety of values for the var.cutoff. The smallest minimum MSPE is obtained with var.cutoff equal to 0.66.
(PDF)

Figure S5 The BCell MSPE for the SPLS regression with var.cutoff in nsFilter set equal to 0.74. This leads to the selection of *K = *2 hidden components and a sparsity parameter *η = *0.8193.
(PDF)

Figure S6 The BCell predicted resistance index vs. the measured resistance index. The left panel shows cross-validated predictions after filtering. The right panel shows cross-validated predictions where filtering is performed each time a cell line is left out.
(PDF)

Figure S7 Regularization paths for the optimal number of partial least squares components *K* = 3 and regularization parameter *η = *0.827 are shown. The true probesets have black lines and the false probesets are illustrated with grey lines. The sensitivity is 0.55, the specificity is 0.994 and the false discovery rate is 0.633.
(PDF)

Figure S8 Kaplan-Meier survival curves for OS based on the predicted NCI60 LDA classes. The logrank test comparing the survival curves results in a P-value of 0.05.
(PDF)

Figure S9 Kaplan-Meier survival curves for EFS based on the predicted NCI60 LDA classes. The logrank test comparing the survival curves results in a P-value of 0.3.
(PDF)

Figure S10 Kaplan-Meier survival curves for OS based on the BCell LDA classifier. The logrank test comparing the survival curves results in a P-value of 0.006.
(PDF)

Figure S11 Kaplan-Meier survival curves for EFS based on the BCell LDA classifier. The logrank test comparing the survival curves results in a P-value of 2e-05.
(PDF)

Figure S12 Kaplan-Meier survival curves for OS based on the two cell lines resistance index. The logrank test comparing the survival curves results in a P-value of 0.004.
(PDF)

Figure S13 Kaplan-Meier survival curves for EFS based on the two cell lines resistance index. The logrank test comparing the survival curves results in a P-value of 6e-04.
(PDF)

Figure S14 Kaplan-Meier survival curves for the Hummel data based on the BCell resistance index. The logrank test comparing the survival curves results in a P-value of 0.08.
(PDF)

Figure S15 Log relative hazard as a function of the BCell resistance index for the Hummel data. The P-value is the maximum likelihood ratio test for no RCS-association between log Relative Hazard and resistance index. The dashed lines represent 95% confidence intervals.
(PDF)

Table S1 Information about the BCell panel including the 18 cell lines used in the study.
(PDF)

Table S2 A summary of the 159 gene expressions used in the BCell LDA classifier.
(PDF)

Table S3 A summary of the gene expressions used in the two cell line resistance index.
(PDF)

Text S1 Sweave Document. This document contains a supporting Sweave document with text, source code, and figures.
(PDF)
