# Supplementary figures and images for: Generation of a Predictive Melphalan Resistance Index by Drug Screen of B-Cell Cancer Cell Lines
Source: PLoS One. 2011 Apr 29;6(4):e19322. doi: 10.1371/journal.pone.0019322 (PMC3084810; doi:10.1371/journal.pone.0019322)

Growth Inhibition for Cell Lines Treated with Melphalan

Replica

1 ▲      2 ◆      3 ■

$2^{-10}$   $2^{-5}$   $2^0$   $2^5$

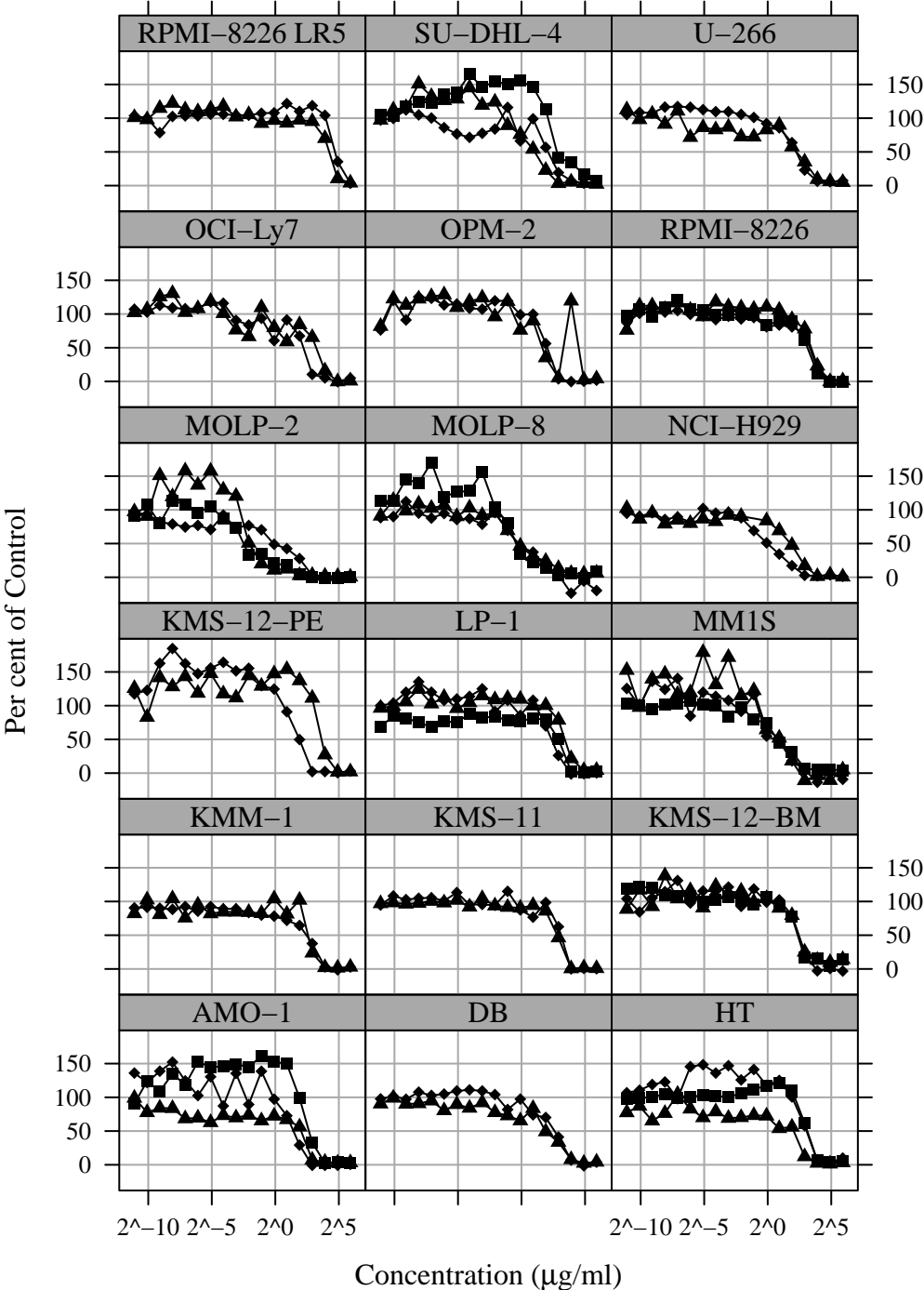

Supplement: Figure S1 — The results of the replicated dose response runs of the cell lines in the BCell panel are plotted in separate panels. (PDF) [file pone.0019322.s001.pdf]

# The NCI60 Melphalan Resistance Index

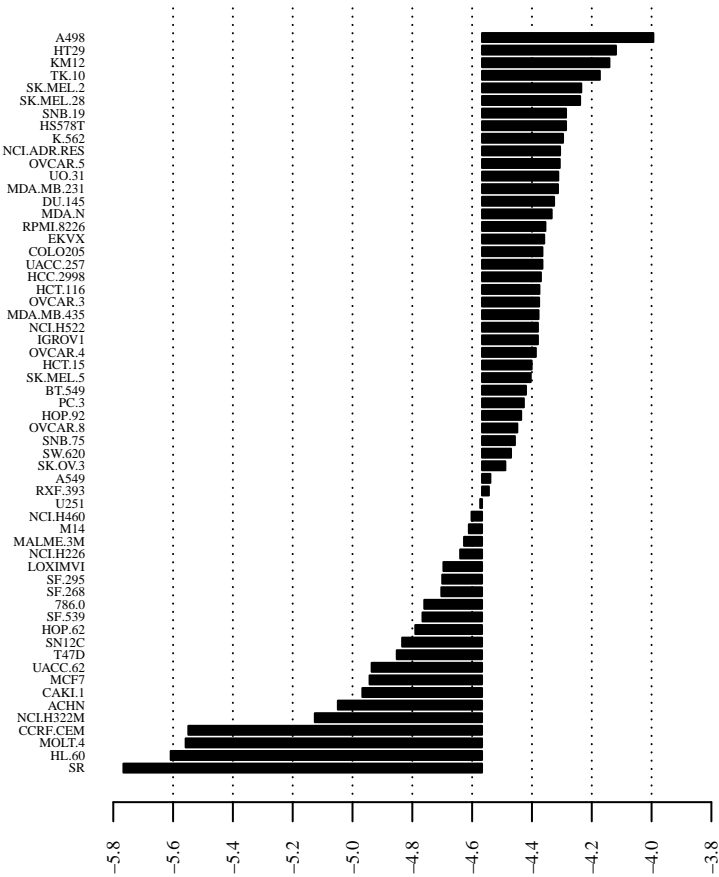

Log base 10 of the molar concentration

Supplement: Figure S2 — Barplot of the GI50-values for the melphalan treatment of the 59 cell lines in the NCI60 panel. (PDF) [file pone.0019322.s002.pdf]

# NCI60 Predicted Accuracy

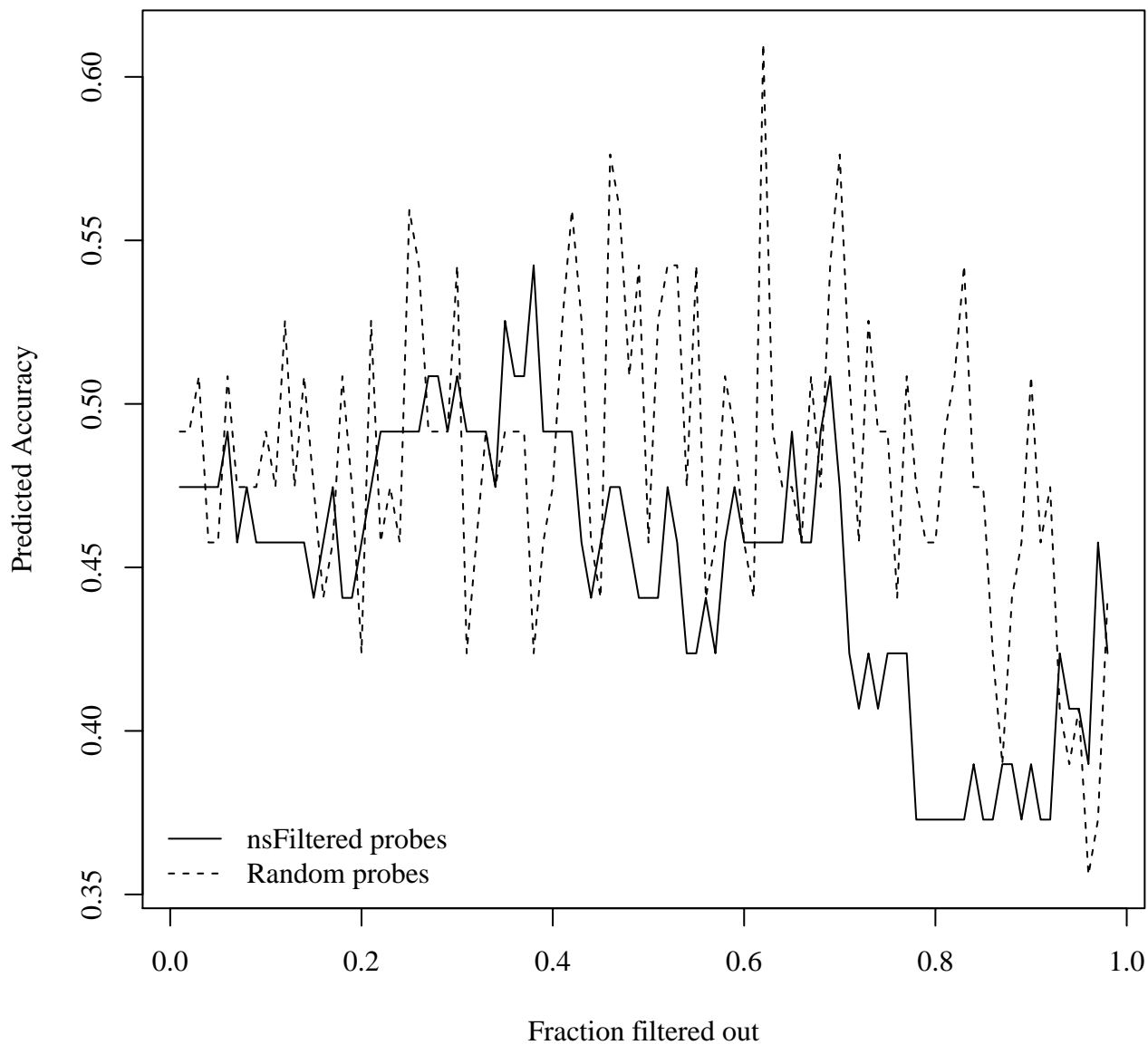

Supplement: Figure S3 — Cross-validated accuracy for the NCI60 LDA analysis at various values of the parameter var.cutoff in nsFilter. The maximum accuracy is achieved when var.cutoff is equal to 0.38. (PDF) [file pone.0019322.s003.pdf]

# NCI60 MSPE

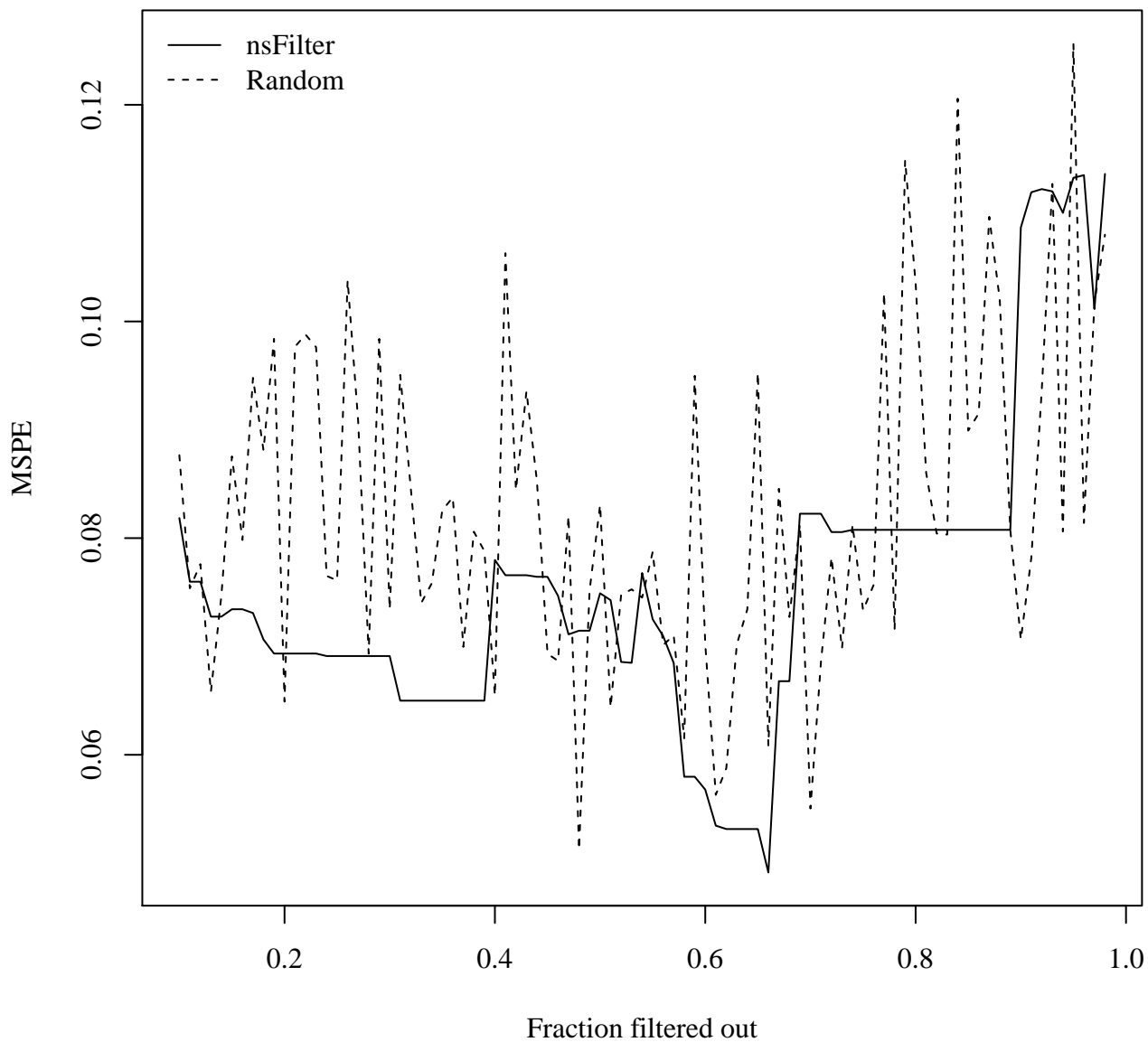

Supplement: Figure S4 — The minimum NCI60 MSPE achieved through cross-validating on K and η in SPLS for a variety of values for the var.cutoff. The smallest minimum MSPE is obtained with var.cutoff equal to 0.66. (PDF) [file pone.0019322.s004.pdf]

# BCell MSPE

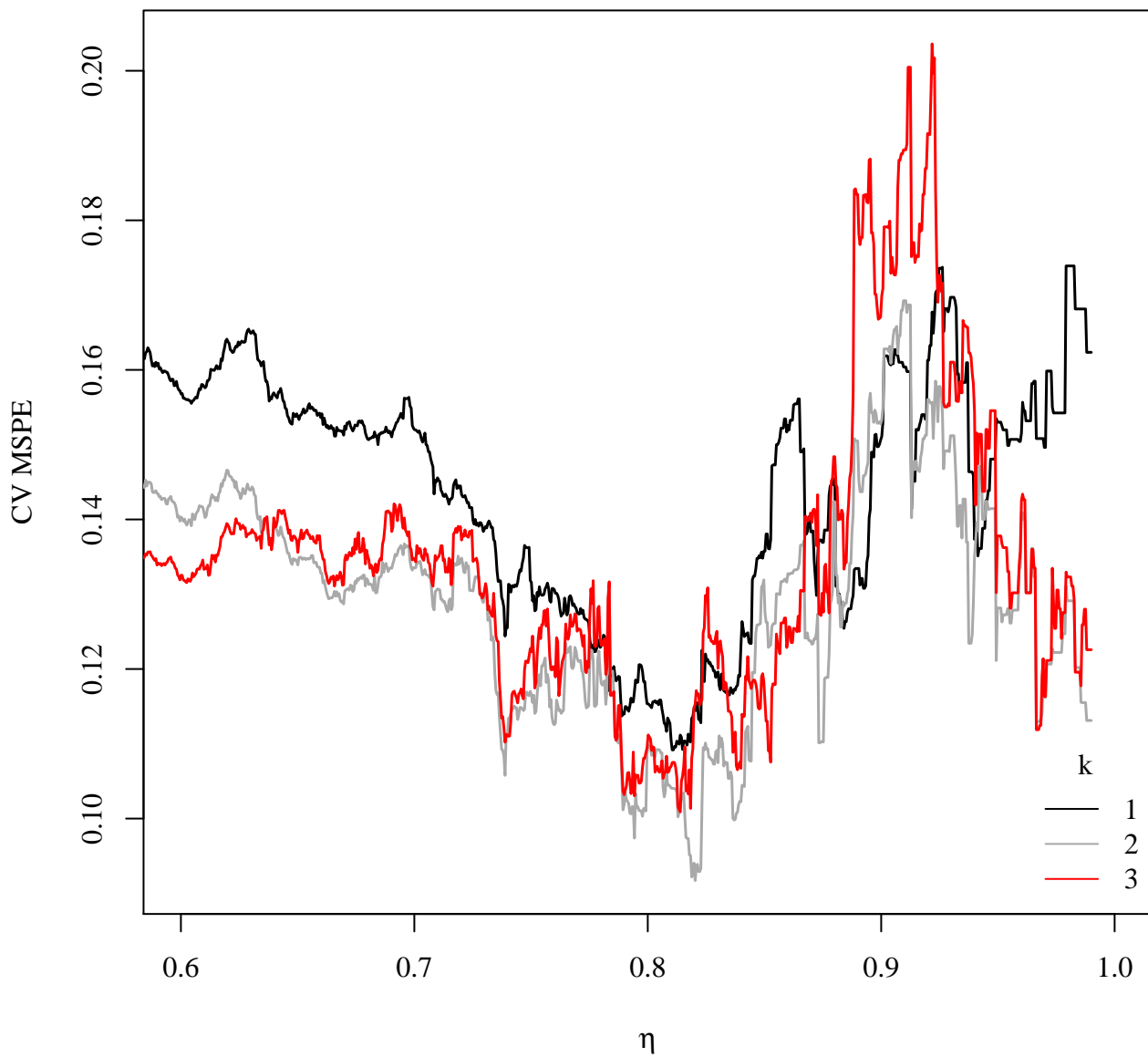

Supplement: Figure S5 — The BCell MSPE for the SPLS regression with var.cutoff in nsFilter set equal to 0.74. This leads to the selection of K = 2 hidden components and a sparsity parameter η = 0.8193. (PDF) [file pone.0019322.s005.pdf]

## CV BCell SPLS – Naive

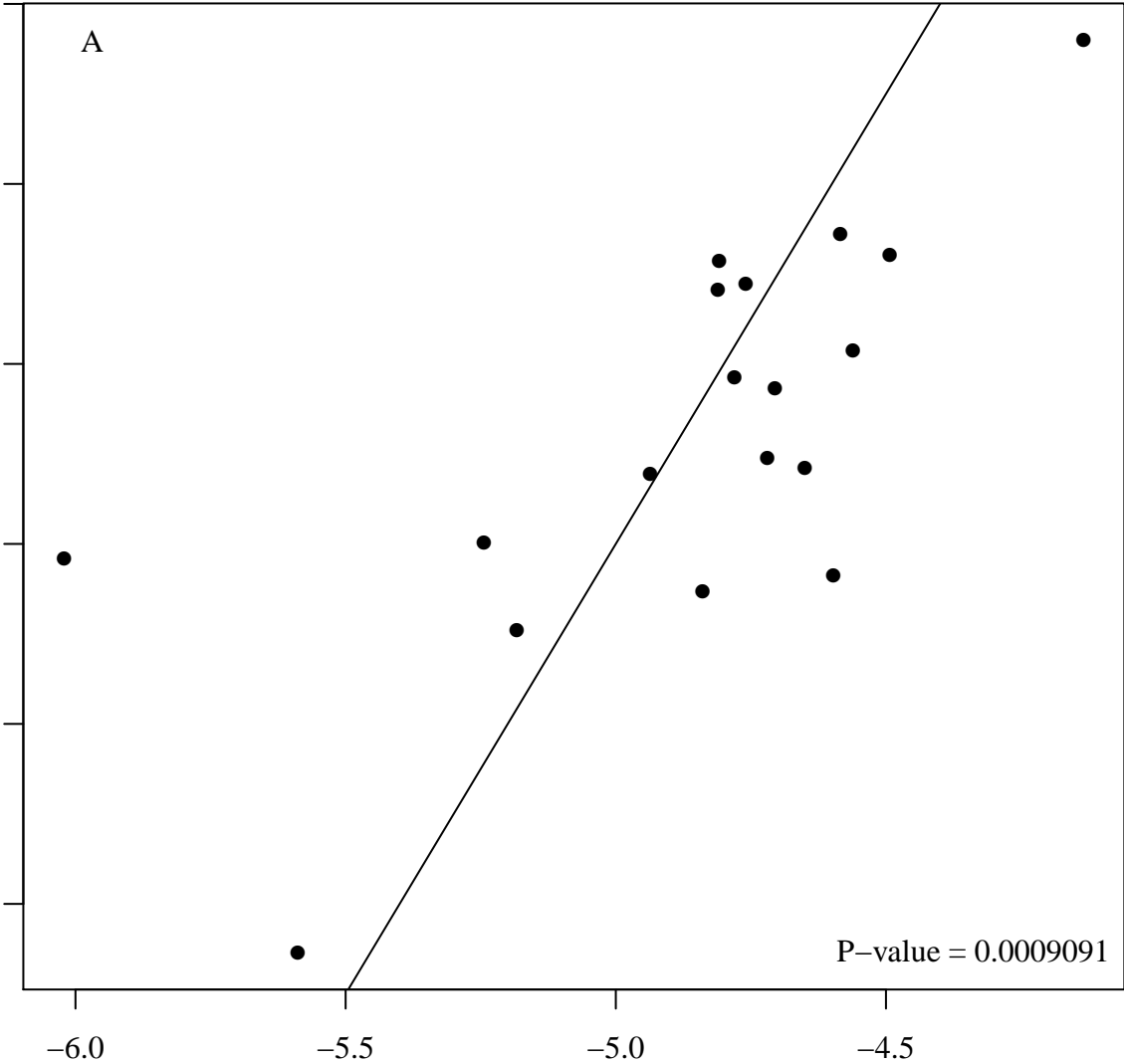

## CV BCell SPLS – with nsFilter

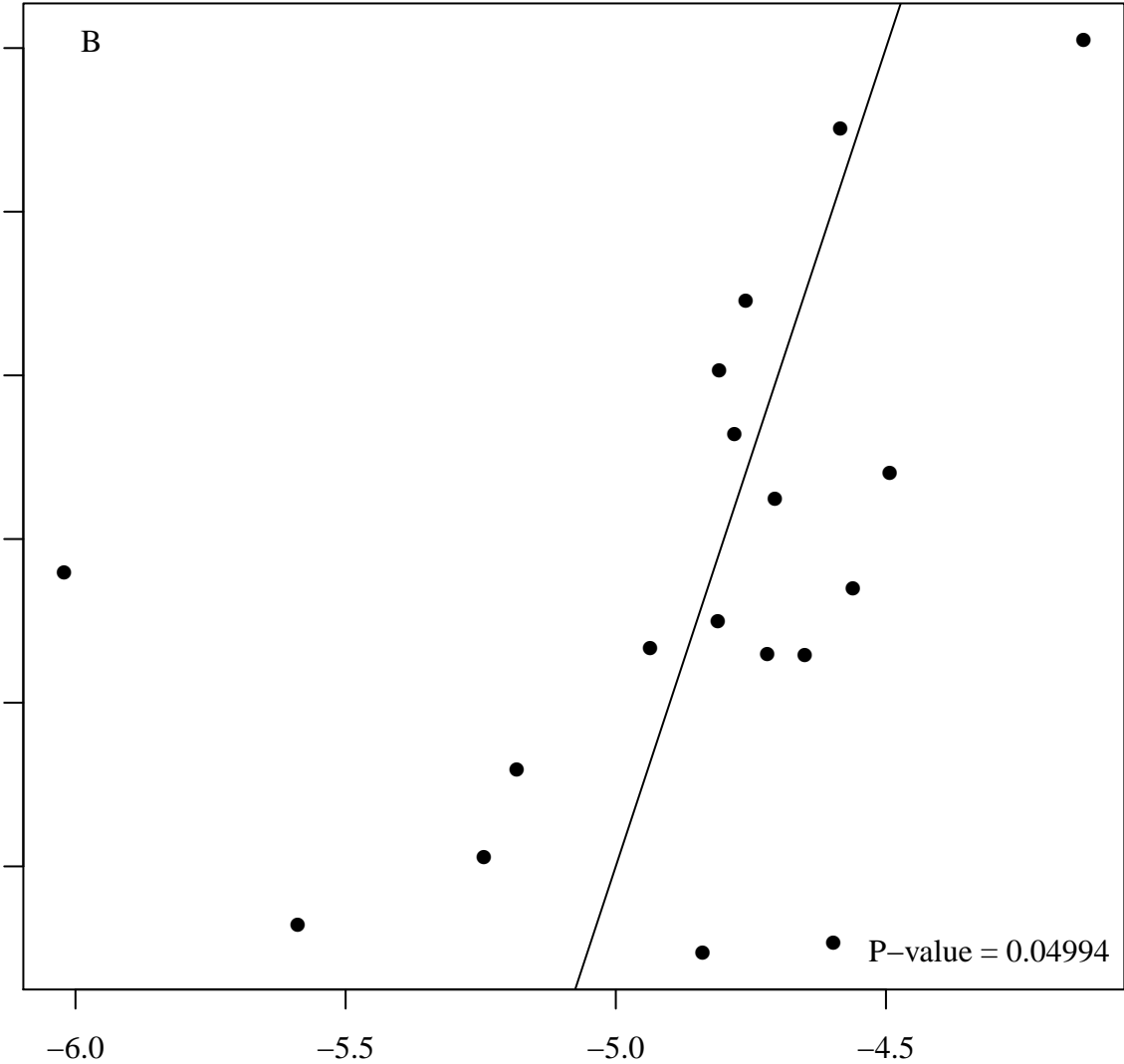

Supplement: Figure S6 — The BCell predicted resistance index vs. the measured resistance index. The left panel shows cross-validated predictions after filtering. The right panel shows cross-validated predictions where filtering is performed each time a cell line is left out. (PDF) [file pone.0019322.s006.pdf]

# Regularization Path

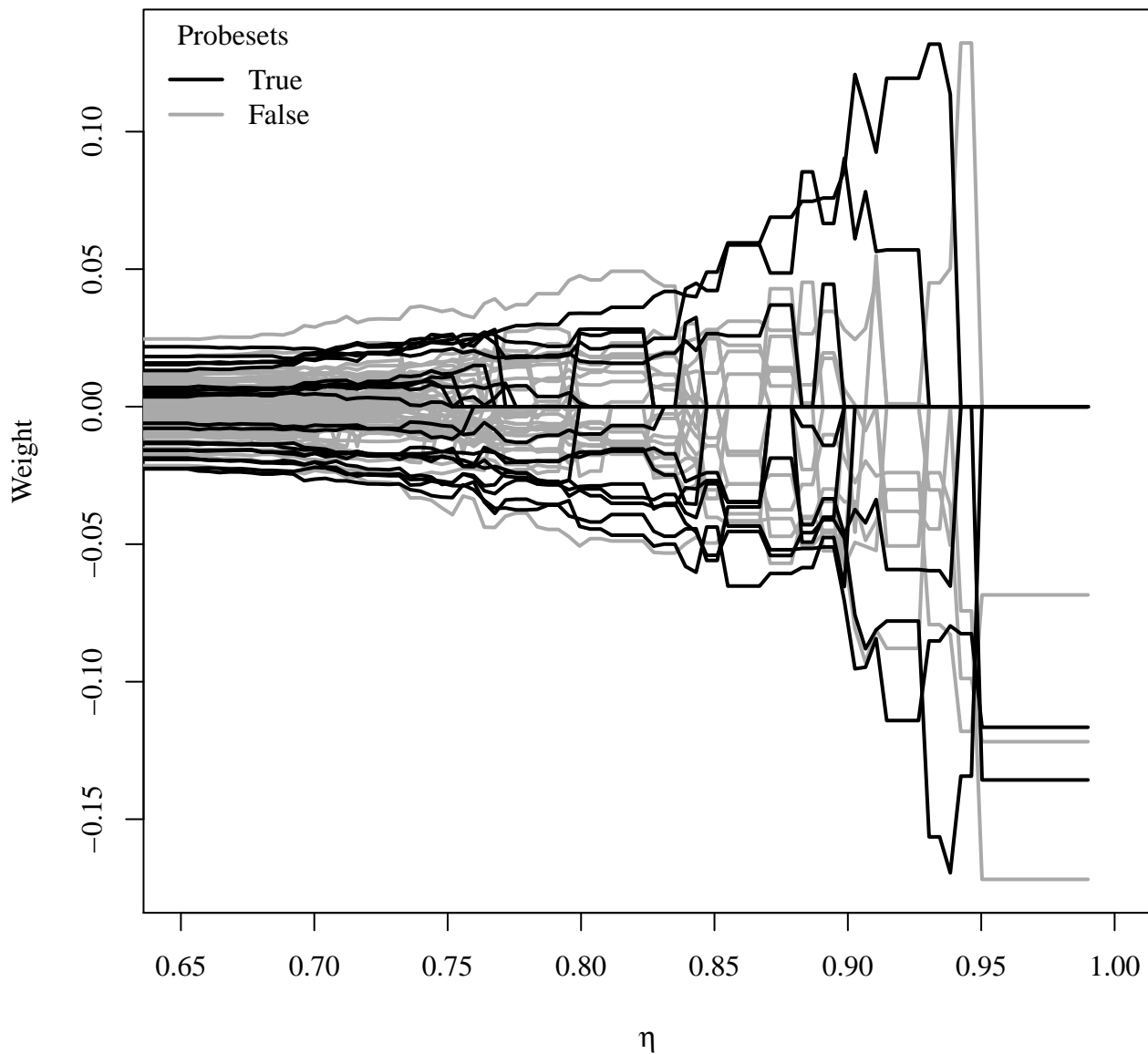

Supplement: Figure S7 — Regularization paths for the optimal number of partial least squares components K = 3 and regularization parameter η = 0.827 are shown. The true probesets have black lines and the false probesets are illustrated with grey lines. The sensitivity is 0.55, the specificity is 0.994 and the false discovery rate is 0.633. (PDF) [file pone.0019322.s007.pdf]

# Arkansas NCI60 Kaplan–Meier OS Curves

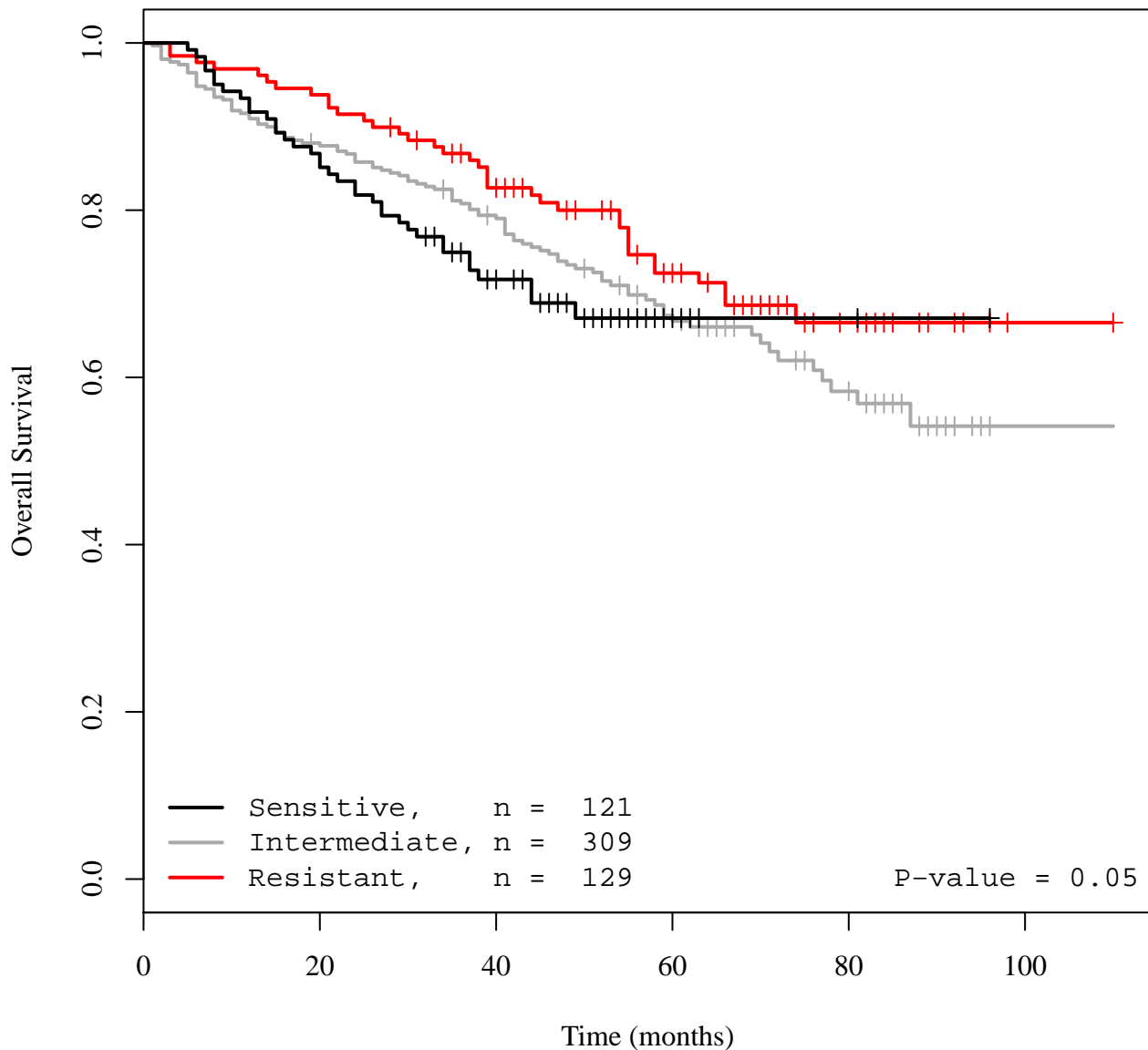

Supplement: Figure S8 — Kaplan-Meier survival curves for OS based on the predicted NCI60 LDA classes. The logrank test comparing the survival curves results in a P-value of 0.05. (PDF) [file pone.0019322.s008.pdf]

# Arkansas NCI60 Kaplan–Meier EFS Curves

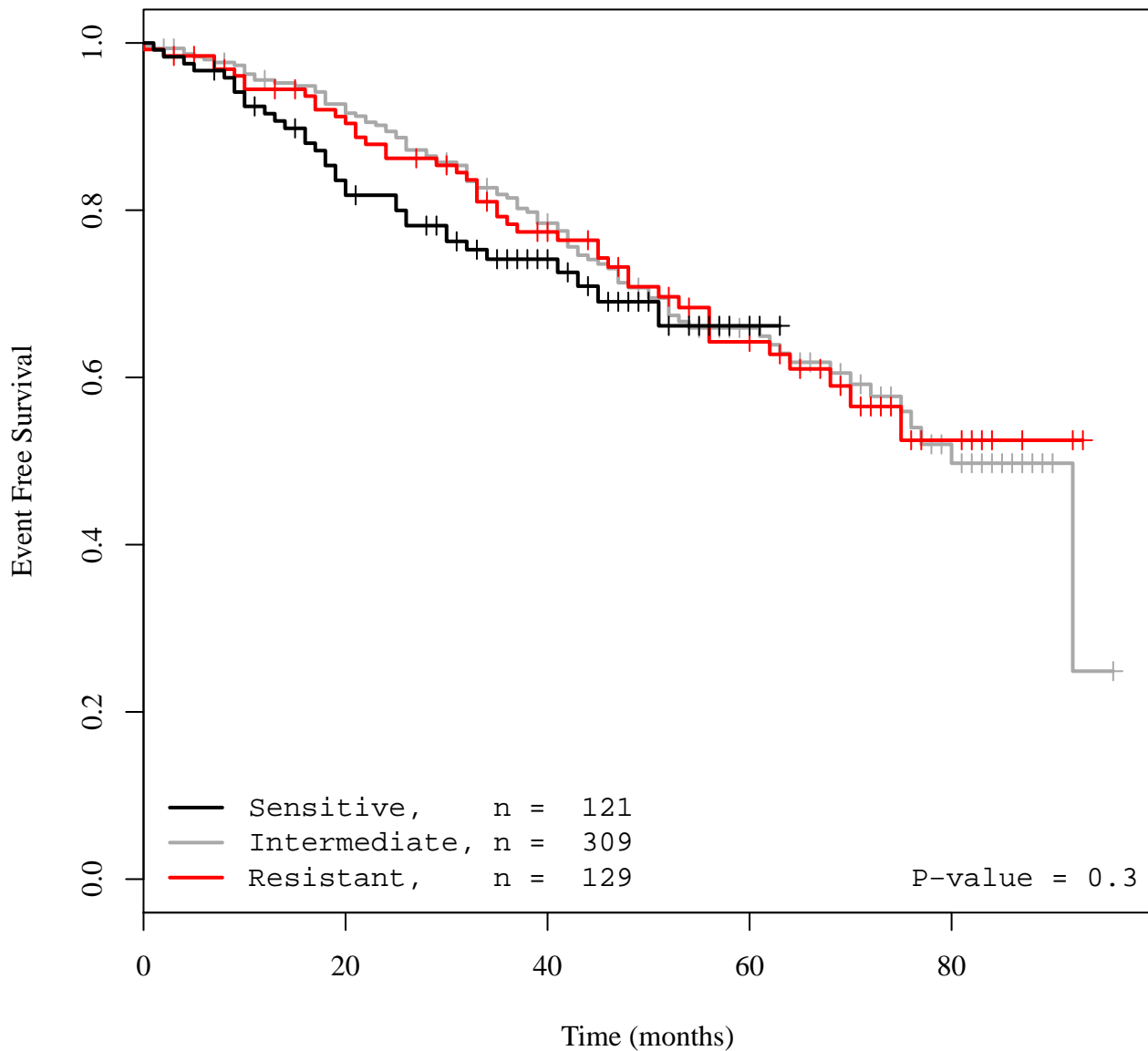

Supplement: Figure S9 — Kaplan-Meier survival curves for EFS based on the predicted NCI60 LDA classes. The logrank test comparing the survival curves results in a P-value of 0.3. (PDF) [file pone.0019322.s009.pdf]

# Arkansas BCell Kaplan–Meier OS Curves

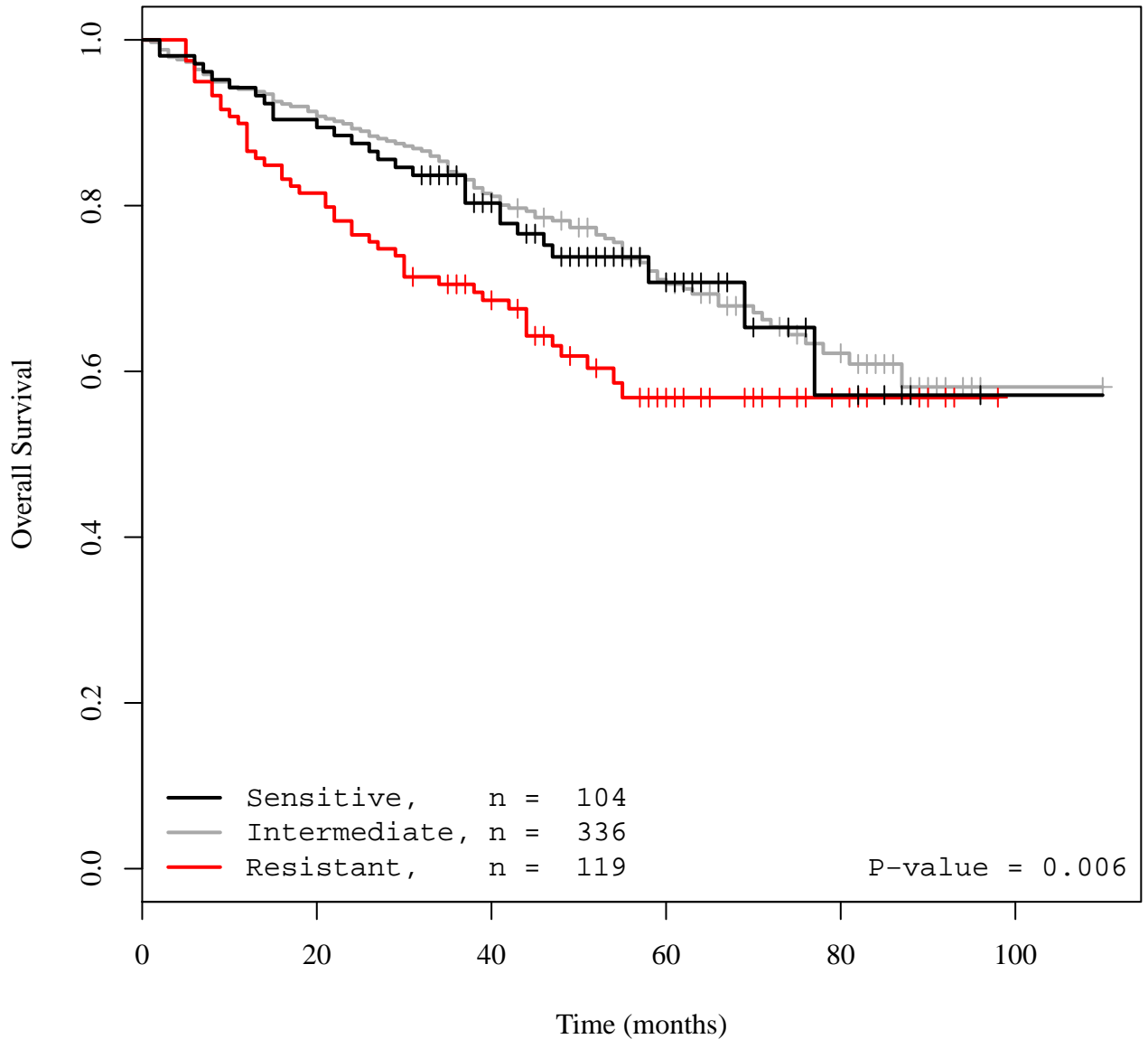

Supplement: Figure S10 — Kaplan-Meier survival curves for OS based on the BCell LDA classifier. The logrank test comparing the survival curves results in a P-value of 0.006. (PDF) [file pone.0019322.s010.pdf]

# Arkansas BCell Kaplan–Meier EFS Curves

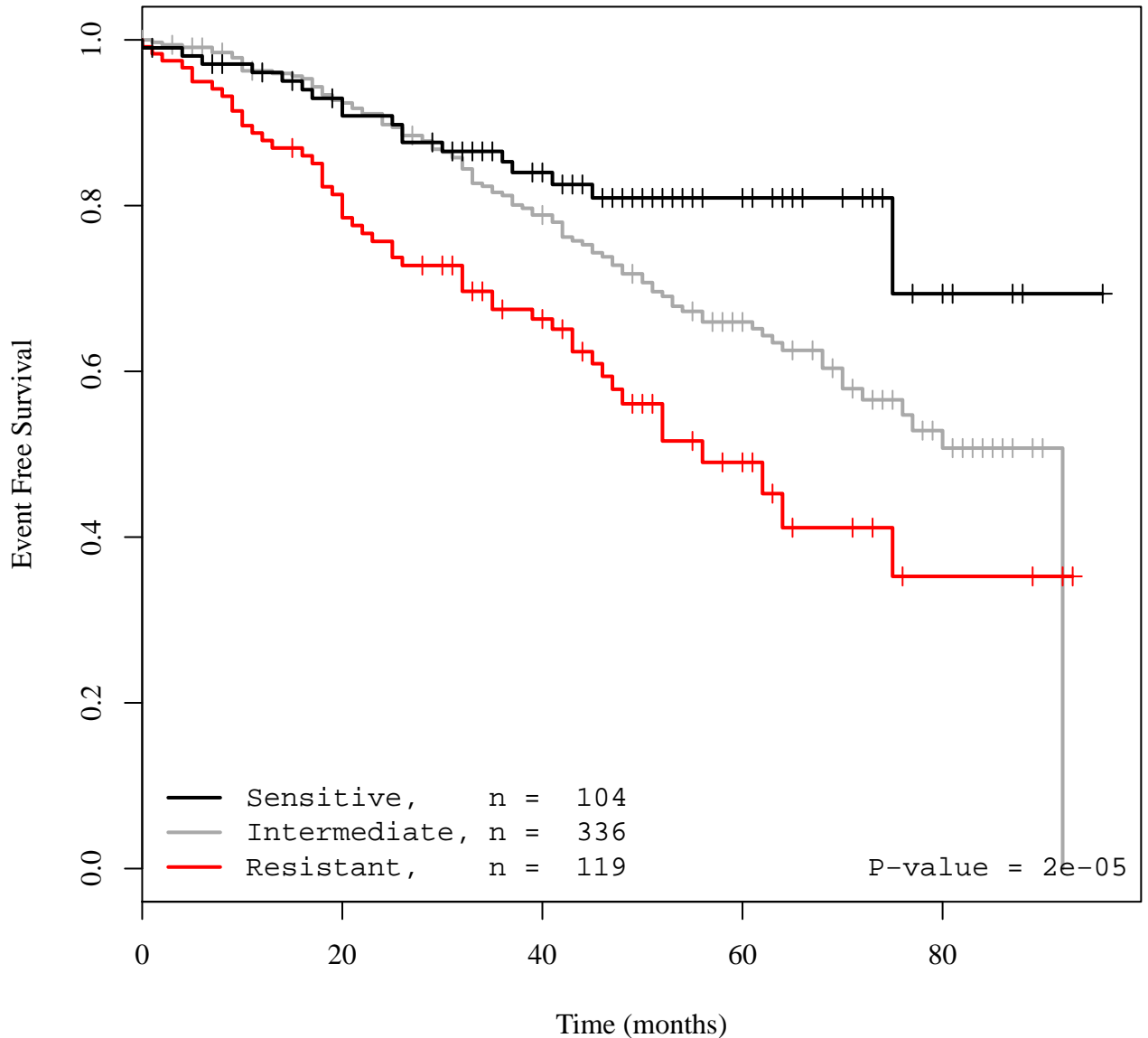

Supplement: Figure S11 — Kaplan-Meier survival curves for EFS based on the BCell LDA classifier. The logrank test comparing the survival curves results in a P-value of 2e-05. (PDF) [file pone.0019322.s011.pdf]

# Arkansas Kaplan–Meier OS curves

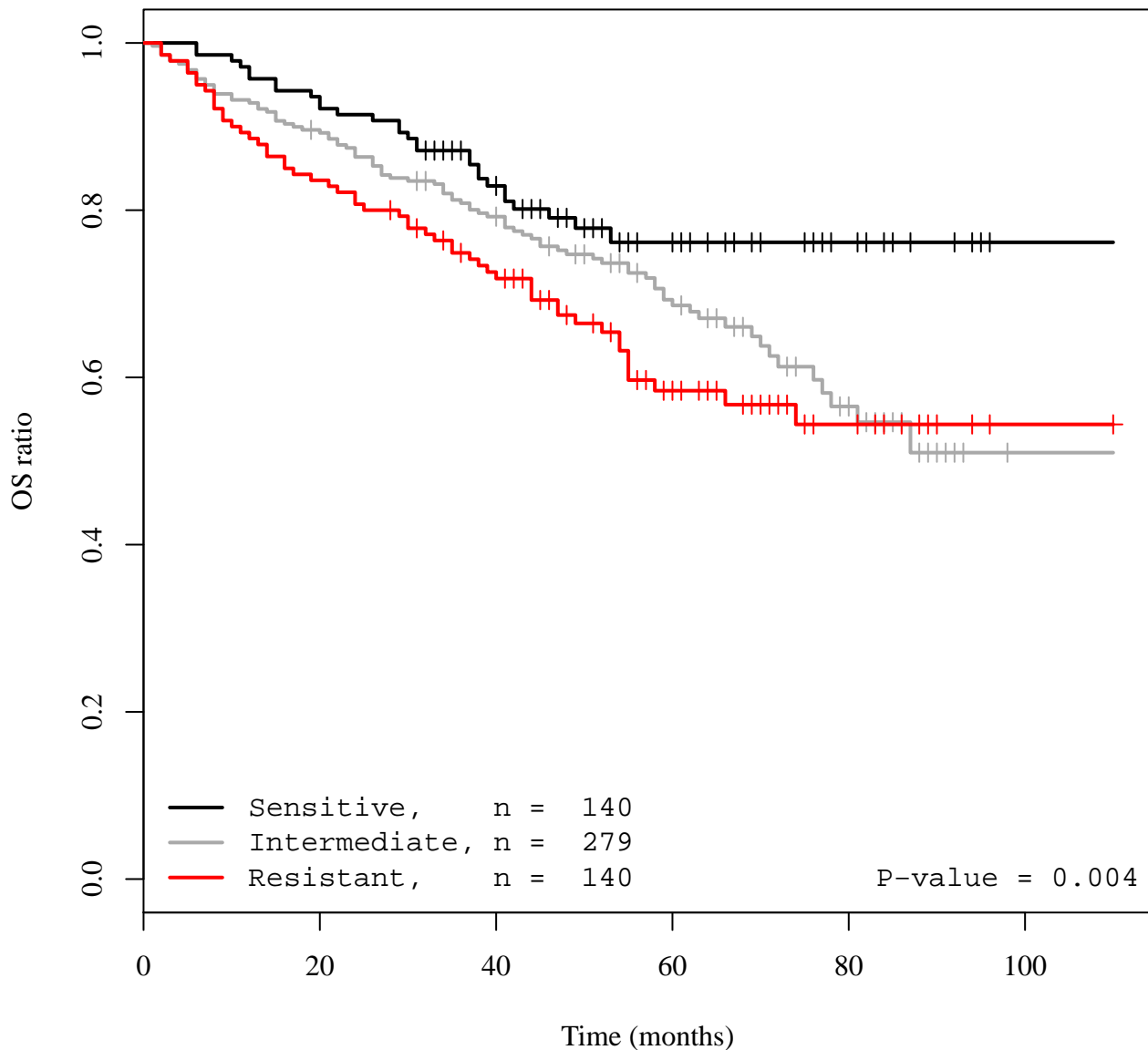

Supplement: Figure S12 — Kaplan-Meier survival curves for OS based on the two cell lines resistance index. The logrank test comparing the survival curves results in a P-value of 0.004. (PDF) [file pone.0019322.s012.pdf]

# Arkansas

## Kaplan–Meier EFS curves

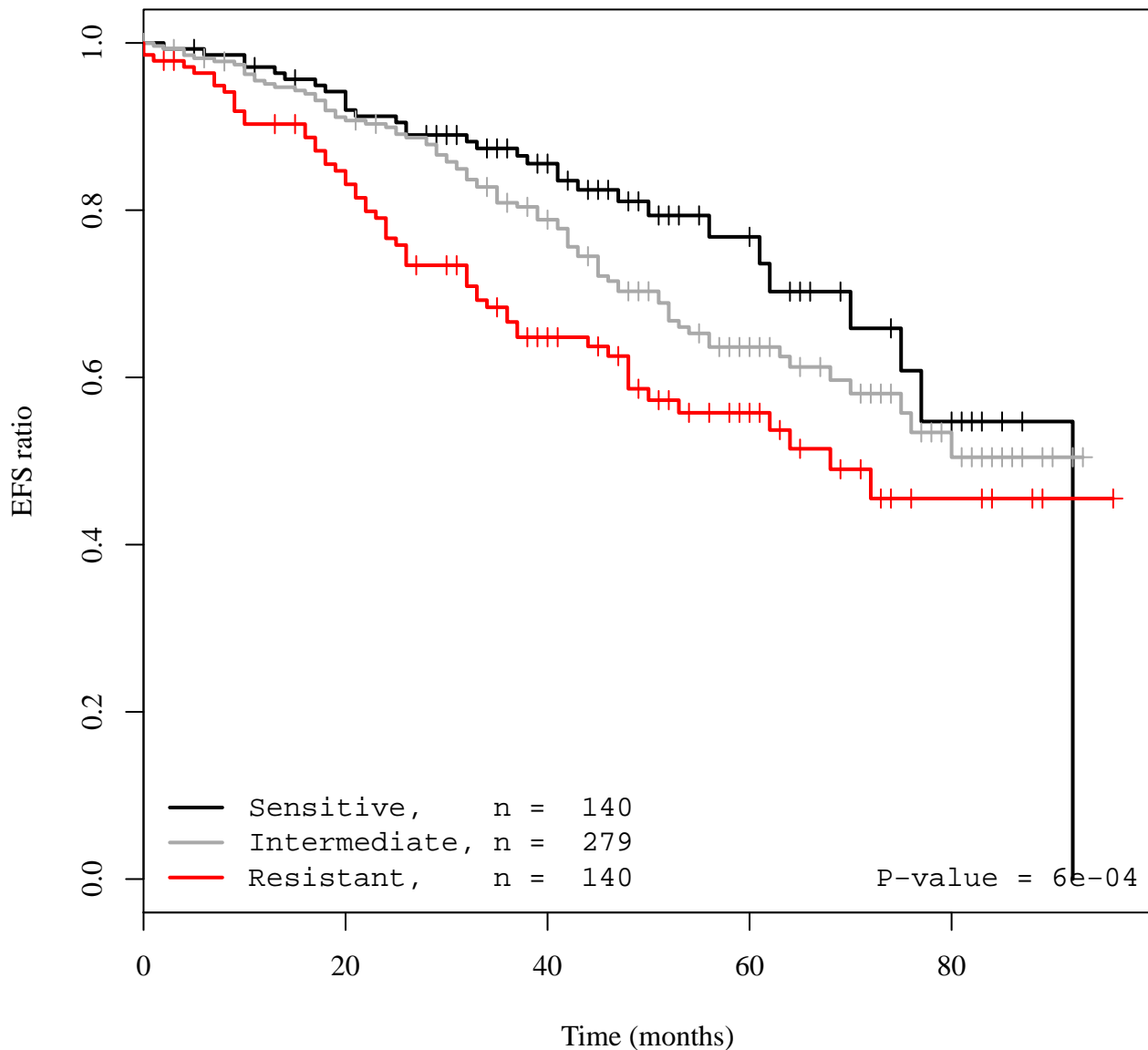

Supplement: Figure S13 — Kaplan-Meier survival curves for EFS based on the two cell lines resistance index. The logrank test comparing the survival curves results in a P-value of 6e-04. (PDF) [file pone.0019322.s013.pdf]

# Hummel

## BCell Kaplan–Meier OS curves

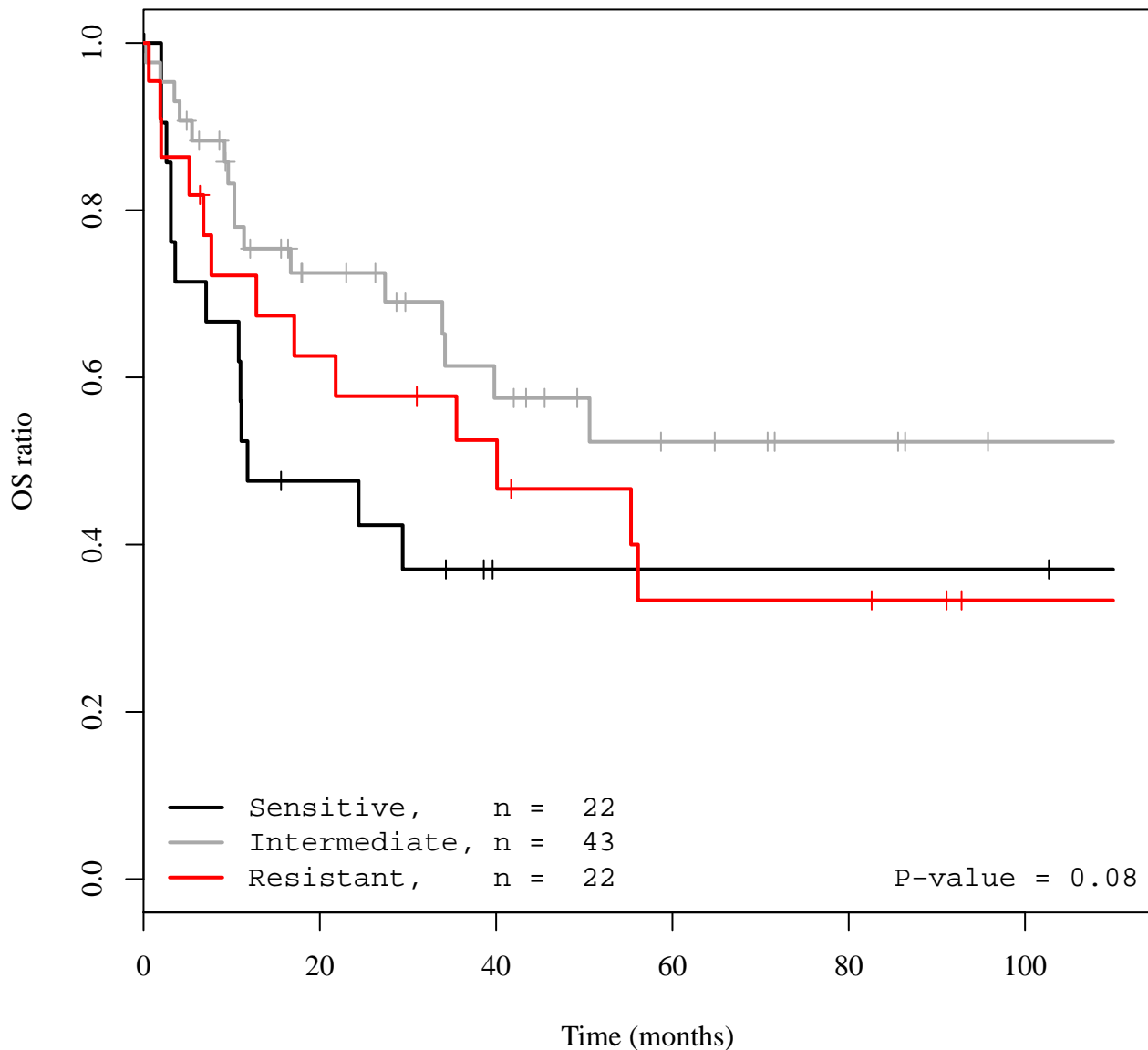

Supplement: Figure S14 — Kaplan-Meier survival curves for the Hummel data based on the BCell resistance index. The logrank test comparing the survival curves results in a P-value of 0.08. (PDF) [file pone.0019322.s014.pdf]

**Hummel**  
**BCell – OS Cox Proportional Hazards**

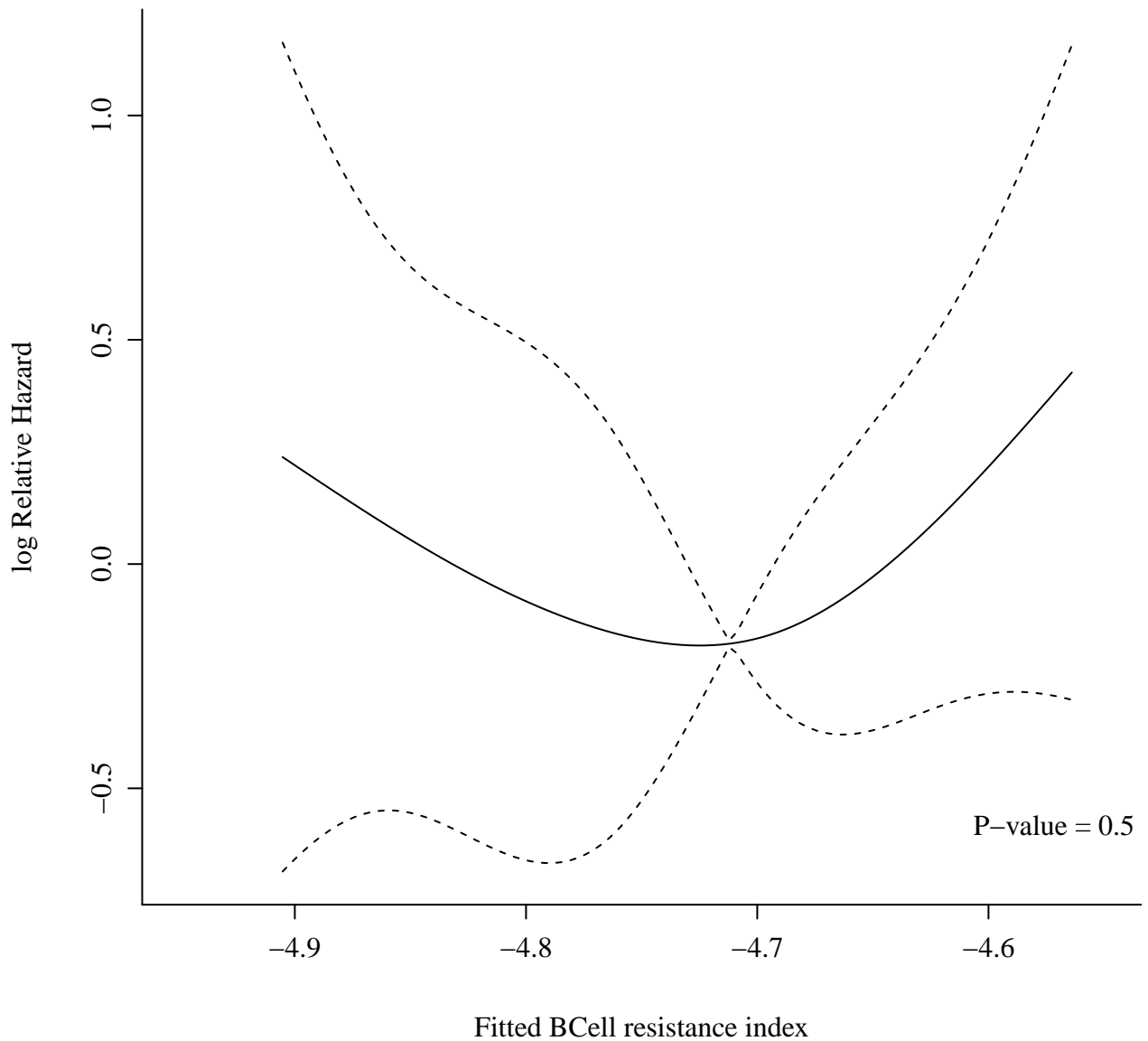

Supplement: Figure S15 — Log relative hazard as a function of the BCell resistance index for the Hummel data. The P-value is the maximum likelihood ratio test for no RCS-association between log Relative Hazard and resistance index. The dashed lines represent 95% confidence intervals. (PDF) [file pone.0019322.s015.pdf]
