# Supplementary material for: Generation of a Predictive Melphalan Resistance Index by Drug Screen of B-Cell Cancer Cell Lines
Source: PLoS One. 2011 Apr 29;6(4):e19322. doi: 10.1371/journal.pone.0019322 (PMC3084810; doi:10.1371/journal.pone.0019322)
Supplement: Table S1 — Information about the BCell panel including the 18 cell lines used in the study. (PDF) [file pone.0019322.s016.pdf]

Table S1. Information about the BCell panel.

Disease	Cell line	Medium	GI_{50}
MM	KMM-1	RPMI-1640, 10% FBS	-4.7198
MM	KMS-11	RPMI-1640, 10% FBS	-4.5848
MM	KMS-12-PE	RPMI-1640, 20% FBS	-4.5612
MM	KMS-12-BM	RPMI-1640, 20% FBS	-4.7596
MM	LP-1	IMDM, 10% FBS	-4.5975
MM	MM1S	RPMI-1640, 10% FBS	-5.2443
MM	MOLP-2	RPMI-1640, 10% FBS	-6.0210
MM	MOLP-8	RPMI-1640, 20% FBS	-5.5889
MM	NCI-H929	RPMI-1640, 20% FBS, 2 mM L-glutamine, 1 mM sodium-pyruvate	-5.1836
MM	OPM-2	RPMI-1640, 10% FBS	-4.9366
MM	RPMI-8226	RPMI-1640, 10% FBS	-4.4932
MM	RPMI-8226 LR5	RPMI-1640, 10% FBS, 5 μM melphalan was added once a week	-4.1343
MM	U-266	RPMI-1640, 10% FBS	-4.8088
PC	AMO-1	RPMI-1640, 20% FBS	-4.8391
DLBCL	DB	RPMI-1640, 20% FBS	-4.7802
DLBCL	HT	RPMI-1640, 10% FBS	-4.6502
DLBCL	OCI-Ly7	RPMI-1640, 10% FBS	-4.7053
DLBCL	SU-DHL-4	RPMI-1640, 10% FBS	-4.8111

Abbreviations: multiple myeloma (MM), plasmacytoma (PC), diffuse large B-cell lymphoma (DLBCL), fetal bovine serum (FBS). GI_{50}-values are on $\log_{10}$ μM/ml scale.
