# Supplementary material for: Generation of a Predictive Melphalan Resistance Index by Drug Screen of B-Cell Cancer Cell Lines
Source: PLoS One. 2011 Apr 29;6(4):e19322. doi: 10.1371/journal.pone.0019322 (PMC3084810; doi:10.1371/journal.pone.0019322)
Supplement: Table S2 — A summary of the 159 gene expressions used in the BCell LDA classifier. (PDF) [file pone.0019322.s017.pdf]

Table S2. A summary of the 159 gene expressions used in the BCell LDA classifier.

Probesets	Symbol	AveExpr	F	P.Value	adj.P.Val
209310_s_at	CASP4	2.282e-16	6.343	0.001759	0.1572
221004_s_at	ITM2C	-2.722e-16	6.245	0.00194	0.1572
221912_s_at	CCDC28B	-1.258e-16	6.139	0.002158	0.1572
209735_at	ABCG2	-8.481e-17	5.973	0.002545	0.1572
221297_at	GPRC5D	2.564e-17	5.863	0.002843	0.1572
206060_s_at	PTPN22	1.627e-16	5.853	0.002872	0.1572
210145_at	PLA2G4A	-1.388e-17	5.676	0.003427	0.1572
218330_s_at	NAV2	-1.496e-16	5.673	0.003438	0.1572
209568_s_at	RGL1	-2.619e-16	5.652	0.003512	0.1572
212543_at	AIM1	1.103e-16	5.51	0.004046	0.1572
200602_at	APP	5.089e-17	5.246	0.005266	0.1572
221268_s_at	SGPP1	1.588e-16	5.236	0.00532	0.1572
205807_s_at	TUFT1	-1.82e-16	5.179	0.005635	0.1572
203758_at	CTSO	6.014e-17	5.131	0.005908	0.1572
204552_at	INPP4A	2.806e-16	5.117	0.005996	0.1572
201125_s_at	ITGB5	-4.715e-16	5.089	0.006163	0.1572
210942_s_at	ST3GAL6	-5.204e-17	5.081	0.006216	0.1572
206121_at	AMPD1	-7.859e-17	5.053	0.006393	0.1572
222258_s_at	SH3BP4	-1.218e-16	5.032	0.006529	0.1572
219572_at	CADPS2	-3.547e-17	4.992	0.006795	0.1572
211368_s_at	CASP1	4.163e-17	4.929	0.007237	0.1572
34210_at	CD52	-5.551e-17	4.916	0.007325	0.1572
202551_s_at	CRIM1	-1.349e-16	4.91	0.007374	0.1572
202947_s_at	GYPC	-1.588e-16	4.866	0.007705	0.1572
212886_at	CCDC69	3.915e-16	4.857	0.007771	0.1572
205016_at	TGFA	-6.818e-17	4.85	0.007827	0.1572
209163_at	CYB561	4.795e-18	4.827	0.008014	0.1572
219191_s_at	BIN2	1.11e-16	4.81	0.008145	0.1572
210279_at	GPR18	-1.388e-16	4.801	0.008222	0.1572
218793_s_at	SCML1	1.295e-16	4.77	0.008479	0.1572
205110_s_at	FGF13	2.467e-17	4.755	0.008606	0.1572
214023_x_at	TUBB2B	-1.068e-16	4.646	0.009601	0.1572
202609_at	EPS8	-2.66e-17	4.643	0.009632	0.1572
221526_x_at	PARD3	1.665e-16	4.634	0.009717	0.1572
221645_s_at	ZNF83	7.922e-17	4.612	0.009929	0.1572
221704_s_at	VPS37B	-2.321e-16	4.585	0.0102	0.1572
219221_at	ZBTB38	3.296e-16	4.567	0.01039	0.1572
212715_s_at	MICAL3	1.759e-16	4.555	0.01051	0.1572
213415_at	CLIC2	7.71e-17	4.534	0.01073	0.1572
222317_at	PDE3B	5.474e-17	4.52	0.01088	0.1572
206698_at	XK	-1.48e-16	4.508	0.01102	0.1572
202732_at	PKIG	-2.313e-18	4.485	0.01128	0.1572
200660_at	S100A11	2.012e-16	4.463	0.01153	0.1572
218723_s_at	C13orf15	-7.556e-17	4.444	0.01175	0.1572
204730_at	RIMS3	-2.506e-16	4.44	0.0118	0.1572

Table 1: *(continued)*

Probesets	Symbol	AveExpr	F	P.Value	adj.P.Val
212588_at	PTPRC	-9.252e-17	4.381	0.01251	0.1572
204589_at	NUAK1	-1.82e-16	4.379	0.01254	0.1572
207826_s_at	ID3	-3.77e-16	4.369	0.01266	0.1572
212195_at	IL6ST	1.261e-16	4.364	0.01273	0.1572
222150_s_at	PION	-9.714e-17	4.363	0.01273	0.1572
218080_x_at	FAF1	5.158e-16	4.352	0.01288	0.1572
212724_at	RND3	-1.542e-16	4.34	0.01303	0.1572
203932_at	HLA-DMB	-2.105e-16	4.332	0.01314	0.1572
214890_s_at	FAM149A	-2.393e-16	4.276	0.0139	0.1612
201301_s_at	ANXA4	-3.847e-16	4.256	0.01418	0.1612
213325_at	PVRL3	-2.409e-16	4.228	0.01458	0.1612
219159_s_at	SLAMF7	-8.635e-17	4.22	0.0147	0.1612
221727_at	SUB1	2.799e-16	4.204	0.01494	0.1612
209829_at	FAM65B	9.56e-17	4.179	0.01531	0.1612
202371_at	TCEAL4	-2.066e-16	4.177	0.01535	0.1612
221942_s_at	GUCY1A3	1.581e-16	4.165	0.01553	0.1612
201998_at	ST6GAL1	-2.891e-17	4.14	0.01592	0.1612
202746_at	ITM2A	3.084e-18	4.134	0.01602	0.1612
207307_at	HTR2C	1.673e-16	4.104	0.01651	0.1635
201063_at	RCN1	5.86e-17	4.041	0.01758	0.1677
204613_at	PLCG2	4.24e-18	4.024	0.01789	0.1677
214608_s_at	EYA1	-3.392e-17	4.019	0.01798	0.1677
202096_s_at	TSPO	-4.572e-16	3.979	0.01871	0.1677
203795_s_at	BCL7A	-1.542e-16	3.955	0.01916	0.1677
212192_at	KCTD12	-1.295e-16	3.948	0.01929	0.1677
213502_x_at	LOC91316	1.465e-16	3.938	0.01948	0.1677
202177_at	GAS6	-2.853e-17	3.938	0.01949	0.1677
209348_s_at	MAF	-4.626e-17	3.935	0.01955	0.1677
218409_s_at	DNAJC1	2.467e-17	3.929	0.01966	0.1677
205945_at	IL6R	-2.082e-16	3.92	0.01984	0.1677
200706_s_at	LITAF	-4.179e-16	3.883	0.02058	0.1685
212442_s_at	LASS6	1.727e-16	3.882	0.02061	0.1685
213245_at	ADCY1	-1.295e-16	3.876	0.02073	0.1685
201681_s_at	DLG5	1.798e-16	3.828	0.02176	0.1709
201212_at	LGMN	-1.557e-16	3.785	0.02271	0.1709
201841_s_at	HSPB1	-4.125e-16	3.765	0.02316	0.1709
202933_s_at	YES1	1.773e-16	3.751	0.0235	0.1709
206641_at	TNFRSF17	2.603e-16	3.745	0.02364	0.1709
203986_at	STBD1	-1.226e-16	3.741	0.02373	0.1709
208892_s_at	DUSP6	-7.864e-17	3.731	0.02396	0.1709
200824_at	GSTP1	-1.11e-16	3.712	0.02443	0.1709
205229_s_at	COCH	1.382e-16	3.685	0.02509	0.1709
219696_at	DENND1B	-3.369e-16	3.681	0.0252	0.1709
217995_at	SQRDL	-1.661e-16	3.678	0.02526	0.1709
209198_s_at	SYT11	1.885e-16	3.671	0.02546	0.1709
205718_at	ITGB7	-3.115e-16	3.661	0.02571	0.1709

Table 1: *(continued)*

Probesets	Symbol	AveExpr	F	P.Value	adj.P.Val
203476_at	TPBG	5.86e-17	3.659	0.02575	0.1709
204960_at	PTPRCAP	2.205e-16	3.657	0.0258	0.1709
203411_s_at	LMNA	2.209e-16	3.649	0.02601	0.1709
200839_s_at	CTSB	1.577e-16	3.625	0.02665	0.1709
209340_at	UAP1	-3.3e-16	3.624	0.02666	0.1709
218404_at	SNX10	-1.847e-16	3.621	0.02677	0.1709
219010_at	C1orf106	-3.481e-16	3.615	0.02691	0.1709
219551_at	EAF2	5.196e-16	3.612	0.027	0.1709
205943_at	TDO2	-1.279e-16	3.61	0.02705	0.1709
212096_s_at	MTUS1	1.789e-16	3.594	0.02748	0.1709
207039_at	CDKN2A	-2.101e-17	3.594	0.02749	0.1709
204254_s_at	VDR	-2.39e-17	3.565	0.02829	0.1741
202242_at	TSPAN7	2.136e-16	3.543	0.02892	0.1763
202136_at	ZMYND11	-2.375e-16	3.522	0.02955	0.1764
218847_at	IGF2BP2	1.342e-16	3.52	0.02961	0.1764
212843_at	NCAM1	-3.286e-17	3.499	0.03021	0.1764
218718_at	PDGFC	-9.252e-18	3.495	0.03035	0.1764
201647_s_at	SCARB2	1.519e-16	3.48	0.03079	0.1764
200697_at	HK1	3.84e-16	3.463	0.03134	0.1764
209619_at	CD74	-1.058e-16	3.458	0.0315	0.1764
203397_s_at	GALNT3	-8.481e-17	3.457	0.03152	0.1764
201828_x_at	FAM127A	-5.089e-16	3.451	0.03172	0.1764
60474_at	FERMT1	-1.164e-16	3.441	0.03202	0.1764
221122_at	HRASLS2	2.567e-16	3.433	0.03229	0.1764
204688_at	SGCE	-3.701e-16	3.431	0.03236	0.1764
212097_at	CAV1	-7.71e-18	3.41	0.03304	0.1764
217967_s_at	FAM129A	1.664e-16	3.41	0.03306	0.1764
202946_s_at	BTBD3	-7.633e-17	3.407	0.03314	0.1764
206632_s_at	APOBEC3B	-2.018e-16	3.393	0.03362	0.1764
219003_s_at	MANEA	-3.192e-16	3.367	0.03448	0.1764
205903_s_at	KCNN3	-3.3e-16	3.366	0.03452	0.1764
206700_s_at	KDM5D	1.419e-16	3.363	0.03464	0.1764
218974_at	SOBP	-2.213e-16	3.359	0.03478	0.1764
206609_at	MAGEC1	1.288e-16	3.359	0.03479	0.1764
205297_s_at	CD79B	-1.766e-16	3.317	0.03625	0.1802
205933_at	SETBP1	1.195e-17	3.314	0.03635	0.1802
202388_at	RGS2	-2.22e-16	3.31	0.03652	0.1802
201462_at	SCRN1	-4.163e-17	3.293	0.03716	0.1802
203167_at	TIMP2	4.318e-17	3.284	0.03747	0.1802
205098_at	CCR1	-9.252e-17	3.277	0.03772	0.1802
219014_at	PLAC8	1.48e-16	3.272	0.03792	0.1802
214452_at	BCAT1	-1.593e-16	3.271	0.03795	0.1802
202017_at	EPHX1	-1.218e-16	3.268	0.03808	0.1802
210473_s_at	GPR125	-1.511e-16	3.247	0.0389	0.1827
204409_s_at	EIF1AY	1.234e-17	3.233	0.03944	0.183
220603_s_at	MCTP2	6.63e-17	3.225	0.03975	0.183

Table 1: *(continued)*

Probesets	Symbol	AveExpr	F	P.Value	adj.P.Val
212345_s_at	CREB3L2	-2.831e-16	3.208	0.04044	0.183
203710_at	ITPR1	-5.551e-17	3.208	0.04045	0.183
200999_s_at	CKAP4	-1.82e-16	3.203	0.04066	0.183
204923_at	SASH3	-1.542e-17	3.202	0.04069	0.183
204364_s_at	REEP1	-1.739e-16	3.155	0.04263	0.1894
218618_s_at	FNDC3B	3.523e-16	3.153	0.04272	0.1894
203324_s_at	CAV2	-5.86e-17	3.143	0.04316	0.1895
211105_s_at	NFATC1	-5.86e-17	3.136	0.04346	0.1895
220306_at	FAM46C	-4.042e-16	3.132	0.04364	0.1895
213135_at	TIAM1	9.213e-17	3.118	0.04424	0.1896
206624_at	USP9Y	2.56e-16	3.116	0.04432	0.1896
211373_s_at	PSEN2	9.059e-17	3.106	0.04478	0.1896
206167_s_at	ARHGAP6	-3.547e-17	3.096	0.04522	0.1896
201858_s_at	SRGN	6.091e-17	3.095	0.04529	0.1896
201540_at	FHL1	-1.735e-16	3.088	0.04557	0.1896
218450_at	HEBP1	-1.156e-16	3.085	0.04575	0.1896
213913_s_at	TBC1D30	-1.372e-16	3.067	0.04654	0.1916
217551_at	OR7E14P	-1.11e-16	3.039	0.04789	0.1946
221666_s_at	PYCARD	5.705e-17	3.039	0.04789	0.1946
214131_at	CYorf15B	-6.168e-17	3.032	0.04822	0.1947
219812_at	PVRIG	1.372e-16	3.005	0.04954	0.1983
213478_at	KAZ	2.282e-16	2.999	0.04985	0.1983
