# Supplementary material for: Generation of a Predictive Melphalan Resistance Index by Drug Screen of B-Cell Cancer Cell Lines
Source: PLoS One. 2011 Apr 29;6(4):e19322. doi: 10.1371/journal.pone.0019322 (PMC3084810; doi:10.1371/journal.pone.0019322)
Supplement: Table S3 — A summary of the gene expressions used in the two cell line resistance index. (PDF) [file pone.0019322.s018.pdf]

Table S3. Table of the gene expressions used in the two cell line resistance index.

U133a ID	Gene Symbol	Weight	U133a ID	Gene Symbol	Weight
205174_s_at	QPCT	-7.893	210942_s_at	ST3GAL6	5.596
205590_at	RASGRP1	-7.89	215189_at	KRT86	5.599
213831_at	HLA-DQA1	-7.591	212473_s_at	MICAL2	5.687
212122_at	RHOQ	-7.303	209735_at	ABCG2	5.69
210587_at	INHBE	-7.032	218502_s_at	TRPS1	5.717
39248_at	AQP3	-6.988	214023_x_at	TUBB2B	5.721
205990_s_at	WNT5A	-6.854	212724_at	RND3	5.752
202609_at	EPS8	-6.797	203474_at	IQGAP2	5.767
219159_s_at	SLAMF7	-6.793	204014_at	DUSP4	5.772
213938_at	ERC2	-6.724	213524_s_at	G0S2	5.774
204118_at	CD48	-6.706	217996_at	PHLDA1	5.797
207191_s_at	ISLR	-6.422	206897_at	PAGE1	5.824
205801_s_at	RASGRP3	-6.387	218736_s_at	PALMD	5.835
212998_x_at	HLA-DQB1	-6.351	204734_at	KRT15	5.978
212063_at	CD44	-6.321	214039_s_at	LAPTM4B	6.004
220132_s_at	CLEC2D	-6.251	204533_at	CXCL10	6.053
221558_s_at	LEF1	-6.186	205098_at	CCR1	6.107
219073_s_at	OSBPL10	-6.165	221698_s_at	CLEC7A	6.122
219667_s_at	BANK1	-6.147	202551_s_at	CRIM1	6.174
204971_at	CSTA	-6.137	203666_at	CXCL12	6.201
219049_at	CSGALNACT1	-6.13	204066_s_at	AGAP1	6.228
201998_at	ST6GAL1	-5.816	44790_s_at	C13orf18	6.246
206150_at	CD27	-5.799	204105_s_at	NRCAM	6.259
211990_at	HLA-DPA1	-5.789	220415_at	TNNI3K	6.366
219926_at	POPDC3	-5.752	209803_s_at	PHLDA2	6.427
208683_at	CAPN2	-5.736	202350_s_at	MATN2	6.463
207734_at	LAX1	-5.673	203917_at	CXADR	6.479
220129_at	SOHLH2	-5.534	207979_s_at	CD8B	6.512
202252_at	RAB13	-5.527	200606_at	DSP	6.739
205671_s_at	HLA-DOB	-5.512	212599_at	AUTS2	6.791
200999_s_at	CKAP4	-5.435	202677_at	RASA1	6.806
213638_at	PHACTR1	-5.405	201445_at	CNN3	6.812
202746_at	ITM2A	-5.381	204151_x_at	AKR1C1	6.908
209942_x_at	MAGEA3	-5.351	204485_s_at	TOM1L1	6.909
203708_at	PDE4B	-5.323	219181_at	LIPG	6.931
209366_x_at	CYB5A	-5.277	212192_at	KCTD12	7.011
203895_at	PLCB4	-5.25	221210_s_at	NPL	7.063
213170_at	GPX7	-5.208	205549_at	PCP4	7.135
221297_at	GPRC5D	-5.158	209699_x_at	AKR1C2	7.169
201925_s_at	CD55	-5.155	201667_at	GJA1	7.485
221571_at	TRAF3	-5.155	210095_s_at	IGFBP3	7.581
219371_s_at	KLF2	-5.132	206336_at	CXCL6	7.688
205884_at	ITGA4	-5.09	205898_at	CX3CR1	7.769
201952_at	ALCAM	-5.018	212094_at	PEG10	7.778
206609_at	MAGEC1	-5.014	201427_s_at	SEPP1	8.534

Table 1: *(continued)*

U133a ID	Gene Symbol	Weight	U133a ID	Gene Symbol	Weight
203397_s_at	GALNT3	-4.979	209348_s_at	MAF	8.855
202947_s_at	GYPC	-4.966	209395_at	CHI3L1	9.056
205632_s_at	PIP5K1B	-4.953	209160_at	AKR1C3	9.105
210889_s_at	FCGR2B	-4.935	205336_at	PVALB	9.142
209619_at	CD74	-4.926	211719_x_at	FN1	9.776
