# Supplementary material for: Generation of a Predictive Melphalan Resistance Index by Drug Screen of B-Cell Cancer Cell Lines
Source: PLoS One. 2011 Apr 29;6(4):e19322. doi: 10.1371/journal.pone.0019322 (PMC3084810; doi:10.1371/journal.pone.0019322)
Supplement: Text S1 — Sweave Document. This document contains a supporting Sweave document with text, source code, and figures. (PDF) [file pone.0019322.s019.pdf]

Text S1: Sweave Document

Generation of a Predictive Melphalan Resistance Index by Drug Screen of B-Cell Cancer Cell Lines

Martin Boegsted, Johanne M. Holst, Kirsten Fogd, Steffen Falgreen,
Suzette Sørensen, Alexander Schmitz, Anne Bukh,
Hans E. Johnsen, Mette Nyegaard, Karen Dybkaer

April 6, 2011

Steffen Falgreen and Martin Boegsted

Contents

1 Data Aquisition and R settings **4**
1.1 Data Aqusition . . . 4
1.2 R Settings . . . 5
1.3 Various Parameter Settings . . . 6
1.4 Loading Functions . . . 6
1.5 R-session Information . . . 6

2 Loading the Retrospective Clinical Data **8**
2.1 Loading the *Arkansas* Data . . . 8
2.1.1 Reading the Metadata . . . 8
2.1.2 Creating Survival Objects . . . 8
2.1.3 Loading and Normalising the .CEL Files . . . 8
2.2 Loading the *Hummel* Data . . . 9
2.2.1 Reading the Metadata . . . 9
2.2.2 Creating the Survival Object . . . 10
2.2.3 Loading and Normalising the .CEL Files . . . 10

3 Establishment of the *NCI60* Gene Expression Data **11**

4 Establishment of the *NCI60* Dose Response Data **13**
4.1 The NCI60 Cell Line Metadata . . . 14
4.2 Converting .csv to .Rdata . . . 14

5 Establishment of the *NCI60* LDA Classifier **18**
5.1 Definition of the *NCI60* Resistance Classes . . . 18
5.2 Cross-Validation . . . 19
5.3 Establishing the LDA Classifier . . . 24

6 Testing the *NCI60* LDA Classifier on the Arkansas Data **30**
6.1 Predicting Melphalan Resistance Classes . . . 30
6.2 Association between the Resistance Classes and OS . . . 30
6.3 Association between the Resistance Classes and EFS . . . 31

7 Establishment of the *NCI60* SPLS Resistance Index **33**

7.1 Cross-Validation 33
7.2 Establishment of the Gene Expression Signature 39
7.3 Investigation of the chosen Gene Expression Profiles 40
7.4 Plots of Marginal Associations 41

8 Testing the *NCI60* SPLS Resistance Index on the Arkansas Data Set 43
8.1 Predicting the Resistance Index in the *Arkansas* data 43
8.2 Association between the Resistance Index and OS 43
8.2.1 Cox Proportional Hazards 44
8.2.2 Association between the Resistance Index and EFS 45

9 Establishment of the *BCell* Gene Expression Data 49
9.1 RMA Normalisation of the HG-U133 Plus 2.0 Arrays 49
9.2 Restriction to the Affymetrix HG-U133A Subset 51

10 Establishment of the *BCell* Dose Response Data 52
10.1 Calculation of the Percentage Growth 53
10.2 Calculation of the GI_{50} values 57
10.3 Subsampling with Replacement 59

11 Establishment of the *BCell* LDA Classifier 62
11.1 Definition of the *BCell* Resistance Classes 62
11.2 Cross-Validation 64
11.3 Establishing the LDA Classifier 69

12 Testing the *BCell* LDA Classifier on the Arkansas Data 76
12.1 Predicting the Melphalan Resistance Classes 76
12.2 Association between the Resistance Classes and OS 76
12.3 Association between the Resistance Classes and EFS 77

13 Establishment of the *BCell* SPLS Resistance Index 79
13.1 Cross-Validation 79
13.2 Establishment of the Gene Expression Signature 86
13.3 Investigation of the chosen Gene Expression Profile 86
13.4 Plots of Marginal Associations 88

14 Testing the *BCell* SPLS Resistance Index 93
14.1 Predicting the Melphalan Resistance in the *Arkansas* data 93
14.1.1 Association between the Resistance Index and OS 93
14.1.2 Association between the Resistance Index and EFS 96
14.2 Predicting Melphalan Resistance in the *Hummel* Data 99
14.2.1 Association between the Resistance Index and OS 99
14.3 Regularisation Paths for the BCell Panel 101

15 Simple Analysis 107

15.1 Predicting Melphalan Resistance in the *Arkansas* data 107
15.1.1 Association between the Melphalan Resistance Index and OS 108
15.1.2 Association between the Melphalan Resistance Index and EFS 110

16 Auxiliary Functions **115**
16.1 Dose Response Functions 115
16.2 Model Building Functions 127
16.3 Functions used for the SPLS Analysis 127
16.4 Functions used in the Simple Analysis 136
16.5 Annotation and Plotting Tools 136

1 Data Aquisition and R settings

1.1 Data Aqusition

The *NCI60* Cancer Cell Line Panel

The *NCI60* panel consists of 59 cancer cell lines. Gene expression data, dose response and metadata are publicly available.

Affymetrix GeneChip Human Genome U133A (HG-U133A) arrays with gene expression data are available as .CEL files via

- `http://www.ncbi.nlm.nih.gov/geo/`

under GEO accession number GSE5720.

The DTP human tumor cell line drug screen data (August 2008 release) were downloaded from the website

- `http://dtp.nci.nih.gov/docs/cancer/cancer_data.html`

The metadata were downloaded May 22, 2009, from

- `http://discover.nci.nih.gov/cellminer/celllineinfo.do`

The *BCell* Cancer Cell Line Panel

The *BCell* panel consists of 18 cancer cell lines all originating from patients suffering from a B-cell malignancy. Affymetrix GeneChip Human Genome U133 Plus 2.0 arrays (HG-U133 Plus 2.0) have been used to obtain the gene expression data of the cell lines.
The gene expression data have been deposited at

- `http://www.ncbi.nlm.nih.gov/geo/`

under GEO accession number GSE22759.

Retrospective Clinical Data Sets

The *Arkansas* Data

The *Arkansas* data consist of data from 565 patients with multiple myeloma (MM). The data include gene expressions from a HG-U133Plus 2.0 array platform and information about overall survival (OS) and event free survival (EFS). The metadata and .CEL files are available at

- `http://www.ncbi.nlm.nih.gov/geo/`

under GEO accession number GSE24080.

The *Hummel* Data

The *Hummel* data consist of data from patients suffering from diffuse large B-cell lymphoma (DLBCL). The data include gene expressions obtained through the HG-U133A microarray platform and OS. The metadata and .CEL files are available at

- `http://www.ncbi.nlm.nih.gov/geo/`

under accession number GSE4475.

1.2 R Settings

```
> options(width       = 70)
> options(continue    = "  ")
> options(digits      = 22)
> windowsFonts(Times = windowsFont("TT Times New Roman"))
> pdf.options(family = "Times")
> par(family          = "Times")
```

The libraries which are required for the analysis are loaded below

```
> require(affy)
> require(annaffy)
> require(annotate)
> require(annotationTools)
> require(arrayQualityMetrics)
> require(Biobase)
> require(caTools)
> require(Design)
> require(foreign)
> require(genefilter)
> require(geneplotter)
> require(gdata)
> require(glmnet)
> require(gplots)
> require(hgu133a.db)
> require(hgu133a2.db)
> require(hgu133aprobe)
> require(hgu133plus2.db)
> require(limma)
> require(nlme)
> require(outliers)
> require(pls)
> require(prada)
> require(quantreg)
```

```
> require(RColorBrewer)
> require(sda)
> require(spls)
> require(statmod)
> require(survival)
> require(TTR)
> require(vsn)
> require(XML)
> require(xtable)
```

1.3 Various Parameter Settings

In the following code chunk the color scheme for the plots is determined, the cut.off values used for the KM plots are set, and the working catalogue is specified.

```
> our.colscheme        <- c("black", "darkgrey", "red")
> names(our.colscheme) <- c("Sensitive", "Intermediate", "Resistant")
> cut.points           <- c(0.25, 0.75)
> setwd(working.directory)
```

1.4 Loading Functions

Auxilary functions used throughout are printed out in Section 16.

1.5 R-session Information

Information regarding the R-session is printed below

```
> toLatex(sessionInfo())
```

- R version 2.12.1 (2010-12-16), `x86_64-pc-mingw32`
- Locale: `LC_COLLATE=Danish_Denmark.1252`, `LC_CTYPE=Danish_Denmark.1252`, `LC_MONETARY=Danish_Denmark.1252`, `LC_NUMERIC=C`, `LC_TIME=Danish_Denmark.1252`
- Base packages: base, datasets, graphics, grDevices, grid, methods, splines, stats, utils
- Other packages: affy 1.28.0, affyPLM 1.26.0, annaffy 1.22.0, annotate 1.28.0, AnnotationDbi 1.12.0, annotationTools 1.22.0, arrayQualityMetrics 3.2.4, Biobase 2.10.0, bitops 1.0-4.1, cacheSweave 0.4-5, caTools 1.11, corpcor 1.5.7, DBI 0.2-5, Design 2.3-0, entropy 1.1.5, fdrtool 1.2.6, filehash 2.1-1, foreign 0.8-41, gcrma 2.22.0, gdata 2.8.1, genefilter 1.32.0, geneplotter 1.28.0, glmnet 1.5.1, GO.db 2.4.5, gplots 2.8.0, gtools 2.6.2, hgu133a.db 2.4.5, hgu133a2.db 2.4.5, hgu133aprobe 2.7.0, hgu133plus2.db 2.4.5, Hmisc 3.8-3, KEGG.db 2.4.5,

lattice 0.19-13, limma 3.6.9, MASS 7.3-9, Matrix 0.999375-46, mvtnorm 0.9-95, nlme 3.1-97, nnet 7.3-1, org.Hs.eg.db 2.4.6, outliers 0.13-3, pcaPP 1.8-3, pls 2.1-0, prada 1.26.0, preprocessCore 1.12.0, quantreg 4.53, RColorBrewer 1.0-2, robustbase 0.5-0-1, rrcov 1.1-00, RSQLite 0.9-4, sda 1.1.0, SparseM 0.86, spls 2.1-0, stashR 0.3-3, statmod 1.4.8, survival 2.36-2, TTR 0.20-2, vsn 3.18.0, XML 3.2-0.2, xtable 1.5-6, xts 0.7-5, zoo 1.6-4

- Loaded via a namespace (and not attached): affyio 1.18.0, beadarray 2.0.2, Biostrings 2.18.2, cluster 1.13.2, digest 0.4.2, hwriter 1.3, IRanges 1.8.8, latticeExtra 0.6-14, marray 1.28.0, simpleaffy 2.26.1, stats4 2.12.1, SVGAnnotation 0.7-2, tools 2.12.1

2 Loading the Retrospective Clinical Data

2.1 Loading the *Arkansas* Data

A directory which includes the *Arkansas* data and a directory to store the generated data are created.

```
> Arkansas.ext.dir <- file.path(getwd(), "External data/Arkansas")
> Arkansas.gen.dir <- file.path(getwd(), "Generated data/Arkansas")
```

The directory storing generated data is created.

```
> dir.create(path = file.path(Arkansas.gen.dir),
             showWarnings = FALSE, recursive = TRUE, mode = "0777")
```

2.1.1 Reading the Metadata

The metadata was transformed manually from XLS to the semicolon-separated file `Arkansasmetadata.csv`.

```
> metadataArkansas <-
      read.csv2(file.path(Arkansas.ext.dir,
                          "Metadata", "Arkansasmetadata.csv"))
> n.Arkansas.all <- nrow(metadataArkansas)
```

2.1.2 Creating Survival Objects

The OS and EFS survival objects are created using the `Surv` function from the `survival` package.

```
> metadataArkansas$OS <-
      Surv(as.numeric(metadataArkansas$OS..months.),
           metadataArkansas$OS.censor == 1)

> metadataArkansas$EFS <-
      Surv(as.numeric(metadataArkansas$EFS.months.),
           metadataArkansas$EFS.censor == 1)
```

2.1.3 Loading and Normalising the .CEL Files

The .CEL files are loaded into R. The metadata are restricted to patients with an associated .CEL file.

```
> row.names(metadataArkansas) <- metadataArkansas$CELfilename
> files <- dir(file.path(Arkansas.ext.dir, "Celfiles"))
> metadataArkansas <- metadataArkansas[substring(files, 11, 100), ]
> n.Arkansas <- nrow(metadataArkansas)
> row.names(metadataArkansas) <- metadataArkansas$PATID
```

This resulted in the removal of 6 patients. Finally, the .CEL files are read and pre-processed using the RMA approach.

```
> GEPArkansas.file <- file.path(Arkansas.gen.dir, "GEPArkansas.Rdata")
> if(file.exists(GEPArkansas.file)){
      load(GEPArkansas.file)
  }else{
      phenoData <- new("AnnotatedDataFrame", data = metadataArkansas)
      cel.files <- file.path(Arkansas.ext.dir, "Celfiles", files)

      GEPArkansas <- just.rma(filenames = cel.files,
                              phenoData = phenoData)

      # The data is only saved if the rownames in the phenoData match
      # the colnames of the gene expression data.
      if(all(rownames(pData(GEPArkansas)) ==
             colnames(exprs(GEPArkansas)))){
          save(GEPArkansas, file=GEPArkansas.file)
      }
  }
```

2.2 Loading the *Hummel* Data

Directories which include the *Hummel* and the generated data are created.

```
> Hummel.ext.dir <- file.path(getwd(), "External data/Hummel")
> Hummel.gen.dir <- file.path(getwd(), "Generated data/Hummel")

> dir.create(path = file.path(Hummel.gen.dir),
             showWarnings = FALSE, recursive = TRUE, mode = "0777")
```

2.2.1 Reading the Metadata

```
> metadataHummel <-
      read.csv(file.path(Hummel.ext.dir,
                         "Metadata", "GSE4475_Clinical.csv"))
```

Attention is restricted to patients for whom the follow up time is observed and is greater than 0 and information regarding the given chemotherapy is registered.

```
> n.na.FU        <-
      dim(metadataHummel[is.na(metadataHummel$FollowupTime..months), ])[1]
> metadataHummel <-
      metadataHummel[!is.na(metadataHummel$FollowupTime..months), ]
> n.zero         <-
```

```
    dim(metadataHummel[metadataHummel$FollowupTime..months == 0, ])[1]
> metadataHummel <- metadataHummel[metadataHummel$FollowupTime..months > 0, ]
> n.na.chemo    <-
    dim(metadataHummel[is.na(metadataHummel$Chemotherapy), ])[1]
> metadataHummel <- metadataHummel[!is.na(metadataHummel$Chemotherapy), ]
```

Only patients who have received ”CHOP-like” chemotherapy are used.

```
> chosen          <- as.character(metadataHummel$Chemotherapy) == "CHOP-like"
> n.not.CHOP     <- dim(metadataHummel[!chosen,])[1]
> metadataHummel <- metadataHummel[chosen,]
```

This results in a dataset with 87 patients. Table 2.1 shows the number of patients removed in each step.

Reason	Patients excluded
No follow up information	62
Follow up equal to 0	4
No chemotherapy information	16
Chemotherapy other than CHOP	52

Table 2.1: Number of excluded patients in each step of the *Hummel* data set.

2.2.2 Creating the Survival Object

```
> metadataHummel$OS <-
    Surv(as.numeric(metadataHummel$FollowupTime..months),
         metadataHummel$Survival.Status == "dead")
```

2.2.3 Loading and Normalising the .CEL Files

```
> file <- file.path("Generated data", "Hummel", "HummelHGU133A.Rdata")
> if(file.exists(file)){
    load(file)
  }else{
    GEPHummel<-
      justRMA(filenames = file.path("External data/Hummel/Celfiles",
              paste(metadataHummel$GEO.ID,".CEL", sep = "")))

    dir.create(path=paste("Generated data/Hummel/", sep = ""),
              showWarnings = FALSE, recursive = TRUE, mode = "0777")

    save(GEPHummel, file = file)
  }
```

3 Establishment of the *NCI60* Gene Expression Data

The directories which are used with the *NCI60* cell line panel are defined

```
> NCI60.ext.dir <- file.path(getwd(), "External data",  "NCI60")
> NCI60.gen.dir <- file.path(getwd(), "Generated data", "NCI60")
```

and created

```
> dir.create(NCI60.gen.dir,
             showWarnings = FALSE, recursive = TRUE, mode = "0777")
```

The NCI60 .CEL files are read into R in the following code chunk. For pre-processing of the arrays the RMA approach is used.

```
> GEPNCI60.file <- file.path(NCI60.gen.dir, "GEPNCI60.RMA.Rdata")
> if(file.exists(GEPNCI60.file)){
      load(GEPNCI60.file)
  }else{
      ## The metadata for the .cel files are stored in the file

      NCI60.cel.names <-
          read.table(file.path(NCI60.ext.dir, "Celfiles", "u133a.fnames.txt"),
                     sep         = "",
                     header      = TRUE,
                     check.names = TRUE,
                     row.names   = 1,
                     col.names   = c("cell.line.name", "array.type"))

      ## The NCI60 data includes both HGU133A and HGU133B
      ## arrays. However, only the HGU133A arrays are used
      ## in the analysis

      NCI60.cel.names <-
          NCI60.cel.names[grep("hg133a", NCI60.cel.names$array.type),]

      ## The cell line IGROV1 is dublicated and data from
      ## experiment a21 are chosen

      NCI60.cel.names <-
          NCI60.cel.names[NCI60.cel.names$cell.line.name != "IGROV1" |
                          NCI60.cel.names$array.type     != "hg133a31", ]
```

```
    ## Some of the cell line names are extended with /ATCC and
    ## the following code removes the extension

    NCI60.cel.names$cell.line.name <-
        gsub("/ATCC", "", as.character(NCI60.cel.names$cell.line.name))

    ## Finally, the .CEL files are imported and RMA normalised

    GEPNCI60 <-
        justRMA(filenames    = paste(rownames(NCI60.cel.names),
                ".CEL",  sep = ""),
                celfile.path = file.path(NCI60.ext.dir,"Celfiles"))

    ## The sample names of the exprs object are
    ## set equal to sample names from the metadata

    sampleNames(GEPNCI60) <-
        as.character(NCI60.cel.names$cell.line.name)
    sampleNames(GEPNCI60) <- gsub("-", "\\.", sampleNames(GEPNCI60))
    save(GEPNCI60, file = GEPNCI60.file)
}

> n.NCI60 <- dim(GEPNCI60)[2]
> p.NCI60 <- dim(GEPNCI60)[1]
> GEPNCI60133a <- GEPNCI60
```

4 Establishment of the *NCI60* Dose Response Data

Parts of the code used in the present chapter have been developed by Kevin Coombes and Keith Baggerley and can be downloaded from the website

- http://bioinformatics.mdanderson.org/Supplements/ReproRsch-Chemo/

The two output catalogues are created by the code chunk below.

```
> dir.create(path=figure.output,
             showWarnings = FALSE, recursive = TRUE, mode = "0777")
> dir.create(path=table.output,
             showWarnings = FALSE, recursive = TRUE, mode = "0777")
```

The DTP human tumor cell line drug screen data (August 2008 release) were downloaded from the website

- http://dtp.nci.nih.gov/docs/cancer/cancer_data.html

This contained the three files:

1. cancer60gi50.lis
2. cancer60lc50.lis
3. cancer60tgi.lis.

Dose response data regarding melphalan induced GI_{50} values are extracted. The data are stored in the directory below.

```
> setwd("External data/NCI60/Doseresponse/Processed/")
```

A Perl script which extracts the melphalan data from the GI_{50} data is invoked.

```
> file       <- paste("CANCER60", "GI50", ".LIS", sep="")
> sourceFile <- paste("../Raw/", file, sep = "")
> targetFile <- paste(file, "MMproject", "csv", sep = ".")
> command    <- paste("perl strip4MMproject.pl", sourceFile, targetFile)
> if(file.exists(targetFile)){
      system(command)
  }
> setwd("../../../../")
```

4.1 The NCI60 Cell Line Metadata

The Cell Miner metadata were downloaded May 22, 2009, from

- `http://discover.nci.nih.gov/cellminer/celllineinfo.do`

into the excel document `cellLine_metadata_783789569.xls`. The file was transformed manually from XLS to the semicolon-separated file *NCI60metadata.csv*.

```
> metadataNCI60 <-
      read.csv2(file.path(NCI60.ext.dir, "Metadata", "NCI60metadata.csv"))
```

Change the problematic names.

```
> metadataNCI60$Cell.Name <- gsub("_", ".", metadataNCI60$Cell.Name)
```

Save dataset in the data folder.

```
> save(metadataNCI60,
       file = file.path(NCI60.gen.dir, "metadataNCI60.Rdata"))
```

4.2 Converting .csv to .Rdata

```
> nsc          <- "8806"
> names(nsc) <- "Melphalan"
```

The function getNSC is able to read the data from the comma-separated source files.

```
> nci60Dir <- file.path(NCI60.ext.dir, "Doseresponse",  "Processed")
> gi50     <- getNSC("cancer60gi50.lis.MMproject.csv", "NLOGGI50", nci60Dir)
```

Drug response data of the cell lines which are present in the NCI60 metadata are chosen.

The cell line name stored in the metadata includes an abbreviation describing which tissue group it originates from. This information is not included in the dose response data. The code chunk below removes the information ensuring that the two data sets are comparable.

```
> xx1 <- metadataNCI60$Cell.Name
> xx2 <- unlist(strsplit(xx1,":"))[2 * (0:(length(xx1) - 1)) + 2]
> xx  <- sort(xx2)
```

The rownames of the dose response data are stored

```
> yy <- rownames(gi50)
```

and corrected.

```
> yy[yy == "HL-60(TB)"]        <- "HL-60"
> yy[yy == "RXF 393"]          <- "RXF.393"
> yy[yy == "T-47D"]            <- "T47D"
> yy[yy == "NCI/ADR-RES"]      <- "NCI.ADR.RES"
> yy[yy == "A549/ATCC"]        <- "A549"
> yy[yy == "MDA-MB-231/ATCC"] <- "MDA.MB.231"
> yy <- gsub(" ", "", gsub(c("-"), c("."), yy))
> novi <- yy %in% xx
```

A matrix consisting of the GI_{50} valuse is established.

```
> GI50           <- gi50[novi, ]
> rownames(GI50) <- xx1[order(xx2)]
> rownames(GI50) <- xx2[order(xx2)]
>
```

The dose response data regarding melphalan are stored in a .Rdata object.

```
> save(nsc, GI50, file = file.path(NCI60.gen.dir, "nscdata.Rdata"))
```

The resistance index which is used in the analysis is established

```
> NCI60Resistanceindex <- - 1 * GI50[, 1]
```

When the NCI60 resistance index is used to establish a gene expression signature the gene expxressions stored in the expression set `GEPNCI60` are used as input variables. It is therefore necessary to align these objects.

```
> NCI60.names <- sampleNames(GEPNCI60)
> excluded.cellline    <- setdiff(names(NCI60Resistanceindex), NCI60.names)
> NCI60Resistanceindex <-  cbind(NCI60Resistanceindex[NCI60.names])
> save(NCI60Resistanceindex,
       file = file.path(NCI60.gen.dir, "NCI60Resistanceindex.Rdata"))
```

The cell line names are stored in a vector for later use.

```
> NCI60.names.sorted <- names(NCI60Resistanceindex[order(NCI60Resistanceindex),])
```

The resistance index which is used in the analysis is established and plotted with the following code chunk.

```
> mel  <- NCI60Resistanceindex[order(NCI60Resistanceindex),]
> mean <- mean(mel)
> xx   <- mel - mean
> pdf(paste(figure.output,"/BarplotNCI60MEL.pdf", sep = ""),
      width = 4, height = 6)
> barplot2(xx, names.arg=names(mel),
           horiz     = TRUE, las = 2, offset = mean,
```

```
          plot.grid = TRUE, grid.inc = 10,
          space     = 0.4,
          col       = "black",
          cex.names = 0.4,
          cex.main  = 1,
          cex.axis  = 0.6,
          xlab      = "Log base 10 of the molar concentration",
          main      = "The NCI60 Melphalan Resistance Index"
          )
> dev.off()
```

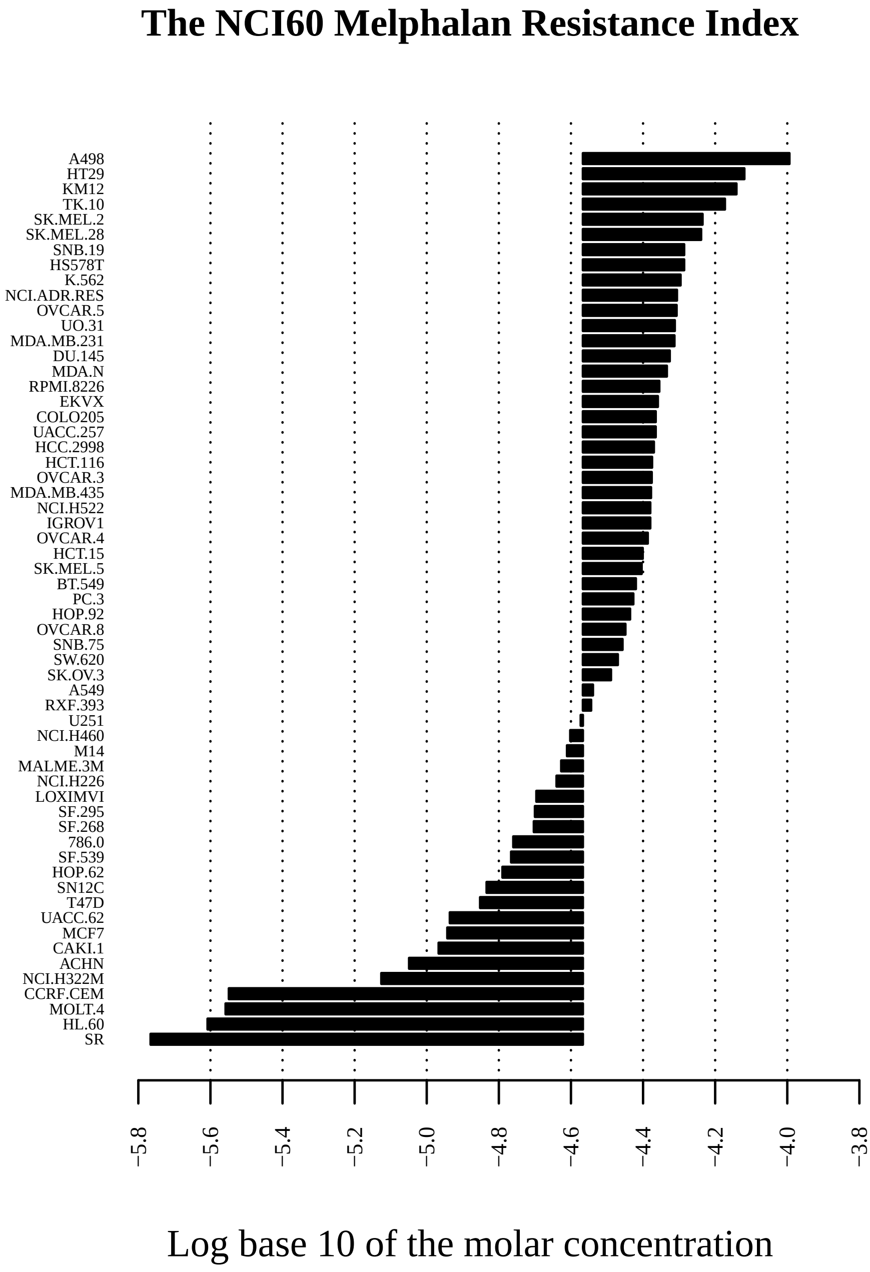

Figure 4.1: Barplot of the GI_{50}-value for melphalan treatment of the 59 cell lines in the NCI60 panel.

Finally, a clean up is performed.

```
> rm(novi, gi50, xx, xx1, xx2, yy)
```

5 Establishment of the *NCI60* LDA Classifier

The seed is set to ensure repeatability of the results.

```
> set.seed(1000)
```

The LDA analysis includes all gene expressions which are expressed significantly different between the sensitive, intermediate and resistant cell lines at a significance level of 0.05.

```
> pval <- 0.05
```

5.1 Definition of the *NCI60* Resistance Classes

The number of sensitive, intermediate and resistant cell lines are defined.

```
> sens <- 14
> resi <- 14
> inte <- n.NCI60 - sens - resi
> class.list <-  c(rep("Sensitive",    sens),
                   rep("Intermediate", inte),
                   rep("Resistant",    resi))
```

A data frame is constructed containing the GI_{50} values and the class of each cell line. The data frame is shown in Table 5.1

```
> sort.NCI60.class <-
      data.frame(NCI60Resistanceindex = sort(NCI60Resistanceindex),
                 class = class.list)
> row.names(sort.NCI60.class) <- NCI60.names.sorted
```

The data set is sorted according to the NCI60 melphalan resistance index.

```
> NCI60.class       <- sort.NCI60.class[NCI60.names,]
> NCI60.class$class <- as.character(NCI60.class$class)
```

The following code chunk constructs a box plot of the GI_{50} values grouped into resistant, intermediate and sensitive cell lines. The plot is shown in Figure 5.1.

```
> pdf(file.path(figure.output, "NCI60resvssensBoxplot.pdf"))
> sort.NCI60.class$class <- relevel(sort.NCI60.class$class,
                                    names(our.colscheme)[1])
> boxplot(sort.NCI60.class$NCI60Resistanceindex ~
          sort.NCI60.class$class,
          border = our.colscheme)
> dev.off()
```

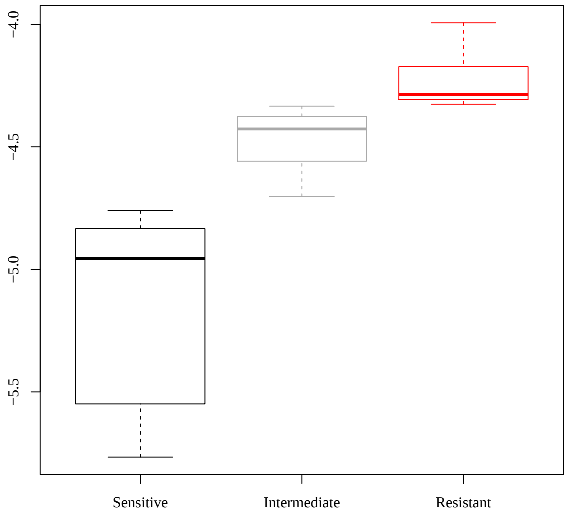

Figure 5.1: Box plot of the GI_{50} values grouped into resistant, intermediate and sensitive cell lines.

5.2 Cross-Validation

In order to choose optimal parameters for the LDA analysis cross-validation (CV) is used. In the code chunk below the cutoff value for the unspecific filtering is likewise determined through CV. In the method the parameters of the LDA analysis are determined through CV for a wide range of values for the parameter `var.cutoff` in the function `nsFilter`. The combination of parameters in LDA and the `var.cutoff` which results in the maximum accuracy is chosen for further analysis. We want to investigate whether the function `nsFilter` chooses gene expressions which perform better than random noise. The analysis is performed as described above, however, instead of selecting the gene expression profiles through `nsFilter` they are chosen at random.

The various fractions to be filtered out are determined

```
> cut.offs <- seq(0.01, 0.98, by = 0.01)
```

A data frame containing the CV accuracy for LDA based on the gene expression profiles obtained through nsFilter and random selection is defined.

```
> NCI60LDAcrossval.res <- data.frame(cut.offs = cut.offs,
                                     accuracy = 0,
                                     acc.rand = 0)
```

```
> NCI60LDAcrossval.res.file <-
      file.path(NCI60.gen.dir, "NCI60LDAcrossval.res.Rdata")
```

```
> if(file.exists(NCI60LDAcrossval.res.file)){
      load(NCI60LDAcrossval.res.file)
  }else{
      for(cut.off in cut.offs) {
          cell.res <-
              data.frame(nsFilter = rep(0, n.NCI60,
                         random   = rep(0, n.NCI60)))

          ## nsFilter expressions

          NCI60.filtered <- nsFilter(GEPNCI60[, rownames(NCI60.class)],
                                     var.cutoff = cut.off)$eset

          exprs(NCI60.filtered) <- t(scale(t(exprs(NCI60.filtered))))

          ## Random expressions

          p.NCI60.filt.LDA <- length(featureNames(NCI60.filtered))
          rand.probes <- sample(p.NCI60, p.NCI60.filt.LDA)

          NCI60.random <- GEPNCI60[rand.probes, rownames(NCI60.class)]

          exprs(NCI60.random) <- t(scale(t(exprs(NCI60.random))))

          ## Analysis with random expressions

          for(cell in 1:n.NCI60) {
              cat("cell line =", cell)
              ## Leave-one-out from arrays

              looNCI60.filtered <- NCI60.random[, - cell]

              ## Leave-one-out from factor levels

              factor.NCI60 <- factor(as.character(NCI60.class$class[ - cell]))

              design.NCI60 <- model.matrix( ~ 0 + factor.NCI60)

              colnames(design.NCI60) <- levels(factor.NCI60)

              contrast.matrixNCI60 <-
                  makeContrasts(Resistant    - Sensitive,
                                Intermediate - Sensitive,
                                levels       = design.NCI60)
```

```
    ## Linear model fit

    fitNCI60 <- lmFit(looNCI60.filtered,
                      design = design.NCI60)

    fit2NCI60 <- contrasts.fit(fitNCI60, contrast.matrixNCI60)
    fit2NCI60 <- eBayes(fit2NCI60)

    NCI60lmFit <- topTable(fit2NCI60,
                           coef = 1:(length(
                                     unique(NCI60.class$class)) - 1),
                           adjust = "BH", number = Inf)

    ## In the case where more than 400 genes are significantly differentially
    ## expressed at 0.05 level only the top 400 are used

    probesets.NCI60 <- NCI60lmFit$ID[NCI60lmFit$P.Val <= pval]
    probesets.NCI60 <- probesets.NCI60[1:min(length(probesets.NCI60), 400)]
    NCI601.filtered <-
        looNCI60.filtered[probesets.NCI60,]

    fitLDANCI60 <-
        sda(t(exprs(NCI601.filtered)), NCI60.class$class[ - cell])

    ## Internal prediction of the cell lines

    predi <-
        predict(fitLDANCI60,
                t(exprs(NCI60.random[probesets.NCI60, cell])))

    ## Correctly classified

    cell.res$random[cell] <-
        as.numeric(predi$class == NCI60.class$class[cell])
}

cat("nsFilter analysis")
for(cell in 1:n.NCI60) {
    cat(cell)
    ## Leave-one-out from arrays

    looNCI60.filtered <- NCI60.filtered[, -cell]

    ## Leave-one-out from factor levels
```

```
factor.NCI60 <- factor(as.character(NCI60.class$class[ - cell]))

design.NCI60 <- model.matrix(~ 0 + factor.NCI60)

colnames(design.NCI60) <- levels(factor.NCI60)

contrast.matrixNCI60  <-
    makeContrasts(Resistant    - Sensitive,
                  Intermediate - Sensitive,
                  levels       = design.NCI60)
## Linear model fit

fitNCI60 <- lmFit(looNCI60.filtered,
                  design = design.NCI60)

fit2NCI60 <- contrasts.fit(fitNCI60, contrast.matrixNCI60)
fit2NCI60 <- eBayes(fit2NCI60)

NCI60lmFit <- topTable(fit2NCI60,
                       coef = 1:(length(
                                 unique(NCI60.class$class)) - 1),
                       adjust = "BH", number = Inf)

## In the case where more than 400 genes are significantly differentially
## expressed at 0.05 level only the top 400 are used

probesets.NCI60 <- NCI60lmFit$ID[NCI60lmFit$P.Val <= pval]
probesets.NCI60 <- probesets.NCI60[1:min(length(probesets.NCI60), 400)]

NCI601.filtered <-
    looNCI60.filtered[probesets.NCI60,]

fitLDANCI60 <-
    sda(t(exprs(NCI601.filtered)), NCI60.class$class[ - cell])

## Internal prediction of the cell lines

predi <-
    predict(fitLDANCI60,
            t(exprs(NCI60.filtered[probesets.NCI60, cell])))

## Correctly classified
```

```
            cell.res$nsFilter[cell] <-
                as.numeric(predi$class == NCI60.class$class[cell])
        }
        cat("var.cutoff =", cut.off)
        cat("mean accuracy =",  mean(cell.res$nsFilter))
        NCI60LDAcrossval.res[NCI60LDAcrossval.res$cut.offs ==
                             cut.off, ]$accuracy <- mean(cell.res$nsFilter)
        NCI60LDAcrossval.res[NCI60LDAcrossval.res$cut.offs ==
                             cut.off, ]$acc.rand <- mean(cell.res$rand)
    }
    save(NCI60LDAcrossval.res, file = NCI60LDAcrossval.res.file)
}
```

The combination of parameters resulting in the maximum accuracy is stored in the object accuracy.max,

```
> accuracy.max <-
    NCI60LDAcrossval.res[NCI60LDAcrossval.res$accuracy ==
                         max(NCI60LDAcrossval.res$accuracy), ]
```

and plotted in Figure 5.2.

```
> pdf(file.path(figure.output,"NCI60LDAcomparison.pdf"))
> plot(NCI60LDAcrossval.res$cut.offs,
       NCI60LDAcrossval.res$accuracy,
       ylim = c(min(NCI60LDAcrossval.res$accuracy,
                    NCI60LDAcrossval.res$acc.rand),
                max(NCI60LDAcrossval.res$accuracy,
                    NCI60LDAcrossval.res$acc.rand)),
       ylab = "Predicted Accuracy",
       xlab = "Fraction filtered out",
       type = "n",
       main = "NCI60 Predicted Accuracy")
> lines(NCI60LDAcrossval.res$cut.offs,
        NCI60LDAcrossval.res$accuracy,
        lty = 1, col = "black")
> lines(NCI60LDAcrossval.res$cut.offs,
        NCI60LDAcrossval.res$acc.rand,
        lty = 2, col = "black")
> legend("bottomleft", lty = c(1,2,1), lwd = c(1,1,2),
         legend = c("nsFiltered probes",
                    "Random probes"),
         col = "black", bty = "n")
> dev.off()
```

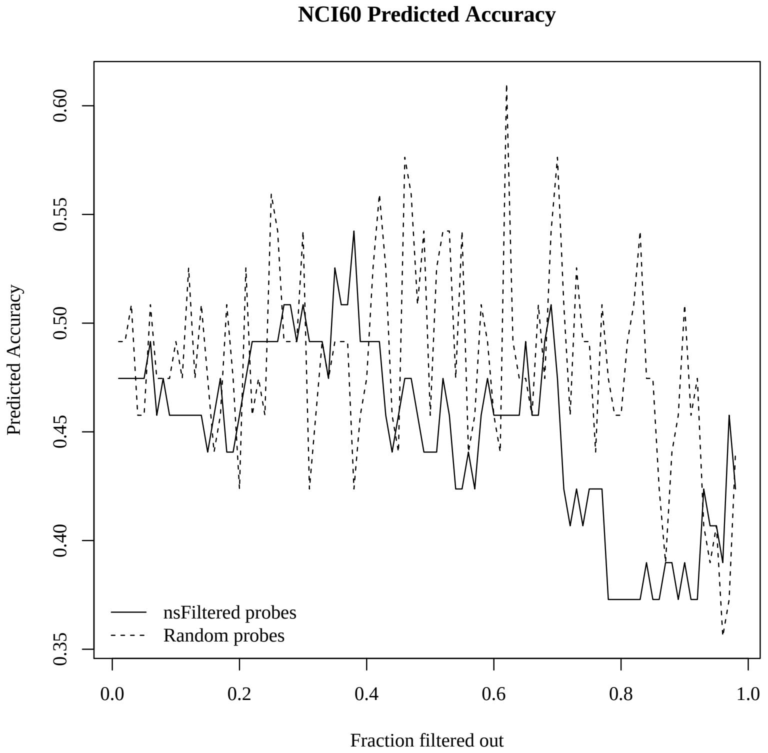

Figure 5.2: CV accuracy for the LDA analysis at various values of the parameter var.cutoff in nsFilter. The maximum accuracy 0.542 is achieved when var.cutoffs is equal to 0.38.

5.3 Establishing the LDA Classifier

The value of the parameter `var.cutoff` resulting in the maximum accuracy is used on the entire data set in the function `nsFilter`

```
> cut.off <- accuracy.max$cut.offs[length(accuracy.max$cut.offs)]
> NCI60.filtered <- nsFilter(GEPNCI60[, rownames(NCI60.class)],
                             var.cutoff = cut.off)$eset
```

resulting in an expression set with 59 samples and 7861 features. The gene expressions are scaled to have unit variance and zero mean.

```
> exprs(NCI60.filtered) <- t(scale(t(exprs(NCI60.filtered))))
```

The design matrix used in `lmFit` to extract the gene expression profiles which are significantly differentially expressed between the three groups is established.

```
> factor.NCI60 <- factor(NCI60.class$class)
> design.NCI60 <- model.matrix(~ 0 + factor.NCI60)
> colnames(design.NCI60) <- levels(factor.NCI60)
```

A matrix defining the set of contrasts is established through the function `makeContrasts` from the `limma` package.

```
> contrast.matrixNCI60 <-
      makeContrasts(Resistant    - Sensitive,
                    Intermediate - Sensitive,
                    levels       = design.NCI60)
```

Finally, a linear model is fitted.

```
> fitNCI60 <- lmFit(NCI60.filtered,
                    design = design.NCI60)
```

The coefficients and standard errors are estimated according to the constructed set of contrasts.

```
> fit2NCI60 <- contrasts.fit(fitNCI60, contrast.matrixNCI60)
```

Using the functions `eBayes` and `topTable` the gene expression profiles are ranked according to the FDR for differential expressions between the three categories of resistance to melphalan.

```
> fit2NCI60  <- eBayes(fit2NCI60)
> NCI60lmFit <- topTable(fit2NCI60,
                         coef   = 1:(length(unique(NCI60.class$class)) - 1),
                         adjust = "BH",
                         number = Inf)
```

The gene expressions which are expressed significantly different with a significance level of 0.05 are selected for further analysis. If the number exceeds 400 only the top 400 are used.

```
> probesets.NCI60 <- NCI60lmFit$ID[NCI60lmFit$P.Val <= pval]
> probesets.NCI60 <- probesets.NCI60[1:min(length(probesets.NCI60), 400)]
> p.NCI60.LDA <- length(probesets.NCI60)
```

This results in a 400 gene expression profile. The gene symbols are looked up and stored together with the topTable result.

```
> NCI60LDAgenes <-
      as.vector(unlist(lookUp(probesets.NCI60, "hgu133plus2", "SYMBOL")))
> probesets.LDA.NCI60            <- matrix(NA, nrow = p.NCI60.LDA, ncol = 5)
> row.names(probesets.LDA.NCI60) <- probesets.NCI60
> probesets.LDA.NCI60[,2:5]      <-
      signif(as.matrix(NCI60lmFit[1:p.NCI60.LDA,4:7]), 4)
> colnames(probesets.LDA.NCI60)  <- c("Symbol", colnames(NCI60lmFit[,4:7]))
> probesets.LDA.NCI60[,1]        <- NCI60LDAgenes
```

The first 40 genes are shown in Table 5.2. Finally, the LDA classifier is built using the function `sda` from the package `sda`.

```
> fitLDANCI60 <-
    sda(t(exprs(NCI60.filtered[probesets.NCI60,])), NCI60.class$class)
```

Table 5.1: A summary of the resistance levels and defined resistance classes for each cell line.

Cell line	NCI60Resistanceindex	class
SR	–5.77	Sensitive
HL.60	–5.61	Sensitive
MOLT.4	–5.56	Sensitive
CCRF.CEM	–5.55	Sensitive
NCI.H322M	–5.13	Sensitive
ACHN	–5.05	Sensitive
CAKI.1	–4.97	Sensitive
MCF7	–4.94	Sensitive
UACC.62	–4.94	Sensitive
T47D	–4.85	Sensitive
SN12C	–4.83	Sensitive
HOP.62	–4.79	Sensitive
SF.539	–4.77	Sensitive
786.0	–4.76	Sensitive
SF.268	–4.70	Intermediate
SF.295	–4.70	Intermediate
LOXIMVI	–4.70	Intermediate
NCI.H226	–4.64	Intermediate
MALME.3M	–4.63	Intermediate
M14	–4.61	Intermediate
NCI.H460	–4.60	Intermediate
U251	–4.57	Intermediate
RXF.393	–4.54	Intermediate
A549	–4.54	Intermediate
SK.OV.3	–4.49	Intermediate
SW.620	–4.47	Intermediate
SNB.75	–4.46	Intermediate
OVCAR.8	–4.45	Intermediate
HOP.92	–4.44	Intermediate
PC.3	–4.43	Intermediate
BT.549	–4.42	Intermediate
SK.MEL.5	–4.40	Intermediate
HCT.15	–4.40	Intermediate
OVCAR.4	–4.39	Intermediate
IGROV1	–4.38	Intermediate
NCI.H522	–4.38	Intermediate
MDA.MB.435	–4.38	Intermediate
OVCAR.3	–4.38	Intermediate
HCT.116	–4.38	Intermediate
HCC.2998	–4.37	Intermediate

Table 5.1: *(continued)*

Cell line	NCI60Resistanceindex	class
UACC.257	–4.37	Intermediate
COLO205	–4.37	Intermediate
EKVX	–4.36	Intermediate
RPMI.8226	–4.36	Intermediate
MDA.N	–4.33	Intermediate
DU.145	–4.33	Resistant
MDA.MB.231	–4.31	Resistant
UO.31	–4.31	Resistant
OVCAR.5	–4.31	Resistant
NCI.ADR.RES	–4.31	Resistant
K.562	–4.30	Resistant
HS578T	–4.29	Resistant
SNB.19	–4.29	Resistant
SK.MEL.28	–4.24	Resistant
SK.MEL.2	–4.24	Resistant
TK.10	–4.17	Resistant
KM12	–4.14	Resistant
HT29	–4.12	Resistant
A498	–3.99	Resistant

Table 5.2: A toptable for the first 40 gene expressions used in the LDA classification.

Probesets	Symbol	F	P.Value	adj.P.Val
214736_s_at	ADD1	7.888	0.000375	0.8467
213160_at	DOCK2	7.693	0.0004561	0.8467
209604_s_at	GATA3	7.65	0.000476	0.8467
209215_at	MFSD10	7.618	0.0004914	0.8467
218308_at	TACC3	7.421	0.0005986	0.8467
208132_x_at	BAT2	7.298	0.0006766	0.8467
201443_s_at	ATP6AP2	6.823	0.001089	0.8467
209052_s_at	WHSC1	6.786	0.001129	0.8467
203600_s_at	FAM193A	6.602	0.001358	0.8467
203054_s_at	TCTA	6.454	0.001574	0.8467
219401_at	XYLT2	6.409	0.001646	0.8467
43544_at	MED16	6.377	0.0017	0.8467
212696_s_at	RNF4	6.331	0.00178	0.8467
211594_s_at	MRPL9	6.273	0.001887	0.8467
208764_s_at	ATP5G2	6.266	0.001901	0.8467
220081_x_at	HSD17B7	6.211	0.002007	0.8467

Table 5.2: *(continued)*

Probesets	Symbol	F	P.Value	adj.P.Val
213416_at	ITGA4	6.138	0.002158	0.8467
202764_at	STIM1	6.097	0.00225	0.8467
212546_s_at	FRYL	5.937	0.002639	0.8467
203997_at	PTPN3	5.932	0.002654	0.8467
202771_at	FAM38A	5.858	0.002857	0.8467
34260_at	TELO2	5.831	0.002935	0.8467
204960_at	PTPRCAP	5.81	0.002996	0.8467
201923_at	PRDX4	5.72	0.003278	0.8467
218884_s_at	GUF1	5.631	0.003586	0.8467
205349_at	GNA15	5.589	0.003739	0.8467
220597_s_at	ARL6IP4	5.573	0.003799	0.8467
209234_at	KIF1B	5.552	0.003879	0.8467
202110_at	COX7B	5.493	0.004114	0.8467
204374_s_at	GALK1	5.491	0.004123	0.8467
213468_at	ERCC2	5.49	0.004129	0.8467
202014_at	PPP1R15A	5.482	0.00416	0.8467
201000_at	AARS	5.467	0.004224	0.8467
202496_at	EDC4	5.426	0.004403	0.8467
221620_s_at	APOO	5.367	0.004667	0.8467
222062_at	IL27RA	5.354	0.004729	0.8467
208658_at	PDIA4	5.345	0.00477	0.8467
212114_at	ATXN7L3B	5.332	0.004835	0.8467
218438_s_at	MED28	5.213	0.005443	0.8467
218600_at	LIMD2	5.18	0.005631	0.8467

6 Testing the *NCI60* LDA Classifier on the Arkansas Data

6.1 Predicting Melphalan Resistance Classes

First, the expression data are extracted from the expression set.

```
> Arkansas.matrix <- exprs(GEPArkansas)
```

Next, the expression set are ordered according to the LDA classifier,

```
> sortNCI60Arkansas <- Arkansas.matrix[probesets.NCI60, ]
```

and scaled so that each gene expression profile across the patients have unit variance and zero mean.

```
> sortNCI60Arkansas <-  t(scale(t(sortNCI60Arkansas)))
```

Finally, the resistance classes are predicted using the function `predict.sda` from the package `sda`

```
> predictDLDAArkansas <-
      predict(fitLDANCI60, t(sortNCI60Arkansas))
```

The predicted classes are summarized in Table 6.1.

Category	Number
Intermediate	309
Resistant	129
Sensitive	121

Table 6.1: Summary of the class predictions made by the *NCI60* LDA classifier.

6.2 Association between the Resistance Classes and OS

In the following code chunk Kaplan-Meier survival curves are created for the resistance classes. The result is shown in Figure 6.1.

```
> pdf(file.path(figure.output, "LDANCI60ArkansasKMplotOS.pdf"))
> par(mfrow = c(1, 1))
> metadataArkansas$DLDAThreshold <- predictDLDAArkansas$class
> p.NCI60.LDA.OS <- PlotKM.sda(predictDLDAArkansas$class, metadataArkansas$OS,
                               ylab    = "Overall Survival",
```

```
                                    col     = our.colscheme[order(names(our.colscheme))],
                                    xlab    = "Time (months)",
                                    legend  = names(our.colscheme),
                                    col.leg = our.colscheme,
                                    main    = paste("Arkansas", "\n",
                                    "NCI60 Kaplan-Meier OS Curves",sep=""))
> dev.off()
```

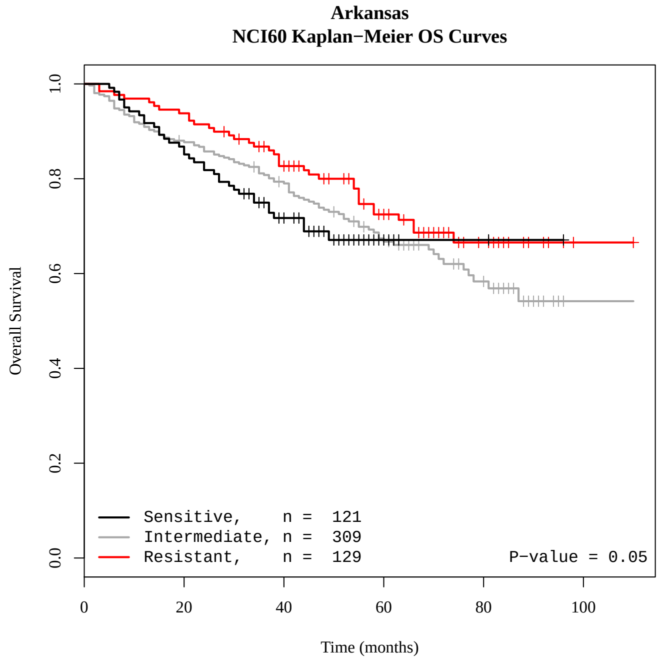

Figure 6.1: Kaplan-Meier survival curves based on the predicted *NCI60* LDA classes. The logrank test comparing the survival curves results in a P-value of 0.05.

6.3 Association between the Resistance Classes and EFS

In the following code chunk Kaplan-Meier survival curves are created for the predicted resistance classes. The result is shown in Figure 6.2.

```
> pdf(file.path(figure.output,"LDANCI60ArkansasKMplot.pdf"))
> par(mfrow = c(1, 1))
> metadataArkansas$DLDAThreshold <- predictDLDAArkansas$class
> p.NCI60.LDA.EFS <- PlotKM.sda(predictDLDAArkansas$class, metadataArkansas$EFS,
                                    ylab    = "Event Free Survival",
                                    col     = our.colscheme[order(names(our.colscheme))],
                                    xlab    = "Time (months)",
```

```
                    legend  = names(our.colscheme),
                    col.leg = our.colscheme,
                    main    = paste("Arkansas", "\n",
                    "NCI60 Kaplan-Meier EFS Curves",sep=""))
> dev.off()
```

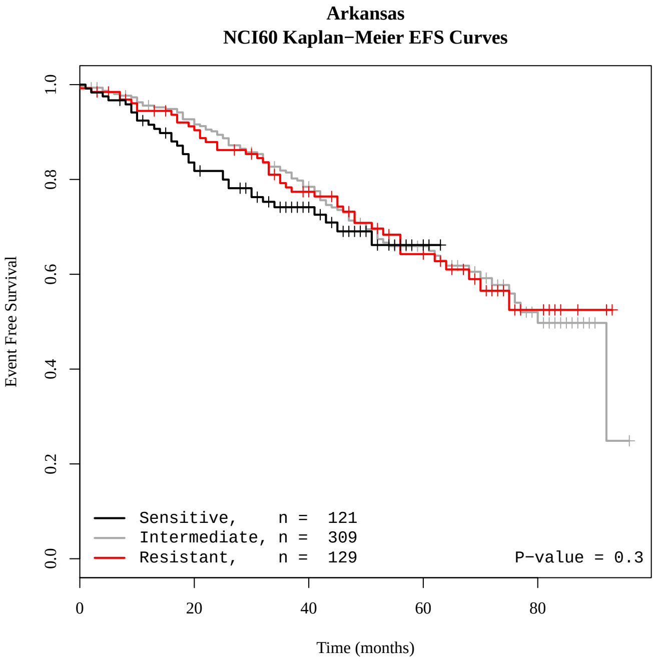

Figure 6.2: Kaplan-Meier survival curves based on the predicted *NCI60* LDA classes. The logrank test comparing the survival curves results in a P-value of 0.3.

7 Establishment of the *NCI60* SPLS Resistance Index

7.1 Cross-Validation

When the output variable is one dimensional, SPLS has two tuning parameters: the number of hidden components K and the shrinkage parameter η. Below, the two tuning parameters are determined through CV for a wide range of values for the parameter `var.cutoff` in the function `nsFilter`. The combination of K, η and `var.cutoff` which results in the minimum Mean Square Prediction Error (MSPE) is chosen for further analysis.

By selecting the expression profiles at random instead of using `nsFilter` it is possible to investigate whether the function choose expression profiles that perform better than random noise.

In order to avoid gene expression profiles which are not marginally related to melphalan resistance sure independence screening is used. Only gene expressions which are significantly correlated at the significance level specified below are used in the analysis.

```
> pval   <- 0.05
```

The P-values are not adjusted.

```
> adjust <- "none"
```

```
> set.seed(1000)
> NCI60.SPLS.crossval.res.file <-
      file.path(NCI60.gen.dir, "NCI60SPLScrossval.res.Rdata")
> if(file.exists(NCI60.SPLS.crossval.res.file)){
      load(NCI60.SPLS.crossval.res.file)
  }else {
      cutoffs <- seq(0.1, 0.98, by = 0.01)

      NCI60.SPLS.crossval.res <-
          data.frame(cutoffs = cutoffs,
                     eta  = 0, K=0,
                     mspe = 0, rand = 0)

      for(cutoff in cutoffs){

          NCI60.filtered <- nsFilter(GEPNCI60133a,
                                     var.cutoff = cutoff)$eset

          n.feat      <- length(featureNames(NCI60.filtered))
```

```
        rand.probes <- sample(length(featureNames(GEPNCI60133a)), n.feat)

        NCI60.random        <- exprs(GEPNCI60133a[rand.probes, ])

        eta <- seq(0.2, 0.99, length.out = 200)

        fit.spls.cv <-
            cv.spls(x        = t(NCI60.random),
                    y        = NCI60Resistanceindex,
                    fold     = length(NCI60Resistanceindex),
                    eta      = eta,
                    K        = c(1, 2, 3),
                    plot.it = FALSE,
                    do.SIS  = TRUE,
                    pval     = pval,
                    adjust  = adjust)

        NCI60.SPLS.crossval.res[NCI60.SPLS.crossval.res$cutoffs ==
                                cutoff, ]$rand <- min(fit.spls.cv$mspe)

        NCI60GEP <- exprs(NCI60.filtered)

        fit.spls.cv <-
            cv.spls(x        = t(NCI60GEP),
                    y        = NCI60Resistanceindex,
                    fold     = length(NCI60Resistanceindex),
                    eta      = eta,
                    K        = c(1, 2, 3),
                    plot.it = FALSE,
                    do.SIS  = TRUE,
                    pval     = pval,
                    adjust  = adjust)

        NCI60.SPLS.crossval.res[NCI60.SPLS.crossval.res$cutoffs ==
                                cutoff, ]$eta  <- fit.spls.cv$eta.opt
        NCI60.SPLS.crossval.res[NCI60.SPLS.crossval.res$cutoffs ==
                                cutoff, ]$K    <- fit.spls.cv$K.opt
        NCI60.SPLS.crossval.res[NCI60.SPLS.crossval.res$cutoffs ==
                                cutoff, ]$mspe <- min(fit.spls.cv$mspe)
    }
    save(NCI60.SPLS.crossval.res, file = NCI60.SPLS.crossval.res.file)
}
```

The result of the CV routine is extracted by the code chunk below and the optimal values

for these three parameters are shown in Table 7.1.

```
> mspe.min <-  NCI60.SPLS.crossval.res[NCI60.SPLS.crossval.res$mspe ==
                                   min(NCI60.SPLS.crossval.res$mspe), ]
> n.mspe   <- dim(mspe.min)[1]
> cutoff   <- mspe.min$cutoffs[n.mspe]
> K        <- mspe.min$K[n.mspe]
> eta      <- mspe.min$eta[n.mspe]
```

Parameter	Optimal value by CV
cutoff	0.6600
η	0.9265
K	3.0000

Table 7.1: Values minimzing the MSPE.

The code chunk below constructs a plot of the minimum MSPE achieved through each of the tested values of `var.cutoff` together with the minimum MSPE achieved through the randomly selected expression profiles.

```
> pdf(file.path(figure.output, "NCI60MSPEcomparison.pdf"))
>   plot(NCI60.SPLS.crossval.res$cutoffs,
         NCI60.SPLS.crossval.res$mspe,
         ylim = c(min(NCI60.SPLS.crossval.res$mspe,
                      NCI60.SPLS.crossval.res$rand),
                  max(NCI60.SPLS.crossval.res$mspe,
                      NCI60.SPLS.crossval.res$rand)),
         ylab = "MSPE", xlab = "Fraction filtered out", type = "n",
         main = "NCI60 MSPE")
>   lines(NCI60.SPLS.crossval.res$cutoffs,
          NCI60.SPLS.crossval.res$mspe, lty = 1)
>   lines(NCI60.SPLS.crossval.res$cutoffs,
          NCI60.SPLS.crossval.res$rand, lty = 2)
>   legend("topleft", lty = c(1, 2),
           legend = c("nsFilter", "Random"),
           bty    = "n")
>   dev.off()
```

The plot is shown in Figure 7.1. Setting `var.cutoff` equal to 0.66 results in the smallest minimum MSPE.

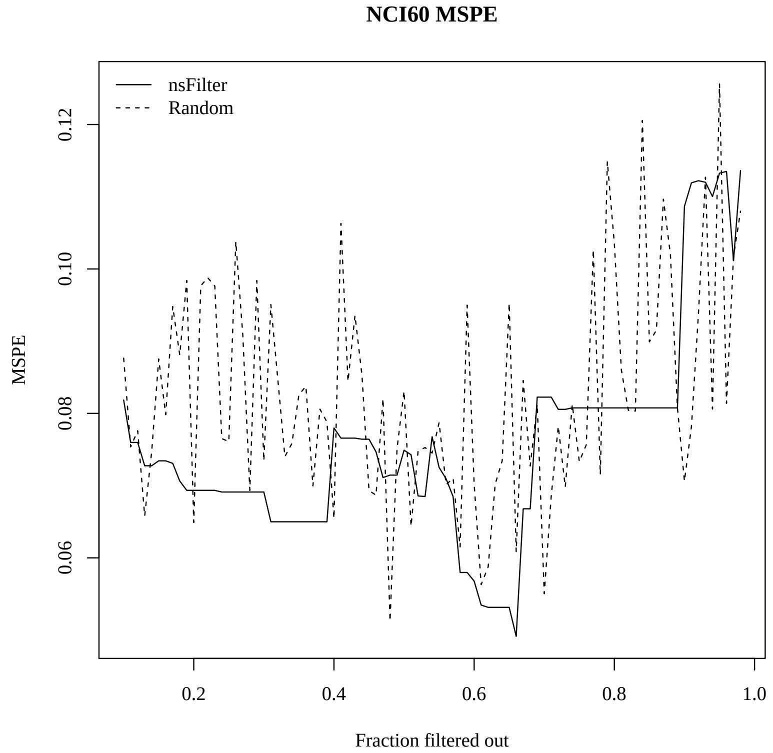

Figure 7.1: The minimum MSPE achieved through CV on K and η in SPLS for a variety of values for the var.cutoff. The smallest minimum MSPE is obtained with var.cutoff equal to 0.66

When setting `var.cutoff` equal to 0.66 the resulting CV on K and η is shown in Figure 7.2. In order to establish the plot filtering of the expression data with the parameter `var.cutoff` set equal to 0.66 is performed.

```
> NCI60.filtered <- nsFilter(GEPNCI60133a,
                             var.cutoff = cutoff)$eset
> GEPNCI60 <- exprs(NCI60.filtered)
```

When using the settings in `nsFilter` the 4311 most varying mRNA expression profiles are used for further analysis.

```
> NCI60MSPE.file <- file.path(figure.output, "NCI60MSPE.pdf")
> if(!file.exists(NCI60MSPE.file)){

     fit.spls.cv <-
         cv.spls(x       = t(GEPNCI60),
                 y       = NCI60Resistanceindex,
```

```
            fold     = n.NCI60,
            eta      = seq(0.5, 0.99, length.out = 100),
            K        = c(1, 2, 3),
            plot.it  = FALSE,
            do.SIS   = TRUE,
            pval     = pval,
            adjust   = adjust)

    pdf(file.path(figure.output, "NCI60MSPE.pdf"),
        width = 7, height = 7)

    trace.mspe(fit.spls.cv, header = "NCI60 MSPE")

    dev.off()
}
```

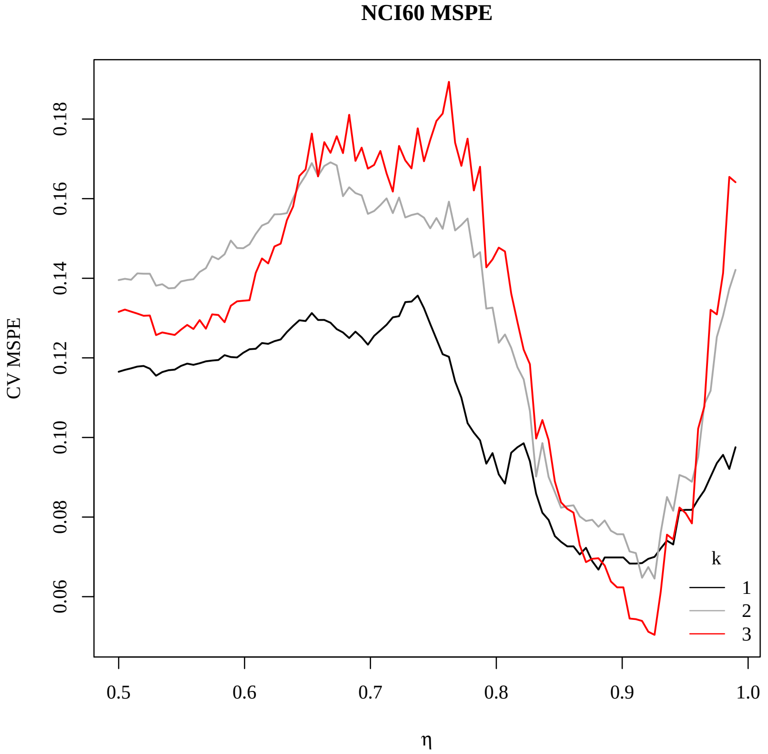

Figure 7.2: MSPE for the SPLS regression with var.cutoff in nsFilter set equal to 0.66. This leads to the selection of K = 3 hidden components and a sparsity parameter $\eta = 0.9265$.

In order to investigate the accuracy of the SPLS model with the specific settings the CV predicted resistance index is calculated once more and stored in the object `pred.1`.

```
> pred.1            <- matrix(NA, nrow = n.NCI60, ncol = 2)
> rownames(pred.1) <- NCI60.names.sorted
> colnames(pred.1) <- c("Measured", "predicted")
> pred.1[, 1]       <- NCI60Resistanceindex[NCI60.names.sorted, ]
> for(i in NCI60.names.sorted){

      GEPNCI60.cv <- exprs(NCI60.filtered[, sampleNames(GEPNCI60133a) %w/o% i])

      y <- NCI60Resistanceindex[sampleNames(GEPNCI60133a) %w/o% i, ]

      NCI60.fit.spls.cv <- spls(x       = t(GEPNCI60.cv),
                                y       = y,
                                K       = K,
                                eta     = eta,
                                do.SIS  = TRUE,
                                pval    = pval,
                                adjust  = adjust)

      newx <- exprs(GEPNCI60133a)[row.names(GEPNCI60.cv), i]

      pred.1[i, 2] <- as.vector(predict(NCI60.fit.spls.cv,
                                newx = t(as.matrix(newx))))

  }
```

In Figure 7.3A the predicted melphalan resistance index is plotted against the GI_{50} values. When the parameter `var.cutoff` in the function `nsFilter` is determined in this manner the resulting MSPE obtained through CV with SPLS is overoptimistic. In order to determine how well the model performs CV over the chosen parameters including `var.cutoff` is performed.

```
> pred.2            <- matrix(NA, nrow = n.NCI60, ncol = 3)
> rownames(pred.2) <- NCI60.names.sorted
> colnames(pred.2) <- c("Measured", "Predicted Naive", "Predicted")
> pred.2[, 1]       <- NCI60Resistanceindex[NCI60.names.sorted, ]
> pred.2[, 2]       <- pred.1[, 2]
> for(i in NCI60.names){
      NCI60.filtered.cv <-
          nsFilter(GEPNCI60133a[, sampleNames(GEPNCI60133a) %w/o% i],
                   var.cutoff = cutoff)$eset

      GEPNCI60.cv <- exprs(NCI60.filtered.cv)

      y <- NCI60Resistanceindex[sampleNames(GEPNCI60133a) %w/o% i, ]
```

```
NCI60.fit.spls.cv <- spls(x       = t(GEPNCI60.cv),
                          y       = y,
                          K       = K,
                          eta     = eta,
                          do.SIS  = TRUE,
                          pval    = pval,
                          adjust  = adjust)

newx <- exprs(GEPNCI60133a)[row.names(GEPNCI60.cv), i]

pred.2[i, 3] <- as.vector(predict(NCI60.fit.spls.cv,
                          newx = t(as.matrix(newx))))

}
```

In Figure 7.3B the predicted resistance index is plotted against the measured resistance index. As assumed the result is slightly worse than when the parameter `var.cutoff` is not included in the CV step.

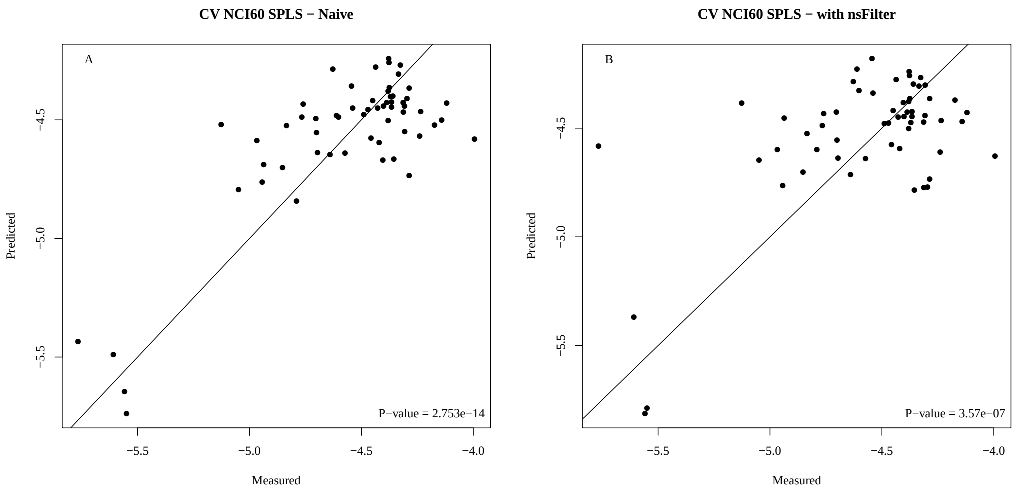

Figure 7.3: Predicted resistance index vs. the measured resistance index. The left panel shows CV after filtering. The right panel shows predictions where filtering is performed each time a cell line is left out.

7.2 Establishment of the Gene Expression Signature

In the previous section various values were determined through CV. In this section these values are used for fitting a gene expression signature. For this purpose the function `spls`

U133 ID	Gene Symbol	Mean	SD	Location	Weight	PMID
213416_at	ITGA4	4.644	1.266	2q31.3	−0.183	0
218840_s_at	NADSYN1	6.944	1.382	11q13.4	−0.139	0
209604_s_at	GATA3	4.487	1.244	10p15	−0.106	7
213160_at	DOCK2	5.639	0.548	5q35.1	−0.091	0

Table 7.2: Gene symbol, location and weight for the 4 probes identified by use of the NCI60 panel in combination with SPLS regression.

from the package `spls` is used.

The gene expression signature is established and saved for later resistance index predictions.

```
> NCI60.fit.spls <- spls(x      = t(GEPNCI60),
                         y      = NCI60Resistanceindex,
                         K      = K,
                         eta    = eta,
                         do.SIS = TRUE,
                         pval   = pval,
                         adjust = adjust)
> save(NCI60.fit.spls,
       file = file.path("Generated data", "NCI60", "NCI60.fit.spls.Rdata"))
```

7.3 Investigation of the chosen Gene Expression Profiles

Only 4 mRNA expression profiles are used in the signature. In the code chunk below the gene symbol for these 4 mRNA expression profiles is looked up. Furthermore, in order to see if these mRNA profiles have been observed in other papers studying chemoresistance the following search is conducted in PubMed.

```
> resTable  <- lookUpPubmed(NCI60.fit.spls, n.coef = 3)
```

The results of the search are shown in Table 7.2.

In order to investigate how the identified probes relate to chemoresistance marginally correlation coefficients are calculated and the association between gene expression and resistance index is plotted in Section 7.4.

```
> NCI60Coef <- coef(NCI60.fit.spls)
> NCI60Coef <- NCI60Coef[NCI60Coef != 0,]
> NCI60Coef <- round(NCI60Coef[order(NCI60Coef)], 4)
> if(length(NCI60Coef) == 1) {
      corm <- t(as.matrix(GEPNCI60[names(NCI60Coef), ]))
      row.names(corm) <- names(NCI60Coef)
```

```
}else{
    corm <- GEPNCI60[names(NCI60Coef), ]
}
> for(i in 1:length(NCI60Coef)){
    pdf(paste(figure.output, "/", i, "NCI60correlations.pdf", sep = ""),
        width = 7, height = 7)

    plot(NCI60Resistanceindex ~ corm[i, ],
         ylab = "Resistance index",
         xlab = "Gene expression",
         type = "n")

    title(unlist(lookUp(rownames(corm)[i],
                        "hgu133plus2", "SYMBOL")))

    text(corm[i, ], NCI60Resistanceindex,
         names(unlist(lookUp(colnames(corm), "hgu133plus2",
                             "SYMBOL"))), cex = 0.5)
    legend("bottomleft",
           title = paste("     Cor =", as.character(round(
           cor(NCI60Resistanceindex, corm[i, ]), 2))),
           legend = "", bty = "n", cex = 0.5)
    dev.off()
}
```

7.4 Plots of Marginal Associations

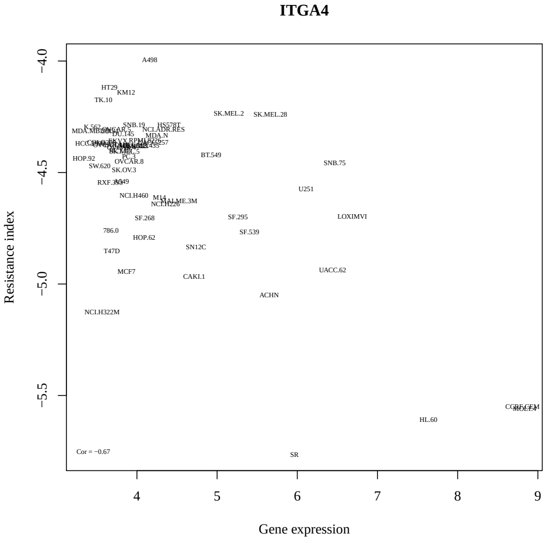

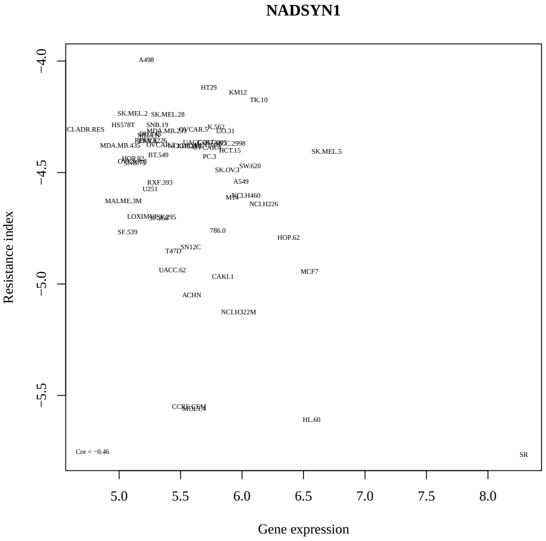

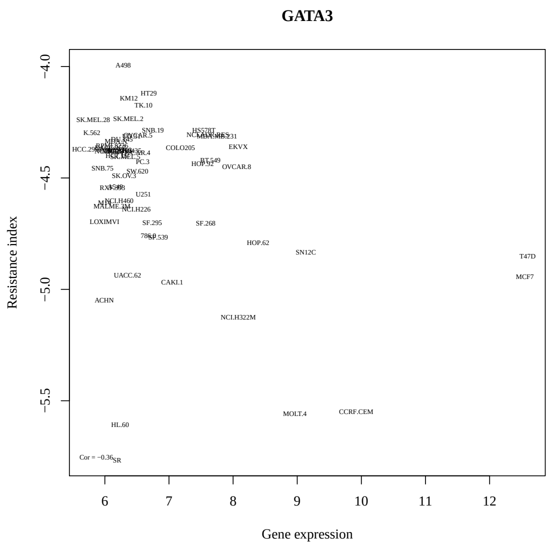
GATA3
Resistance index
Gene expression
Cor = −0.36

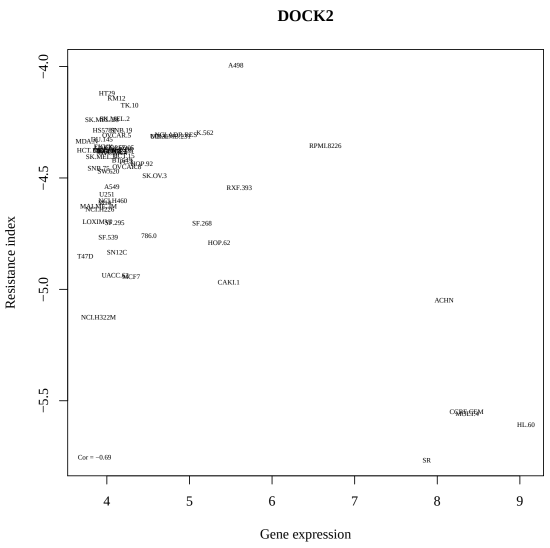
DOCK2
Resistance index
Gene expression
Cor = −0.69

8 Testing the *NCI60* SPLS Resistance Index on the Arkansas Data Set

8.1 Predicting the Resistance Index in the *Arkansas* data

Firstly, the expression data are extracted from the expression set.

```
> arkansas.matrix <- exprs(GEPArkansas)
```

Next, the probes which were removed by `nsFilter` with `var.cutoff` set equal to 0.66 are discarded from the matrix.

```
> NCI60ArkansasTest <-
      arkansas.matrix[featureNames(NCI60.filtered), ]
```

Finally, the signature is used to predict each patient's resistance index.

```
> NCI60Arkansasindex <- as.vector(predict(NCI60.fit.spls,
                                          newx = t(NCI60ArkansasTest)))
```

8.2 Association between the Resistance Index and OS

Kaplan-Meier Survival Curves

Kaplan-Meier survival curves are constructed by catergorizing the patients as being either sensitive, intermediate or resistant. The 25% with the lowest predicted melphalan resistance index are categorised as sensitive and the 25% with the highest predicted melphalan resistance index are categorised as resistant. The remaining subjects are characterized as having intermediate resistance.

The Kaplan-Meier survival curves are plotted with the code chunk below and the result is shown in Figure 8.1.

```
> pdf(file.path(figure.output,"NCI60arkansasOSKMplot.pdf"))
> NCI60arkansasOSKM.P <-
      PlotKM(NCI60Arkansasindex,metadataArkansas$OS,
             cut.points = cut.points,
             xlab       = "Time (months)",
             ylab       = "OS ratio",
             xmax       = 110,
             main       = "Arkansas \n NCI60 Kaplan-Meier OS curves"
             )
> dev.off()
```

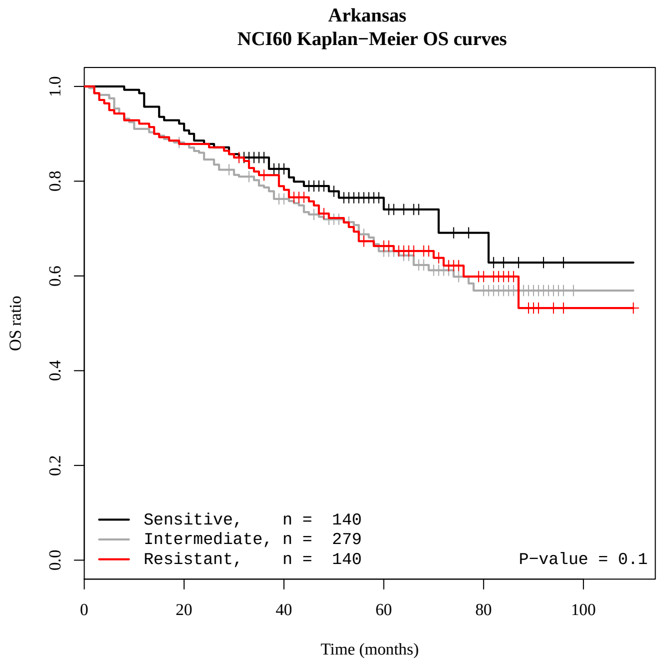

Figure 8.1: Kaplan-Meier survival curves based on the *NCI60* resistance index. The logrank test comparing the survival curves results in a P-value of 0.1.

8.2.1 Cox Proportional Hazards

A Cox proportional hazards model is fitted where the association between log relative hazard and the resistance index is modelled by a spline with four knots.

```
> n.knots <- 4
> coxfit.os <- coxph(metadataArkansas$OS ~
                     rcs(NCI60Arkansasindex, n.knots))
```

Table 8.1 summarizes three tests for no association between the log relative hazard and the resistance index.

Test	Test	D.o.F	P-value
Likelihood ratio test	3.72	3	0.293
Wald test	3.20	3	0.362
Score (log rank) test	3.23	3	0.357

Table 8.1: A summary of three tests for no association between the log relative hazard and the resistance index.

The code chunk below produces a plot of the log relative hazard as a function of the *NCI60* resistance index. The result is depicted in Figure 8.2.

```
> pdf(file.path(figure.output,"NCI60arkansasOSPredictors.pdf"))
> par(mfrow=c(1,1))
> d <- datadist(NCI60Arkansasindex)
> options(datadist = "d", width = 150)
> NCI60f <- cph(metadataArkansas$OS ~ rcs(NCI60Arkansasindex,4))
> plot(NCI60f,
       xlab = "Fitted NCI60 resistance index")
> title("Arkansas \n NCI60 - OS Cox Proportional Hazards")
> legend("bottomright",
         bty    = "n",
         legend = paste("P-value = ",
         as.character(signif(unlist(NCI60f)$stats.P, 1)), sep = ""))
> dev.off()
```

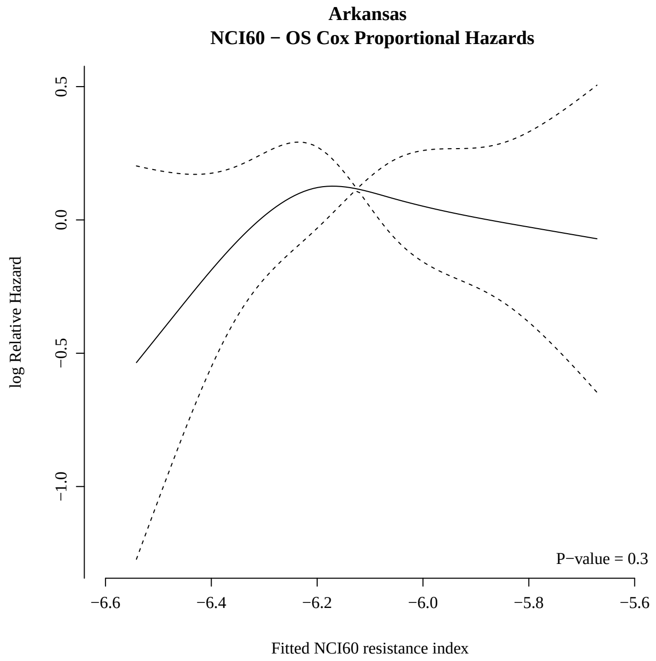

Figure 8.2: The log relative hazard as a function of the *NCI60* resistance index. The P-value is the likelihood ratio test for no RCS-association between the log relative hazard and the resistance index. The dashed lines represent 95% confidence intervals.

8.2.2 Association between the Resistance Index and EFS

Kaplan-Meier Survival Curves

Similar to the previous section Kaplan-Meier survival curves are made with the patients grouped to be sensitive, intermediate or resistant, however, EFS is used as endpoint.

The Kaplan-Meier survival curves are plotted with the code chunk below and the result is shown in Figure 8.3.

```
> pdf(file.path(figure.output,"NCI60arkansasEFSKMplot.pdf"))
> NCI60arkansasEFSKM.P <-
      PlotKM(NCI60Arkansasindex,metadataArkansas$EFS,
             cut.points = cut.points,
             xlab       = "Time (months)",
             ylab       = "EFS ratio",
             xmax       = 110,
             main       = "Arkansas \n  NCI60 Kaplan-Meier EFS curves")
> dev.off()
```

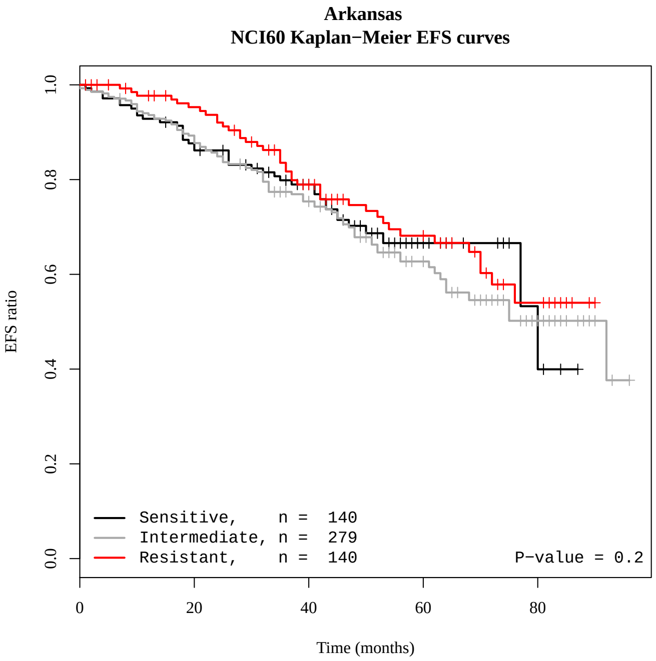

Figure 8.3: Kaplan-Meier survival curves based on the *NCI60* resistance index. The logrank test comparing the survival curves results in a P-value of 0.2.

Cox Proportional Hazards

A Cox proportional hazards model is fitted were the association between log relative hazard and the resistance index is modelled by a spline with four knots.

```
> n.knots   <- 4
> coxfit.os <-  coxph(metadataArkansas$EFS ~
                  rcs(NCI60Arkansasindex, n.knots))
```

Table 8.2 summarises three tests for no association between the log relative hazard and the resistance index.

Test	Test	D.o.F	P-value
Likelihood ratio test	0.263	3	0.967
Wald test	0.260	3	0.968
Score (log rank) test	0.259	3	0.967

Table 8.2: A summary of three tests for no association between the log relative hazard and the resistance index.

The code chunk below produces a plot of the log relative hazard as a function of the *NCI60* resistance index. The result is depicted in Figure 8.4.

```
> pdf(file.path(figure.output, "NCI60arkansasEFSPredictors.pdf"))
> par(mfrow = c(1, 1))
> d <- datadist(NCI60Arkansasindex)
> options(datadist = "d", width = 150)
> NCI60f <- cph(metadataArkansas$EFS ~ rcs(NCI60Arkansasindex, n.knots))
> plot(NCI60f, xlab = "Fitted NCI60 resistance index")
> title("Arkansas \n NCI60 - EFS Cox Proportional Hazards")
> legend("bottomright",
         bty    = "n",
         legend = paste("P-value = ",
         as.character(signif(unlist(NCI60f)$stats.P, 1)), sep = ""))
> dev.off()
```

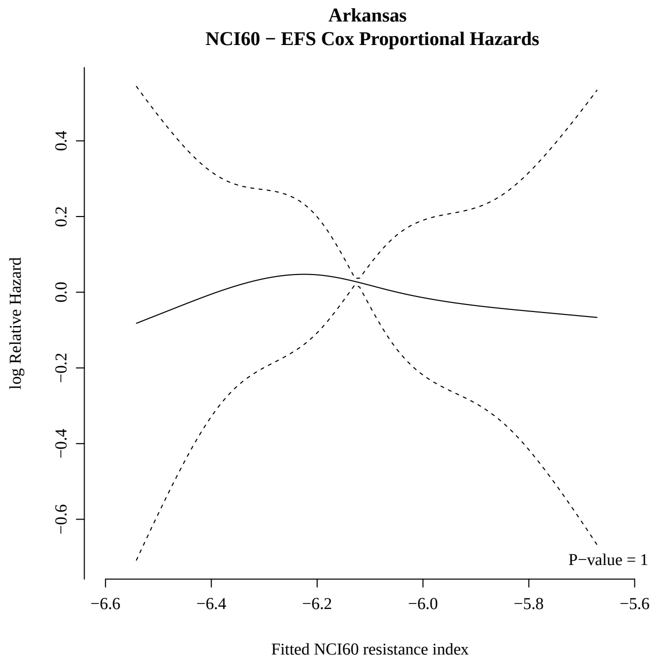

Figure 8.4: The log relative hazard as a function of the *NCI60* resistance index. The P-value is the likelihood ratio test for no RCS-association between the log relative hazard and the resistance index. The dashed lines represent 95% confidence intervals.

9 Establishment of the *BCell* Gene Expression Data

Firstly, various directories are specified.

```
> BCell.ext.dir <- paste(getwd(), "/External data/Bcell",   sep = "")
> BCell.gen.dir <- paste(getwd(), "/Generated data/BCell/", sep = "")
> figure.output <- paste(getwd(), "/Output/Figures",        sep = "")
> table.output  <- paste(getwd(), "/Output/Tables",         sep = "")
```

The three output directories are created by the code chunk below.

```
> dir.create(path=BCell.gen.dir,
           showWarnings = FALSE, recursive = TRUE, mode = "0777")
> dir.create(path=figure.output,
           showWarnings = FALSE, recursive = TRUE, mode = "0777")
> dir.create(path=table.output,
           showWarnings = FALSE, recursive = TRUE, mode = "0777")
```

The gene expression arrays used for the analysis are specified in the metadata. This is used to construct a phenoData object.

```
> file <-
      paste(BCell.ext.dir, "/Metadata/metadata_MM.csv", sep = "")
> metadata           <- read.csv2(file)
> rownames(metadata) <- metadata$Name
> phenoData          <- new("AnnotatedDataFrame", data = metadata)
```

9.1 RMA Normalisation of the HG-U133 Plus 2.0 Arrays

```
> file <- paste(BCell.gen.dir, "GEPBCell.RMA.Rdata", sep = "")
> if(file.exists(file)){
      load(file)
  }else{
      fns <-
          paste(getwd(), "/External data/BCell/Celfiles/",
                metadata$Filename, ".CEL", sep = "")
      GEPBCell.RMA <- just.rma(filenames = fns,
                               phenoData = phenoData)
      save(GEPBCell.RMA, file=file)
  }
> n.BCell <- dim(GEPBCell.RMA)[2]
> p.BCell <- dim(GEPBCell.RMA)[1]
```

Write GEPBCell.rma to a space separated .txt file, used for GEO upload.

```
> write.table(exprs(GEPBCell.RMA),
              file = paste(table.output,"/GEPBCell.RMA", ".txt", sep = ""),
              sep = "\t")
```

This results in an expression set with 54675 features and 18 samples.

Unsupervised clustering

The microarrays of the *BCell* panel were prepared in 5 batches numbered subsequently from 1 to 5. The cell line gene expression profiles were compared using hierarchical cluster analysis with average linkage as the agglomeration method and the Euclidean norm as distance measure. The result is shown in Figure 9.1.

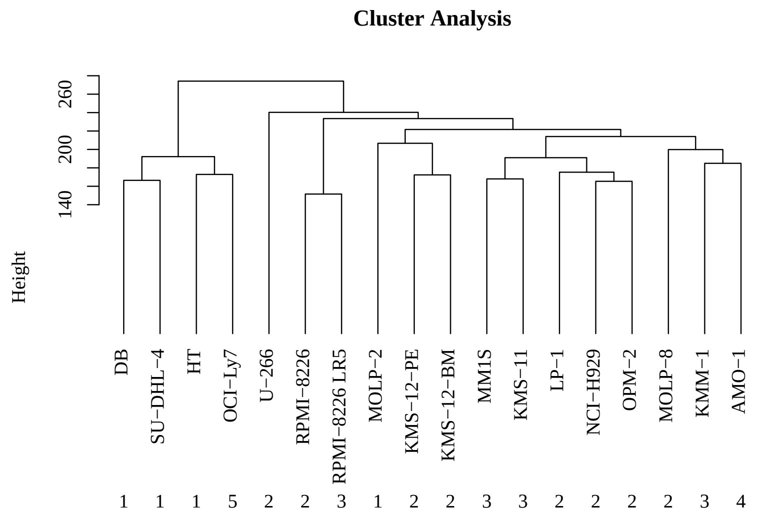

Figure 9.1: Hierarchical clustering of the 18 cell lines. The numbers annotated below each branch represents batch runs of the cell lines.

The four cell lines *OCI-Ly7*, *DB*, *HT*, and *SU-DHL-4* originate from patients with DLBCL. The two cell lines *RPMI-8226* and *RPMI-8226 LR5* originate from the same person as do the two cell lines *KMS-12-BM* and *KMS-12-PE*. The cell lines clustered together showing the existence of an intra person correlation. We conclude that no severe batch effects can be detected from the present analysis.

9.2 Restriction to the Affymetrix HG-U133A Subset

```
> GEPBCell133a <- GEPBCell.RMA[intersect(hgu133aprobe$Probe.Set.Name,
                                         featureNames(GEPBCell.RMA)), ]

> write.table(exprs(GEPBCell133a),
              file = paste(table.output,"/GEPBCell133a", ".txt", sep = ""),
              sep = "\t")
```

10 Establishment of the *BCell* Dose Response Data

When a cell line is exposed to melphalan the majority of the cells will be resistant to the drug until the dose reaches a certain threshold. A dose of the drug beyond this threshold causes an abrupt decline in the number of living cells. In this chapter the dose response data for the BCell panel is loaded into R and the GI_{50} values are calculated. The plates which have been used for the analysis are specified in the metadata. This is used to construct a phenoData object. All information regarding the 96 well microtiter plates used for the dose response experiments are stored in the following metadata

```
> file <- paste(BCell.ext.dir,"/Metadata/",
                "Cell_lines_18_doses_Melphalan_Metadata.csv", sep = "")
> metadata <- read.csv2(file)
```

A pilot study was conducted in order to give an idea of the necessary span in the doses to ensure that the threshold is included. The 18 doses, denoted C1,...,C18, used to cover this span are shown in Table 10.1. The strongest dose is C18 and C17 is made by halfing this dose, similarly C16 is made by halfing C17 and so on, until reaching the weakest dose C1. Every time such a halfing is made an error is introduced, and to minimise this source of error the doses are split into three series: C18,...,C13, C12,...,C7, and C6,...,C1 where the doses C12 and C6 are made directly from C18. These three series are represented by the three sets of columns in Table 10.1.

Label	Dose	Label	Dose	Label	Dose
C18	60.00	C12	0.937500	C6	0.014648
C17	30.00	C11	0.468750	C5	0.007324
C16	15.00	C10	0.234375	C4	0.003662
C15	7.500	C9	0.117188	C3	0.001831
C14	3.750	C8	0.058594	C2	0.000916
C13	1.875	C7	0.029297	C1	0.000458

Table 10.1: The 18 different doses (in µg/ml) used for each dose response experiment. The strongest and weakest doses are denoted C18 and C1, respectively.

The Plate Setup

In order to measure the response of a cetain cell line, a culture plate with 96 wells is used. The standard setup for such a culture plate is depicted in Table 10.2. The wells marked C0,...,C18 contain cell suspension from the examined cell line and the 18 different doses of melphalan. At time t_0 cell suspension is added to wells labelled C0,...,C18 and

medium is added to the first and last column of the plate, labelled M and the wells labelled B.

The different dilutions of melphalan depicted in Table 10.1 are added to the respectable wells 24 hours later, at time t_1. At this point salt water is added to wells labelled C0 and B, while nothing is added to wells labelled M.

	1	2	3	4	5	6	7	8	9	10	11	12
A	M	C0	C2	C4	C6	C8	C10	C12	C14	C16	C18	M
B	M	C0	C2	C4	C6	C8	C10	C12	C14	C16	C18	M
C	M	C0	C2	C4	C6	C8	C10	C12	C14	C16	C18	M
D	M	C0	C2	C4	C6	C8	C10	C12	C14	C16	C18	M
E	M	C1	C3	C5	C7	C9	C11	C13	C15	C17	B	M
F	M	C1	C3	C5	C7	C9	C11	C13	C15	C17	B	M
G	M	C1	C3	C5	C7	C9	C11	C13	C15	C17	B	M
H	M	C1	C3	C5	C7	C9	C11	C13	C15	C17	B	M

Table 10.2: The standard set-up for the culture plates. The labelling of the wells is interpreted as: Wells labelled M contain medium alone, wells labelled C0 contain cell culture and salt water is added at time t_1, wells labelled C1,...,C18 contain cell culture and drug dilutions corresponding to Table 10.1 are added at time t_1, and finally wells labelled B contain medium alone and added salt water at time t_1.

The plate is harvested 48 hours later, at time t_2. When a plate is harvested the measured absorbance for each well containing cell culture is denoted $A_{c,k}$, where $c = 0,\ldots,18$ and $k = 1,\ldots,4$ denotes the concentration of melphalan and replica, respectively. The absorbance measured for the background is denoted B_k.

The result of such an experiment is stored in a .DBF file and the following code chunk reads all the .DBF files associated with the analysis.

```
> chemo.index <- readDBFMeta(file.path(BCell.ext.dir, "Doseresponse"),
                             metadata = metadata)
> excluded <- createGIData(chemo.index)[[2]]
```

The measurements in row A and B were removed to avoid any potential border effect. A Grubbs test was applied on every remaining triplicates and outliers were expunged from the dataset. A significance level of 0.01 leads to the exclusion of 34/7200 wells.

10.1 Calculation of the Percentage Growth

In order to calculate the GI_{50} values the percentage growth (PG) needs to be calculated first. When calculating the percentage growth the absorbance of the wells C0 is compared with the absorbance of the wells C1,...,C18.

The four wells labelled B in Table 10.2 do not contain any cellular material, and instead of adding melphalan at time t_1 salt water is added. Thus, the four wells indicate the plate

specific background absorbance. We assume that the background absorbance is additive.

Let

$$\bar{A}_{c,\cdot} = \frac{1}{3}\sum_{k=1}^{3} A_{c,k} \quad \text{and} \quad \bar{B}_{\cdot} = \frac{1}{3}\sum_{k=1}^{3} B_k$$

denote the average of the measured absorbance for the wells containing cell suspension and background wells, respectively.

We have chosen to calculate the percentage growth at concentration c as

$$PG_c = \frac{\bar{A}_{c,\cdot} - \bar{B}_{\cdot}}{\bar{A}_{0,\cdot} - \bar{B}_{\cdot}} \times 100. \tag{10.1}$$

This is done by the following function.

```
> GIdata    <- createGIData(chemo.index)[[1]]
```

The results are shown in Figure 10.1 where the replicated runs of the 18 cell lines are plotted in separate panels.

```
> key.variety <-
          list(space   = "top",
               text    = list(levels(GIdata$platerep)),
               points  = list(pch = c(17, 18, 15),
               col     = "black"),
               title   = "Replica",
               columns = 3)
> plot <- (xyplot((GI * 100) ~ t|name,
                  data       = GIdata,
                  groups     = platerep,
                  key        = key.variety,
                  lty        = 1,
                  pch        = c(17, 18, 15),
                  col.line   = "black",
                  col.symbol = "black",
                  col.grid   = "black",
                  scales     = list(x = list(log = 2)),
                  layout     = c(3, 6),
                  height     = 10,
                  width      = 20,
                  aspect     = 0.65,
                  type       = c("b","g"),
                  ylab       = "Per cent of Control",
```

```
                    xlab        = expression(paste(
                                  "Concentration (",mu,"g/ml)")),
                    main        = paste("Growth Inhibition for Cell Lines",
                                  "Treated with Melphalan")))

> pdf(file.path(figure.output, "complete_data.pdf"),
      width = 6, height = 8.5)
> print(plot)
> dev.off()
```

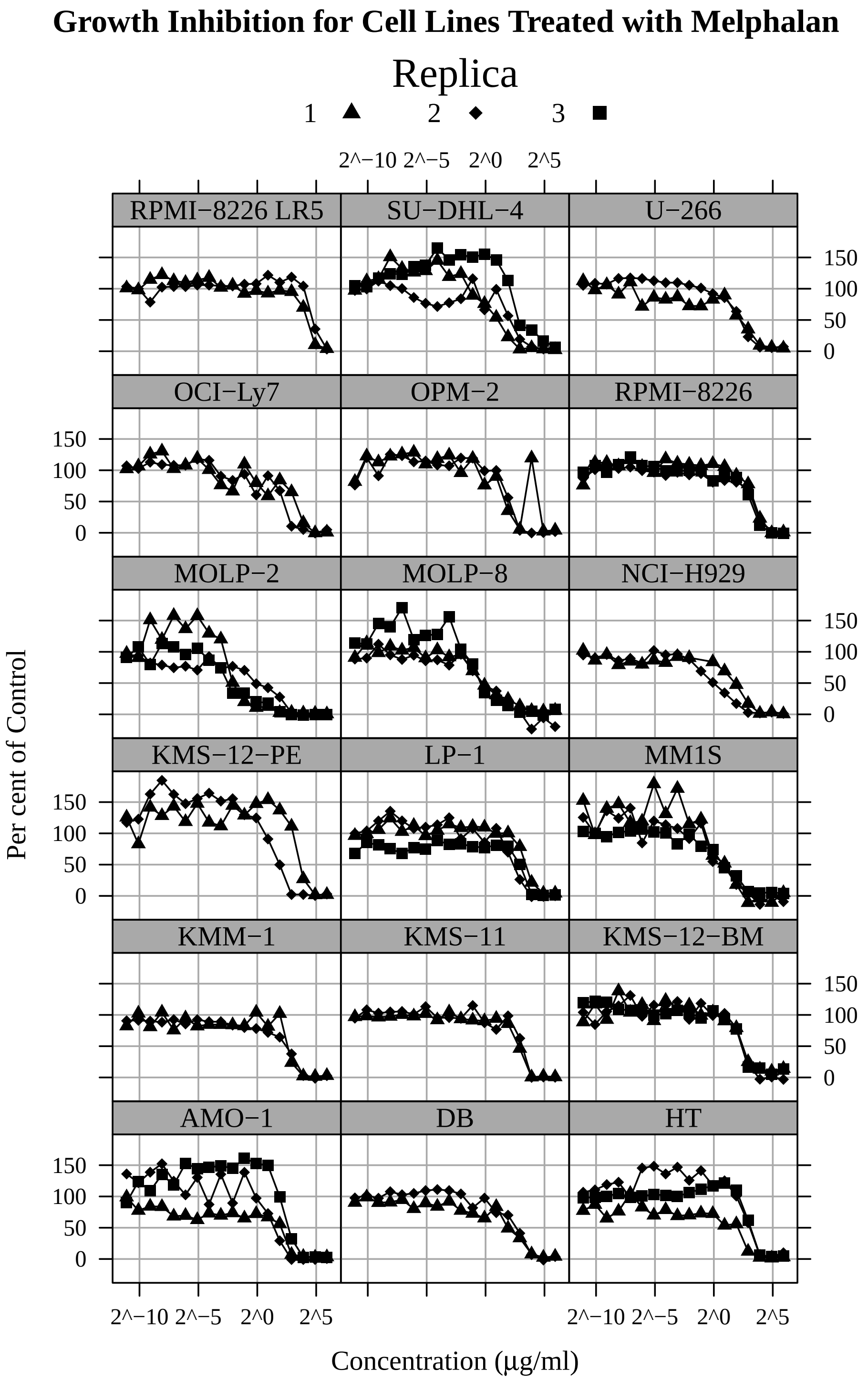

Figure 10.1: The result of replicated dose response runs of the cell lines are plotted in separate panels.

It seems that the following five points are outliers and are removed prior to the calculation of GI_{50} values.

```
>        GIdata <- GIdata[!(GIdata$name     == "OPM-2"     &
                            GIdata$platerep == 1 &
                            GIdata$GIname   == 16 ), ]
>         GIdata <- GIdata[!(GIdata$name     == "MM1S"      &
                            GIdata$platerep == 1 &
                            GIdata$GIname   == 7), ]
>         GIdata <- GIdata[!(GIdata$name     == "MM1S"      &
                            GIdata$platerep == 1 &
                            GIdata$GIname   == 9 ), ]
>         GIdata <- GIdata[!(GIdata$name     == "MOLP-2"    &
                            GIdata$platerep == 1 &
                            GIdata$GIname   == 7 ), ]
>         GIdata <- GIdata[!(GIdata$name     == "KMS-12-PE" &
                            GIdata$platerep == 1 &
                            GIdata$GIname   == 9 ), ]

> GIdata      <- GIdata[GIdata$t != 0, ]
> GIdata$name <- as.factor(as.character(GIdata$name))
```

10.2 Calculation of the GI_{50} values

In order to calculate the GI_{50} value for each cell line linear interpolation between the 18 PG values are chosen. The first dose at which the interpolation is equal to 50% is used as the GI_{50} value.

When applying this method the PG values of the replicated dose response experiments are summarized as the mean response at each concentration. Let $PG_{i,j,c}$ be the calculated percentage growth for the cth drug concentration of the jth replica within the ith cell line, and

$$\overline{PG}_{i,\cdot,c} = \frac{1}{k}\sum_{j=1}^{k} PG_{i,j,c},$$

where k represents the number of replicates for the ith cell line. In the code chunk below a dataset consisting only of these mean values is created.

```
> GImean         <- aggregate(GIdata$GI,
                            list(GIdata$name, GIdata$t),
                            FUN = mean)
> names(GImean) <- c("name", "t", "GI")
> GInames        <- levels(GImean$name)
```

In order to compare the results obtained through the BCell panel with those obtained with the NCI60 panel the GI_{50} values are transformed to the $\log_{10}$ mM scale. This is done by the following calculation:

$$\log_{10}(\text{mmol/ml}) = \log_{10}\left(\frac{1000 \cdot (\mu\text{g/ml})}{305.2} / 1000000\right) \tag{10.2}$$

```
> GImean$t.m <- molaer(GImean$t)
```

The averaged transformed percentage growth values are shown in Figure 10.2A for each cell line along with the linear interpolation. The figure is produced by the code chunk below.

```
> pdf(paste(figure.output, "/BCellDosecurves.pdf", sep = ""),
      width = 10, height = 7)
```

The settings for the plot are determined.

```
> matplot(c(min(GImean$t.m),    max(GImean$t.m)),
          c(min(GImean$GI * 100), max(GImean$GI) * 100),
          type      = "n",
          xlab      = expression(paste("Concentration ",
                        log[10], "(", m,"mol/ml)", sep = "")),
          ylab      = "Percent of Control", las = 1,
          main      = paste("Melphalan Induced Growth Inhibition", sep = ""),
          cex.axis = 0.7)
> # The five most sensitive are coloured black,
> # the eight intermediate are coloured dark grey
> # and the five most resistant are coloured red
> col <- c(rep(our.colscheme[1], 5),
           rep(our.colscheme[2], c(n.BCell - 5 * 2)),
           rep(our.colscheme[3], 5))
> # Six different line types are used
> lty <- c(1:5, 1:c(n.BCell - 5 * 2), 1:5)
> names <-  levels(GImean$name)
> # The lines are drawn
> h <- 1
> for(i in names){
      lines(GImean$t.m[GImean$name == i],
            GImean$GI [GImean$name == i] * 100,
            col = col[h], lty = lty[h], lwd = 1.4)
      h <- h + 1
  }
> # A legend,  ordered according to sensitivity,  is inserted
> legend("bottomleft", names, col = col,
         lty = lty, cex = 0.7, bty = "n")
```

```
> dev.off()
```

Linear interpolation between the PG values is used to extract the GI_{50} values. In the following the first point at which the dose response curve drops below the 50% per cent level is used as the GI_{50} value. The results are summarized in Table 11.1.

```
> GInames  <- levels(GImean$name)
> GIvalues <- rep(0, length(GInames))
> names(GIvalues) <- GInames
> for(j in GInames){
      GIvalues[j] <- findGI(GImean$t.m[GImean$name == j],
                            GImean$GI [GImean$name == j],
                            GI = 0.5)
  }
> BCellResistanceindex <- GIvalues
```

When the BCell resistance index is used to establish a gene expression signature the mRNA profiles stored in the expression set `GEPBCell133a` are used as input variables. It is therefore necessary to align the objects.

```
> BCell.names <- sampleNames(GEPBCell133a)
> BCellResistanceindex <- cbind(BCellResistanceindex[BCell.names])
> save(BCellResistanceindex, file=paste(BCell.gen.dir,
                              "/BCellResistanceindex.Rdata", sep = ""))
> BCell.names.sorted <- names(BCellResistanceindex[order(BCellResistanceindex),])
```

10.3 Subsampling with Replacement

In order to asses the uncertainty of the estimates we have chosen to use subsampling with replacement of the replicated wells and recalculation of the GI_{50} values in each dataset 200 times is used.

```
> file <- c(paste(BCell.gen.dir,"/bs.Rdata",sep=""))
> if(file.exists(file)){
      load(file)
  }else{
      set.seed(1000)
      bs <- matrix(NA, ncol = n.BCell, nrow = n.subsamples)
      colnames(bs) <- levels(GImean$name)
      for(i in 1:n.subsamples) {
          GIdata <- createGIData(chemo.index, Bootstrap = TRUE)[[1]]
      #============================================================
      # The outliers which were removed from the original data are
      # also removed here
```

```
        GIdata <- GIdata[!(GIdata$name     == "OPM-2"     &
                           GIdata$platerep == 1 &
                           GIdata$GIname   == 16 ), ]

        GIdata <- GIdata[!(GIdata$name     == "MM1S"      &
                           GIdata$platerep == 1 &
                           GIdata$GIname   == 7), ]

        GIdata <- GIdata[!(GIdata$name     == "MM1S"      &
                           GIdata$platerep == 1 &
                           GIdata$GIname   == 9 ), ]

        GIdata <- GIdata[!(GIdata$name     == "MOLP-2"    &
                           GIdata$platerep == 1 &
                           GIdata$GIname   == 7 ), ]

        GIdata <- GIdata[!(GIdata$name     == "KMS-12-PE" &
                           GIdata$platerep == 1 &
                           GIdata$GIname   == 9 ), ]
    #=================================================================
        GImean.bs <- aggregate(GIdata$GI,
                               list(GIdata$name, GIdata$t),
                               FUN=mean)

        names(GImean.bs) <- c("name","t","GI")
        GImean.bs$t.m    <- molaer(GImean.bs$t)
        GIvalues         <- rep(0,length(GInames))
        names(GIvalues)  <- GInames

        for(j in GInames){
            GIvalues[j] <- findGI(GImean.bs$t.m[GImean.bs$name == j],
                                  GImean.bs$GI [GImean.bs$name == j],
                                  GI = 0.5)
        }

        bs[i,] <- GIvalues
        print(i)
    }

    save(bs, file = file)
}
```

Figure 10.2B summarizes the result of the 200 GI_{50} values for each cell line in a box plot.

```
> pdf(paste(figure.output, "/BCellBoxplots.pdf", sep = ""),
      width = 7, height = 7)

> boxplot(bs, las = 2, cex.axis = 0.7,
          main = "BCell Resistance Index",
          ylab = expression(paste("Concentration ",
                 log[10], "(", m,"mol/ml)", sep = "")),
          pch = 18, col = col)

> dev.off()

windows
      2
```

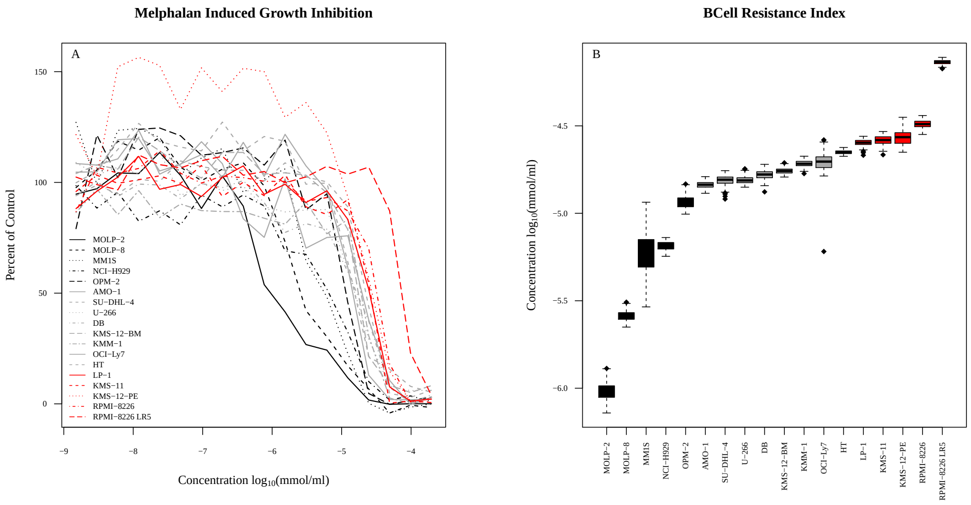

Figure 10.2: The average PG values are shown in plot A for each cell line in the *BCell* panel along with linear interpolation. Plot B shows a box plot of the GI_{50} values obtained for each cell line from each of the 200 bootstrapped datasets through linear interpolation.

11 Establishment of the *BCell* LDA Classifier

Setting the seed to ensure repeatability of the results.

```
> set.seed(1000)
```

The LDA analysis includes all gene expressions which are expressed significantly different between the sensitive, intermediate and resistant cell lines at an significance level of 0.05.

```
> pval <- 0.05
```

11.1 Definition of the *BCell* Resistance Classes

Defininining and annotating the sensitive, intermediate and resistant cell lines.

```
> sens <- 5
> resi <- 5
> inte <- n.BCell - sens - resi
> class.list <-  c(rep("Sensitive",    sens),
                   rep("Intermediate", inte),
                   rep("Resistant",    resi))
```

A data frame is constructed containing the GI_{50} values and the class of each cell line. The data frame is shown in Table 11.1

```
> sort.BCell.class <-
    data.frame(BCellResistanceindex = sort(BCellResistanceindex),
               class = class.list)
```

Cell line	BCellResistanceindex	class
MOLP-2	-6.0210	Sensitive
MOLP-8	-5.5889	Sensitive
MM1S	-5.2443	Sensitive
NCI-H929	-5.1836	Sensitive
OPM-2	-4.9366	Sensitive
AMO-1	-4.8391	Intermediate
SU-DHL-4	-4.8111	Intermediate
U-266	-4.8088	Intermediate
DB	-4.7802	Intermediate
KMS-12-BM	-4.7596	Intermediate
KMM-1	-4.7198	Intermediate
OCI-Ly7	-4.7053	Intermediate
HT	-4.6502	Intermediate
LP-1	-4.5975	Resistant
KMS-11	-4.5848	Resistant
KMS-12-PE	-4.5612	Resistant
RPMI-8226	-4.4932	Resistant
RPMI-8226 LR5	-4.1343	Resistant

Table 11.1: A summary of the level of resistance for each cell line.

The data are sorted according to the observed GI_{50} value.

```
> BCell.class       <- sort.BCell.class[row.names(BCellResistanceindex),]
> BCell.class$class <- as.character(BCell.class$class)
```

The following code chunk constructs a box plot of the GI_{50} values grouped into resistant, intermediate and sensitive cell lines.

```
> pdf(file.path(figure.output, "BCellresvssensBoxplot.pdf"))
> sort.BCell.class$class <- relevel(sort.BCell.class$class,
                                    names(our.colscheme)[1])
> boxplot(sort.BCell.class$BCellResistanceindex ~
          sort.BCell.class$class,
          border = our.colscheme)
> dev.off()
```

The plot is shown in Figure 11.1.

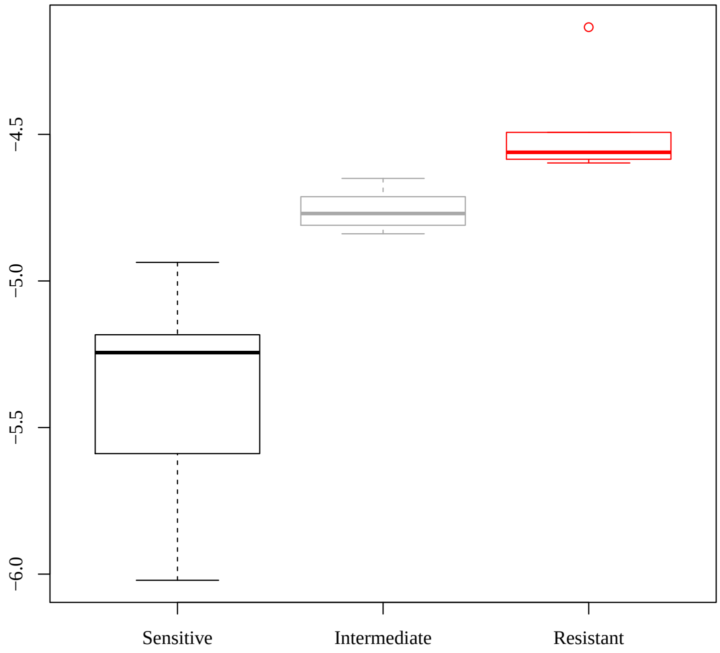

Figure 11.1: Boxplots of the GI_{50} values grouped into the resistant, intermediate and sensitive cell lines.

11.2 Cross-Validation

In order to choose optimal parameters for the LDA analysis CV is used. In the code chunk below the cutoff value of the unspecific filtering is likewise determined through CV. In this method the parameters of the LDA analysis are determined through CV for a wide range of values of the parameter `var.cutoff` in the function `nsFilter`. The combination of parameters in LDA and the `var.cutoff` that results in the maximum accuracy is chosen for further analysis.

Investigating whether the function `nsFilter` choose expression profiles that perform better than random noise, randomly selected expression profiles is used instead of `nsFilter` filter.

The various fractions to be filtered out is determined

```
> cut.offs <- seq(0.01, 0.98, by = 0.01)
```

A data frame containing the CV accuracy for LDA based on the gene expression profiles obtained through nsFilter and random selection is established.

```
> BCellLDAcrossval.res <- data.frame(cut.offs = cut.offs,
```

```
                                    accuracy = 0,
                                    acc.rand = 0)

> BCellLDAcrossval.res.file <-
      file.path(BCell.gen.dir, "BCellLDAcrossval.res.Rdata")
> if(file.exists(BCellLDAcrossval.res.file)){
      load(BCellLDAcrossval.res.file)
  }else{
      for(cut.off in cut.offs){

          print("Cut off:")
          print(cut.off)

          cell.res <-
              data.frame(nsFilter = rep(0, length(BCell.class$BCellResistanceindex),
                         random   = rep(0, length(BCell.class$BCellResistanceindex))))

          ## nsFilter expression

          BCell.filtered <- nsFilter(GEPBCell133a[, rownames(BCell.class)],
                                     var.cutoff = cut.off)$eset

          exprs(BCell.filtered) <-
              t(scale(t(exprs(BCell.filtered))))

          ## Random expressions

          n.feat       <- length(featureNames(BCell.filtered))
          rand.probes  <- sample(length(featureNames(GEPBCell133a)), n.feat)
          BCell.random <- GEPBCell133a[rand.probes, rownames(BCell.class)]

          exprs(BCell.random) <- t(scale(t(exprs(BCell.random))))

          ## Analysis with random expressions

          for(cell in 1:length(BCell.class$BCellResistanceindex)){

              print(rownames(BCell.class)[cell])
              print(cell)

              ## Leave-one-out from arrays

              looBCell.filtered <-
                  BCell.random[ ,-cell]
```

```
    ## Leave-one-out from factor levels

    fBCell      <- factor(as.character(BCell.class$class[ - cell]))
    designBCell <- model.matrix(~ 0 + fBCell)

    colnames(designBCell) <- levels(fBCell)

    contrast.matrixBCell  <-
        makeContrasts(Resistant    - Sensitive,
                      Intermediate - Sensitive,
                      levels       = designBCell)
    ## Linear model fit

    fitBCell <- lmFit(looBCell.filtered,
                      design = designBCell)

    fit2BCell <- contrasts.fit(fitBCell, contrast.matrixBCell)
    fit2BCell <- eBayes(fit2BCell)

    BCelllmFit <- topTable(fit2BCell,
                           coef = 1:(length(
                                     unique(BCell.class$class)) - 1),
                           adjust = "BH", number = Inf)

    probesets.BCell <- BCelllmFit$ID[BCelllmFit$P.Val <= pval]

    BCell1.filtered <-
        looBCell.filtered[probesets.BCell,]

    fitLDABCell <-
        sda(t(exprs(BCell1.filtered)), BCell.class$class[ - cell])

    ## Internal prediction of the cell lines

    predi <-
        predict(fitLDABCell,
                t(exprs(BCell.random[probesets.BCell, cell])))

    ## Correctly classified

    cell.res$random[cell] <-
        as.numeric(predi$class == BCell.class$class[cell])
}
```

```
## nsFilter analysis

for(cell in 1:length(BCell.class$BCellResistanceindex)){

    print(rownames(BCell.class)[cell])
    print(cell)

    ## Leave-one-out from arrays

    looBCell.filtered <-
        BCell.filtered[, -cell]

    ## Leave-one-out from factor levels

    fBCell <- factor(as.character(BCell.class$class[-cell]))

    designBCell <- model.matrix(~ 0 + fBCell)

    colnames(designBCell) <- levels(fBCell)

    contrast.matrixBCell  <-
        makeContrasts(Resistant    - Sensitive,
                      Intermediate - Sensitive,
                      levels       = designBCell)
    ## Linear model fit

    fitBCell   <- lmFit(looBCell.filtered,
                      design = designBCell)

    fit2BCell  <- contrasts.fit(fitBCell, contrast.matrixBCell)
    fit2BCell  <- eBayes(fit2BCell)

    BCelllmFit <- topTable(fit2BCell,
                           coef = 1:(length(
                                     unique(BCell.class$class))-1),
                           adjust = "BH", number = Inf)

    probesets.BCell <- BCelllmFit$ID[BCelllmFit$P.Val <= pval]

    BCell1.filtered <-
        looBCell.filtered[probesets.BCell,]

    fitLDABCell <-
```

```
            sda(t(exprs(BCell1.filtered)), BCell.class$class[-cell])

        ## Internal prediction of the cell lines

        predi <-
            predict(fitLDABCell,
                    t(exprs(BCell.filtered[probesets.BCell, cell])))

        ## Correctly classified

        cell.res$nsFilter[cell] <-
            as.numeric(predi$class == BCell.class$class[cell])
    }

    BCellLDAcrossval.res[BCellLDAcrossval.res$cut.offs ==
                         cut.off, ]$accuracy <- mean(cell.res$nsFilter)
    BCellLDAcrossval.res[BCellLDAcrossval.res$cut.offs ==
                         cut.off, ]$acc.rand <- mean(cell.res$rand)


  }
  save(BCellLDAcrossval.res, file = BCellLDAcrossval.res.file)
}
>
```

The combination of the parameters resulting in the maximum accuracy in the CV routine is stored in the object accuracy.max,

```
> accuracy.max <-
    BCellLDAcrossval.res[BCellLDAcrossval.res$accuracy ==
                         max(BCellLDAcrossval.res$accuracy), ]
```

and plotted

```
> pdf(file.path(figure.output, "BCellLDAcomparison.pdf"))
> plot(BCellLDAcrossval.res$cut.offs,
       BCellLDAcrossval.res$accuracy,
       ylim = c(min(BCellLDAcrossval.res$accuracy,
                    BCellLDAcrossval.res$acc.rand),
                max(BCellLDAcrossval.res$accuracy,
                    BCellLDAcrossval.res$acc.rand)),
       ylab = "Accuracy",
       xlab = "Fraction filtered out",type="n")
> lines(BCellLDAcrossval.res$cut.offs,
        BCellLDAcrossval.res$accuracy,
```

```
        lty = 1, col = "black")
> lines(BCellLDAcrossval.res$cut.offs,
        BCellLDAcrossval.res$acc.rand,
        lty = 2, col = "black")
> legend("bottomleft", lty = c(1, 2), lwd = c(1, 1),
         legend = c("nsFiltered probes",
                    "Random probes"),
         col = "black", bty = "n")
> dev.off()
```

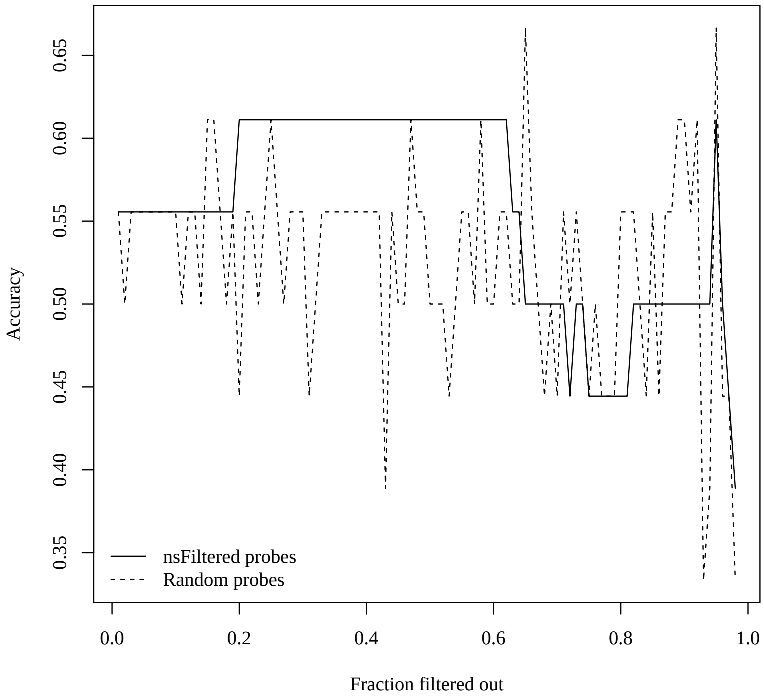

Figure 11.2: The CV accuracy for the LDA analysis at various values of the parameter var.cutoff in nsFilter. The maximum accuracy 0.611 is achieved when var.cutoff is set equal to 0.95.

11.3 Establishing the LDA Classifier

The value of the parameter `var.cutoff` resulting in the maximum accuracy is used in the function `nsFilter` on the entire dataset,

```
> cut.off <- accuracy.max$cut.offs[length(accuracy.max$cut.offs)]
> BCell.filtered <- nsFilter(GEPBCell133a[, rownames(BCell.class)],
                             var.cutoff = cut.off)$eset
```

and the gene expression profiles are scaled to have unit variance and zero mean.

```
> exprs(BCell.filtered) <-
      t(scale(t(exprs(BCell.filtered))))
```

The design matrix which is used in the function `lmFit` to extract the gene expression profiles which are significantly differentially expressed between the three groups is established.

```
> fBCell <- factor(BCell.class$class)
> designBCell <- model.matrix(~ 0 + fBCell)
> colnames(designBCell) <- levels(fBCell)
```

A matrix defining the set of contrasts is established through the function `makeContrasts` from the `limma` package.

```
> contrast.matrixBCell <-
      makeContrasts(Resistant    - Sensitive,
                    Intermediate - Sensitive,
                    levels       = designBCell)
```

Finally, a linear model is fitted.

```
> fitBCell <- lmFit(BCell.filtered, design = designBCell)
```

The coefficients and standard errors are estimated according to the constructed set of contrasts.

```
> fit2BCell <- contrasts.fit(fitBCell, contrast.matrixBCell)
```

Using the functions `eBayes` and `topTable` the gene expression profiles are ranked according to the adjusted P-values for differential expressions between the three categories of resistance to melphalan.

```
> fit2BCell  <- eBayes(fit2BCell)
> BCelllmFit <- topTable(fit2BCell,
                         coef   = 1:(length(unique(BCell.class$class)) - 1),
                         adjust = "BH",
                         number = Inf)
```

The gene expressions expressed significantly different at a significance level of 0.05 are selected for further analysis.

```
> probesets.BCell <- BCelllmFit$ID[BCelllmFit$P.Val <= pval]
> n.probes        <- length(probesets.BCell)
```

This results in 159 gene expressions. The gene symbols are looked up and stored together with the topTable result.

```
> BCellLDAgenes <-
     as.vector(unlist(lookUp(probesets.BCell, "hgu133plus2", "SYMBOL")))
> probesets.LDA.BCell            <- matrix(NA, nrow = n.probes, ncol = 5)
> row.names(probesets.LDA.BCell) <- probesets.BCell
> probesets.LDA.BCell[,2:5]      <- signif(as.matrix(BCelllmFit[1:n.probes, 4:7]), 4)
> colnames(probesets.LDA.BCell)  <- c("Symbol",colnames(BCelllmFit[, 4:7]))
> probesets.LDA.BCell[,1]        <- BCellLDAgenes
```

The matrix is shown in Table 11.2

Finally, the LDA analysis is conducted using the function sda from the package sda and saved for later predictions.

```
> fitLDABCell <-
    sda(t(exprs(BCell.filtered[probesets.BCell,])), BCell.class$class)
> save(fitLDABCell, file = file.path(BCell.gen.dir, "fitLDABCell.Rdata"))
```

Table 11.2: A summary of the toptable for the 159 gene expressions used in the LDA classifier.

Probesets	Symbol	AveExpr	F	P.Value	adj.P.Val
209310_s_at	CASP4	2.282e-16	6.343	0.001759	0.1572
221004_s_at	ITM2C	-2.722e-16	6.245	0.00194	0.1572
221912_s_at	CCDC28B	-1.258e-16	6.139	0.002158	0.1572
209735_at	ABCG2	-8.481e-17	5.973	0.002545	0.1572
221297_at	GPRC5D	2.564e-17	5.863	0.002843	0.1572
206060_s_at	PTPN22	1.627e-16	5.853	0.002872	0.1572
210145_at	PLA2G4A	-1.388e-17	5.676	0.003427	0.1572
218330_s_at	NAV2	-1.496e-16	5.673	0.003438	0.1572
209568_s_at	RGL1	-2.619e-16	5.652	0.003512	0.1572
212543_at	AIM1	1.103e-16	5.51	0.004046	0.1572
200602_at	APP	5.089e-17	5.246	0.005266	0.1572
221268_s_at	SGPP1	1.588e-16	5.236	0.00532	0.1572
205807_s_at	TUFT1	-1.82e-16	5.179	0.005635	0.1572
203758_at	CTSO	6.014e-17	5.131	0.005908	0.1572
204552_at	INPP4A	2.806e-16	5.117	0.005996	0.1572
201125_s_at	ITGB5	-4.715e-16	5.089	0.006163	0.1572
210942_s_at	ST3GAL6	-5.204e-17	5.081	0.006216	0.1572
206121_at	AMPD1	-7.859e-17	5.053	0.006393	0.1572
222258_s_at	SH3BP4	-1.218e-16	5.032	0.006529	0.1572
219572_at	CADPS2	-3.547e-17	4.992	0.006795	0.1572
211368_s_at	CASP1	4.163e-17	4.929	0.007237	0.1572
34210_at	CD52	-5.551e-17	4.916	0.007325	0.1572
202551_s_at	CRIM1	-1.349e-16	4.91	0.007374	0.1572

Table 11.2: *(continued)*

Probesets	Symbol	AveExpr	F	P.Value	adj.P.Val
202947_s_at	GYPC	-1.588e-16	4.866	0.007705	0.1572
212886_at	CCDC69	3.915e-16	4.857	0.007771	0.1572
205016_at	TGFA	-6.818e-17	4.85	0.007827	0.1572
209163_at	CYB561	4.795e-18	4.827	0.008014	0.1572
219191_s_at	BIN2	1.11e-16	4.81	0.008145	0.1572
210279_at	GPR18	-1.388e-16	4.801	0.008222	0.1572
218793_s_at	SCML1	1.295e-16	4.77	0.008479	0.1572
205110_s_at	FGF13	2.467e-17	4.755	0.008606	0.1572
214023_x_at	TUBB2B	-1.068e-16	4.646	0.009601	0.1572
202609_at	EPS8	-2.66e-17	4.643	0.009632	0.1572
221526_x_at	PARD3	1.665e-16	4.634	0.009717	0.1572
221645_s_at	ZNF83	7.922e-17	4.612	0.009929	0.1572
221704_s_at	VPS37B	-2.321e-16	4.585	0.0102	0.1572
219221_at	ZBTB38	3.296e-16	4.567	0.01039	0.1572
212715_s_at	MICAL3	1.759e-16	4.555	0.01051	0.1572
213415_at	CLIC2	7.71e-17	4.534	0.01073	0.1572
222317_at	PDE3B	5.474e-17	4.52	0.01088	0.1572
206698_at	XK	-1.48e-16	4.508	0.01102	0.1572
202732_at	PKIG	-2.313e-18	4.485	0.01128	0.1572
200660_at	S100A11	2.012e-16	4.463	0.01153	0.1572
218723_s_at	C13orf15	-7.556e-17	4.444	0.01175	0.1572
204730_at	RIMS3	-2.506e-16	4.44	0.0118	0.1572
212588_at	PTPRC	-9.252e-17	4.381	0.01251	0.1572
204589_at	NUAK1	-1.82e-16	4.379	0.01254	0.1572
207826_s_at	ID3	-3.77e-16	4.369	0.01266	0.1572
212195_at	IL6ST	1.261e-16	4.364	0.01273	0.1572
222150_s_at	PION	-9.714e-17	4.363	0.01273	0.1572
218080_x_at	FAF1	5.158e-16	4.352	0.01288	0.1572
212724_at	RND3	-1.542e-16	4.34	0.01303	0.1572
203932_at	HLA-DMB	-2.105e-16	4.332	0.01314	0.1572
214890_s_at	FAM149A	-2.393e-16	4.276	0.0139	0.1612
201301_s_at	ANXA4	-3.847e-16	4.256	0.01418	0.1612
213325_at	PVRL3	-2.409e-16	4.228	0.01458	0.1612
219159_s_at	SLAMF7	-8.635e-17	4.22	0.0147	0.1612
221727_at	SUB1	2.799e-16	4.204	0.01494	0.1612
209829_at	FAM65B	9.56e-17	4.179	0.01531	0.1612
202371_at	TCEAL4	-2.066e-16	4.177	0.01535	0.1612
221942_s_at	GUCY1A3	1.581e-16	4.165	0.01553	0.1612
201998_at	ST6GAL1	-2.891e-17	4.14	0.01592	0.1612
202746_at	ITM2A	3.084e-18	4.134	0.01602	0.1612

Table 11.2: *(continued)*

Probesets	Symbol	AveExpr	F	P.Value	adj.P.Val
207307_at	HTR2C	1.673e-16	4.104	0.01651	0.1635
201063_at	RCN1	5.86e-17	4.041	0.01758	0.1677
204613_at	PLCG2	4.24e-18	4.024	0.01789	0.1677
214608_s_at	EYA1	-3.392e-17	4.019	0.01798	0.1677
202096_s_at	TSPO	-4.572e-16	3.979	0.01871	0.1677
203795_s_at	BCL7A	-1.542e-16	3.955	0.01916	0.1677
212192_at	KCTD12	-1.295e-16	3.948	0.01929	0.1677
213502_x_at	LOC91316	1.465e-16	3.938	0.01948	0.1677
202177_at	GAS6	-2.853e-17	3.938	0.01949	0.1677
209348_s_at	MAF	-4.626e-17	3.935	0.01955	0.1677
218409_s_at	DNAJC1	2.467e-17	3.929	0.01966	0.1677
205945_at	IL6R	-2.082e-16	3.92	0.01984	0.1677
200706_s_at	LITAF	-4.179e-16	3.883	0.02058	0.1685
212442_s_at	LASS6	1.727e-16	3.882	0.02061	0.1685
213245_at	ADCY1	-1.295e-16	3.876	0.02073	0.1685
201681_s_at	DLG5	1.798e-16	3.828	0.02176	0.1709
201212_at	LGMN	-1.557e-16	3.785	0.02271	0.1709
201841_s_at	HSPB1	-4.125e-16	3.765	0.02316	0.1709
202933_s_at	YES1	1.773e-16	3.751	0.0235	0.1709
206641_at	TNFRSF17	2.603e-16	3.745	0.02364	0.1709
203986_at	STBD1	-1.226e-16	3.741	0.02373	0.1709
208892_s_at	DUSP6	-7.864e-17	3.731	0.02396	0.1709
200824_at	GSTP1	-1.11e-16	3.712	0.02443	0.1709
205229_s_at	COCH	1.382e-16	3.685	0.02509	0.1709
219696_at	DENND1B	-3.369e-16	3.681	0.0252	0.1709
217995_at	SQRDL	-1.661e-16	3.678	0.02526	0.1709
209198_s_at	SYT11	1.885e-16	3.671	0.02546	0.1709
205718_at	ITGB7	-3.115e-16	3.661	0.02571	0.1709
203476_at	TPBG	5.86e-17	3.659	0.02575	0.1709
204960_at	PTPRCAP	2.205e-16	3.657	0.0258	0.1709
203411_s_at	LMNA	2.209e-16	3.649	0.02601	0.1709
200839_s_at	CTSB	1.577e-16	3.625	0.02665	0.1709
209340_at	UAP1	-3.3e-16	3.624	0.02666	0.1709
218404_at	SNX10	-1.847e-16	3.621	0.02677	0.1709
219010_at	C1orf106	-3.481e-16	3.615	0.02691	0.1709
219551_at	EAF2	5.196e-16	3.612	0.027	0.1709
205943_at	TDO2	-1.279e-16	3.61	0.02705	0.1709
212096_s_at	MTUS1	1.789e-16	3.594	0.02748	0.1709
207039_at	CDKN2A	-2.101e-17	3.594	0.02749	0.1709
204254_s_at	VDR	-2.39e-17	3.565	0.02829	0.1741

Table 11.2: *(continued)*

Probesets	Symbol	AveExpr	F	P.Value	adj.P.Val
202242_at	TSPAN7	2.136e-16	3.543	0.02892	0.1763
202136_at	ZMYND11	-2.375e-16	3.522	0.02955	0.1764
218847_at	IGF2BP2	1.342e-16	3.52	0.02961	0.1764
212843_at	NCAM1	-3.286e-17	3.499	0.03021	0.1764
218718_at	PDGFC	-9.252e-18	3.495	0.03035	0.1764
201647_s_at	SCARB2	1.519e-16	3.48	0.03079	0.1764
200697_at	HK1	3.84e-16	3.463	0.03134	0.1764
209619_at	CD74	-1.058e-16	3.458	0.0315	0.1764
203397_s_at	GALNT3	-8.481e-17	3.457	0.03152	0.1764
201828_x_at	FAM127A	-5.089e-16	3.451	0.03172	0.1764
60474_at	FERMT1	-1.164e-16	3.441	0.03202	0.1764
221122_at	HRASLS2	2.567e-16	3.433	0.03229	0.1764
204688_at	SGCE	-3.701e-16	3.431	0.03236	0.1764
212097_at	CAV1	-7.71e-18	3.41	0.03304	0.1764
217967_s_at	FAM129A	1.664e-16	3.41	0.03306	0.1764
202946_s_at	BTBD3	-7.633e-17	3.407	0.03314	0.1764
206632_s_at	APOBEC3B	-2.018e-16	3.393	0.03362	0.1764
219003_s_at	MANEA	-3.192e-16	3.367	0.03448	0.1764
205903_s_at	KCNN3	-3.3e-16	3.366	0.03452	0.1764
206700_s_at	KDM5D	1.419e-16	3.363	0.03464	0.1764
218974_at	SOBP	-2.213e-16	3.359	0.03478	0.1764
206609_at	MAGEC1	1.288e-16	3.359	0.03479	0.1764
205297_s_at	CD79B	-1.766e-16	3.317	0.03625	0.1802
205933_at	SETBP1	1.195e-17	3.314	0.03635	0.1802
202388_at	RGS2	-2.22e-16	3.31	0.03652	0.1802
201462_at	SCRN1	-4.163e-17	3.293	0.03716	0.1802
203167_at	TIMP2	4.318e-17	3.284	0.03747	0.1802
205098_at	CCR1	-9.252e-17	3.277	0.03772	0.1802
219014_at	PLAC8	1.48e-16	3.272	0.03792	0.1802
214452_at	BCAT1	-1.593e-16	3.271	0.03795	0.1802
202017_at	EPHX1	-1.218e-16	3.268	0.03808	0.1802
210473_s_at	GPR125	-1.511e-16	3.247	0.0389	0.1827
204409_s_at	EIF1AY	1.234e-17	3.233	0.03944	0.183
220603_s_at	MCTP2	6.63e-17	3.225	0.03975	0.183
212345_s_at	CREB3L2	-2.831e-16	3.208	0.04044	0.183
203710_at	ITPR1	-5.551e-17	3.208	0.04045	0.183
200999_s_at	CKAP4	-1.82e-16	3.203	0.04066	0.183
204923_at	SASH3	-1.542e-17	3.202	0.04069	0.183
204364_s_at	REEP1	-1.739e-16	3.155	0.04263	0.1894
218618_s_at	FNDC3B	3.523e-16	3.153	0.04272	0.1894

Table 11.2: *(continued)*

Probesets	Symbol	AveExpr	F	P.Value	adj.P.Val
203324_s_at	CAV2	-5.86e-17	3.143	0.04316	0.1895
211105_s_at	NFATC1	-5.86e-17	3.136	0.04346	0.1895
220306_at	FAM46C	-4.042e-16	3.132	0.04364	0.1895
213135_at	TIAM1	9.213e-17	3.118	0.04424	0.1896
206624_at	USP9Y	2.56e-16	3.116	0.04432	0.1896
211373_s_at	PSEN2	9.059e-17	3.106	0.04478	0.1896
206167_s_at	ARHGAP6	-3.547e-17	3.096	0.04522	0.1896
201858_s_at	SRGN	6.091e-17	3.095	0.04529	0.1896
201540_at	FHL1	-1.735e-16	3.088	0.04557	0.1896
218450_at	HEBP1	-1.156e-16	3.085	0.04575	0.1896
213913_s_at	TBC1D30	-1.372e-16	3.067	0.04654	0.1916
217551_at	OR7E14P	-1.11e-16	3.039	0.04789	0.1946
221666_s_at	PYCARD	5.705e-17	3.039	0.04789	0.1946
214131_at	CYorf15B	-6.168e-17	3.032	0.04822	0.1947
219812_at	PVRIG	1.372e-16	3.005	0.04954	0.1983
213478_at	KAZ	2.282e-16	2.999	0.04985	0.1983

12 Testing the *BCell* LDA Classifier on the Arkansas Data

12.1 Predicting the Melphalan Resistance Classes

Firstly, the expression data are extracted from the expression set.

```
> Arkansas.matrix <- exprs(GEPArkansas)
```

Next, the expression set is ordered according to the LDA classifier

```
> sortBCellArkansas <- Arkansas.matrix[probesets.BCell, ]
```

and scaled so that each gene expression profile has unit variance and zero mean.

```
> sortBCellArkansas <-  t(scale(t(sortBCellArkansas)))
```

Finally, the resistance classes are predicted.

```
> predictDLDAArkansas <-
     predict(fitLDABCell, t(sortBCellArkansas))
```

The predictions are summarized in Table 12.1.

Category	Number
Intermediate	336
Resistant	119
Sensitive	104

Table 12.1: A summary of the predicted resistance classes made by the *BCell* LDA classifier.

12.2 Association between the Resistance Classes and OS

In the following code chunk Kaplan-Meier survival curves are created for the resistance classes. The result is shown in Figure 12.1.

```
> pdf(file.path(figure.output, "LDABCellArkansasKMplotOS.pdf"))
> par(mfrow = c(1, 1))
> metadataArkansas$DLDAThreshold <- predictDLDAArkansas$class
> p.BCell.LDA.OS <- PlotKM.sda(predictDLDAArkansas$class, metadataArkansas$OS,
                               ylab    = "Overall Survival",
                               col     = our.colscheme[order(names(our.colscheme))],
                               xlab    = "Time (months)",
```

```
                               legend  = names(our.colscheme),
                               col.leg = our.colscheme,
                               main    = paste("Arkansas", "\n",
                               "BCell Kaplan-Meier OS Curves",sep=""))
> dev.off()
```

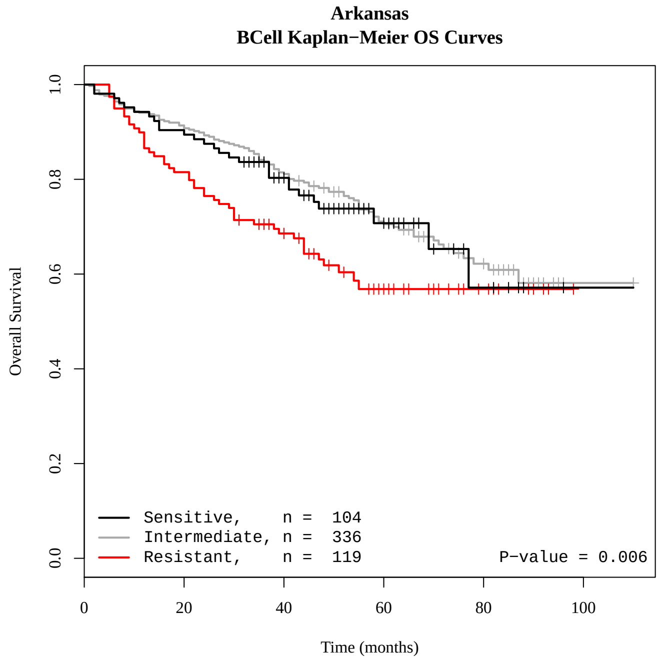

Figure 12.1: Kaplan-Meier survival curves based on the *BCell* LDA classifier. The logrank test comparing the survival curves results in a P-value of 0.006.

12.3 Association between the Resistance Classes and EFS

In the following code chunk Kaplan-Meier survival curves are created for the resistance classes. The result is shown in Figure 12.2.

```
> pdf(file.path(figure.output,"LDABCellArkansasKMplot.pdf"))
> par(mfrow = c(1, 1))
> metadataArkansas$DLDAThreshold <- predictDLDAArkansas$class
> p.BCell.LDA.EFS <- PlotKM.sda(predictDLDAArkansas$class, metadataArkansas$EFS,
                               ylab    = "Event Free Survival",
                               col     = our.colscheme[order(names(our.colscheme))],
                               xlab    = "Time (months)",
                               legend  = names(our.colscheme),
                               col.leg = our.colscheme,
```

```
                                    main     = paste("Arkansas", "\n",
                                    "BCell Kaplan-Meier EFS Curves",sep=""))
> dev.off()
```

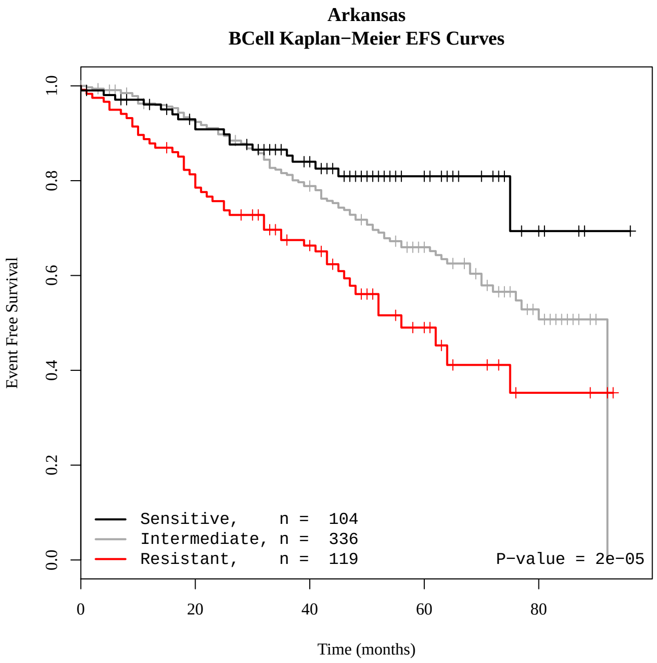

Figure 12.2: Kaplan-Meier survival curves based on the *BCell* LDA classifier. The logrank test comparing the survival curves results in a P-value of 2e-05.

13 Establishment of the *BCell* SPLS Resistance Index

13.1 Cross-Validation

When the output variable is one dimensional, SPLS has two tuning parameters: the number of hidden components K and the shrinkage parameter η. Below, the two tuning parameters are determined through CV of a wide range of values for the parameter `var.cutoff` in the function `nsFilter`. The combination of K, η and `var.cutoff` which results in the minimum Mean Square Prediction Error (MSPE) is chosen for further analysis.

By selecting the expression profiles at random instead of using nsFilter it is possible to investigate whether the function `nsFilter` choose expression profiles that perform better than random noise.

In order to avoid gene expression profiles which are not marginally related to melphalan resistance sure independence screening is used. Only gene expression profiles which are significantly correlated at the significance level specified below are used in the analysis.

```
> pval   <- 0.05
```

The P-values are not adjusted.

```
> adjust <- "none"
```

```
> set.seed(1000)
> BCell.SPLS.crossval.res.file <-
      file.path(BCell.gen.dir, "BCellSPLScrossval.res.Rdata")
> if(file.exists(BCell.SPLS.crossval.res.file)){
      load(BCell.SPLS.crossval.res.file)
  }else {
      cutoffs <- seq(0.1, 0.98, by = 0.01)

      BCell.SPLS.crossval.res <-
          data.frame(cutoffs = cutoffs,
                     eta  = 0, K=0,
                     mspe = 0, rand = 0)

      for(cutoff in cutoffs){

          BCell.filtered <- nsFilter(GEPBCell133a,
                                     var.cutoff = cutoff)$eset

          n.feat      <- length(featureNames(BCell.filtered))
```

```
        rand.probes <- sample(length(featureNames(GEPBCell133a)), n.feat)

        BCell.random        <- exprs(GEPBCell133a[rand.probes, ])

        eta <- seq(0.2, 0.99, length.out = 200)

        fit.spls.cv <-
            cv.spls(x        = t(BCell.random),
                    y        = BCellResistanceindex,
                    fold     = length(BCellResistanceindex),
                    eta      = eta,
                    K        = c(1, 2, 3),
                    plot.it  = FALSE,
                    do.SIS   = TRUE,
                    pval     = pval,
                    adjust   = adjust)

        BCell.SPLS.crossval.res[BCell.SPLS.crossval.res$cutoffs ==
                                cutoff, ]$rand <- min(fit.spls.cv$mspe)

        BCellGEP <- exprs(BCell.filtered)

        sis.cv <- SISCV(BCellGEP, BCellResistanceindex, adjust = "fdr")
        fit.spls.cv <-
            cv.spls(x        = t(BCellGEP),
                    y        = BCellResistanceindex,
                    fold     = length(BCellResistanceindex),
                    eta      = eta,
                    K        = c(1, 2, 3),
                    plot.it  = FALSE,
                    do.SIS   = TRUE,
                    pval     = pval,
                    adjust   = adjust)

        BCell.SPLS.crossval.res[BCell.SPLS.crossval.res$cutoffs ==
                                cutoff, ]$eta  <- fit.spls.cv$eta.opt
        BCell.SPLS.crossval.res[BCell.SPLS.crossval.res$cutoffs ==
                                cutoff, ]$K    <- fit.spls.cv$K.opt
        BCell.SPLS.crossval.res[BCell.SPLS.crossval.res$cutoffs ==
                                cutoff, ]$mspe <- min(fit.spls.cv$mspe)
    }
    save(BCell.SPLS.crossval.res, file = BCell.SPLS.crossval.res.file)
}
```

The result of the CV routine is extracted by the code chunk below and the optimal values for the three parameters are shown in Table 13.1.

```
> mspe.min <-  BCell.SPLS.crossval.res[BCell.SPLS.crossval.res$mspe ==
                                     min(BCell.SPLS.crossval.res$mspe), ]
> n.mspe   <- dim(mspe.min)[1]
> cutoff   <- mspe.min$cutoffs[n.mspe]
> K        <- mspe.min$K[n.mspe]
> eta      <- mspe.min$eta[n.mspe]
```

Parameter	Optimal value by CV
cutoff	0.7400
η	0.8193
K	2.0000

Table 13.1

The code chunk below constructs a plot of the minimum MSPE achieved through each of the tested values for the parameter `var.cutoff` together with minimum MSPE achieved through the randomly selected gene expressions.

```
> pdf(file.path(figure.output, "BCellMSPEcomparison.pdf"))
>   plot(BCell.SPLS.crossval.res$cutoffs,
         BCell.SPLS.crossval.res$mspe,
         ylim = c(min(BCell.SPLS.crossval.res$mspe,
                      BCell.SPLS.crossval.res$rand),
                  max(BCell.SPLS.crossval.res$mspe,
                      BCell.SPLS.crossval.res$rand)),
         ylab = "MSPE", xlab = "Fraction filtered out", type = "n",
         main = "BCell MSPE")
>   lines(BCell.SPLS.crossval.res$cutoffs,
          BCell.SPLS.crossval.res$mspe, lty = 1)
>   lines(BCell.SPLS.crossval.res$cutoffs,
          BCell.SPLS.crossval.res$rand, lty = 2)
>   legend("topleft", lty = c(1, 2),
           legend = c("nsFilter", "Random"),
           bty    = "n")
>   dev.off()
```

The plot is shown in Figure 13.1. Setting `var.cutoff` equal to 0.74 results in the mimimal MSPE.

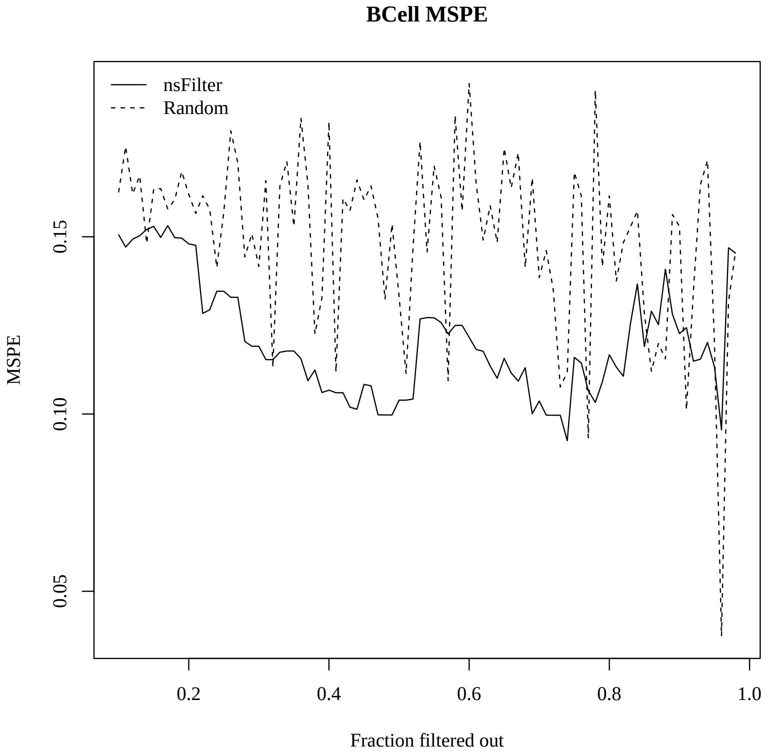

Figure 13.1: The minimum MSPE achieved through CV on K and η in SPLS for a variety of values for the parameter var.cutoff. The dashed line represents the minimum MSPE achieved through CV on K and η in SPLS for randomly selected gene expressions. The smallest minimum MSPE is obtained with a value of var.cutoff equal to 0.74

When setting `var.cutoff` equal to 0.74 the resulting CV on K and η is shown in Figure 13.2. In order to establish the plot filtering of the expression data with the parameter `var.cutoff` set equal to 0.74 is performed.

```
> BCell.filtered <- nsFilter(GEPBCell133a,
                             var.cutoff = cutoff)$eset
> GEPBCell <- exprs(BCell.filtered)
```

When using these settings in `nsFilter` only the 3297 most varying gene expression profiles are used for further analysis.

```
> BCellMSPE.file <- file.path(figure.output, "BCellMSPE.pdf")
> if(!file.exists(BCellMSPE.file)){

      fit.spls.cv <-
```

```
    cv.spls(x        = t(GEPBCell),
            y        = BCellResistanceindex,
            fold     = n.BCell,
            eta      = seq(0.5, 0.99, length.out = 1000),
            K        = c(1, 2, 3),
            plot.it  = FALSE,
            do.SIS   = TRUE,
            pval     = pval,
            adjust   = adjust)

  pdf(file.path(figure.output, "BCellMSPE.pdf"),
      width = 7, height = 7)

  trace.mspe(fit.spls.cv, header = "BCell MSPE", xlim = c(0.6, 1))

  dev.off()
}
```

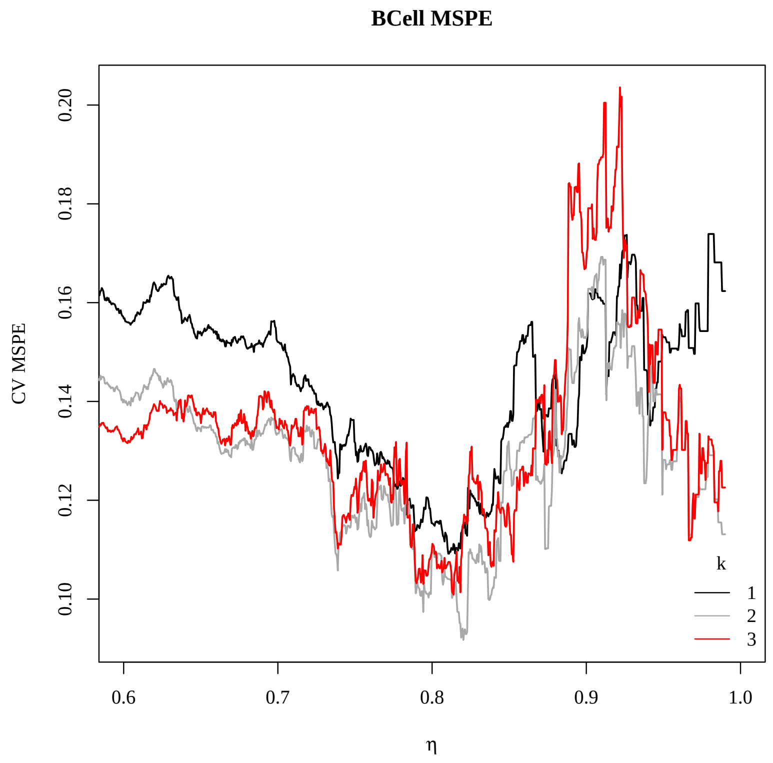

Figure 13.2: The MSPE for the SPLS regression with var.cutoff in nsFilter set equal to 0.74. This leads to the selection of K=2 hidden components and a sparsity parameter η=0.8193.

In order to investigate the accuracy of the SPLS model with the specific settings the CV predicted resistance index is calculated once more and stored in the object `pred.1`.

```
> pred.1            <- matrix(NA, nrow = n.BCell, ncol = 2)
> rownames(pred.1) <- BCell.names.sorted
> colnames(pred.1) <- c("Measured", "predicted")
> pred.1[, 1]       <- BCellResistanceindex[BCell.names.sorted, ]
> for(i in BCell.names.sorted){

      GEPBCell.cv <- exprs(BCell.filtered[, sampleNames(GEPBCell133a) %w/o% i])

      y <- BCellResistanceindex[sampleNames(GEPBCell133a) %w/o% i, ]

      BCell.fit.spls.cv <- spls(x       = t(GEPBCell.cv),
                                y       = y,
                                K       = K,
                                eta     = eta,
                                do.SIS  = TRUE,
                                pval    = pval,
                                adjust  = adjust)

      newx <- exprs(GEPBCell133a)[row.names(GEPBCell.cv), i]

      pred.1[i, 2] <- as.vector(predict(BCell.fit.spls.cv,
                                newx = t(as.matrix(newx))))

  }
```

In Figure 13.3A the CV predicted melphalan resistance is plotted against the measured GI_{50} values.

When the parameter `var.cutoff` in the function `nsFilter` is determined in this manner the resulting MSPE obtained through CV with SPLS is overoptimistic. In order to determine how well the model performs, CV over the chosen parameters including `var.cutoff` is performed.

```
> pred.2            <- matrix(NA, nrow = n.BCell, ncol = 3)
> rownames(pred.2) <- BCell.names.sorted
> colnames(pred.2) <- c("Measured", "Predicted Naive", "Predicted")
> pred.2[, 1]       <- BCellResistanceindex[BCell.names.sorted, ]
> pred.2[, 2]       <- pred.1[, 2]
> for(i in BCell.names.sorted){
      BCell.filtered.cv <-
          nsFilter(GEPBCell133a[, sampleNames(GEPBCell133a) %w/o% i],
```

```
                var.cutoff = cutoff)$eset

    GEPBCell.cv <- exprs(BCell.filtered.cv)

    y <- BCellResistanceindex[sampleNames(GEPBCell133a) %w/o% i, ]


    BCell.fit.spls.cv <- spls(x       = t(GEPBCell.cv),
                              y       = y,
                              K       = K,
                              eta     = eta,
                              do.SIS  = TRUE,
                              pval    = pval,
                              adjust  = adjust)

    newx <- exprs(GEPBCell133a)[row.names(GEPBCell.cv), i]

    pred.2[i, 3] <- as.vector(predict(BCell.fit.spls.cv,
                              newx = t(as.matrix(newx))))

}
```

In Figure 13.3B the predicted resistance index is plotted against the measured resistance index. As assumed the result is slightly worse than when the parameter `var.cutoff` is not included in the CV step.

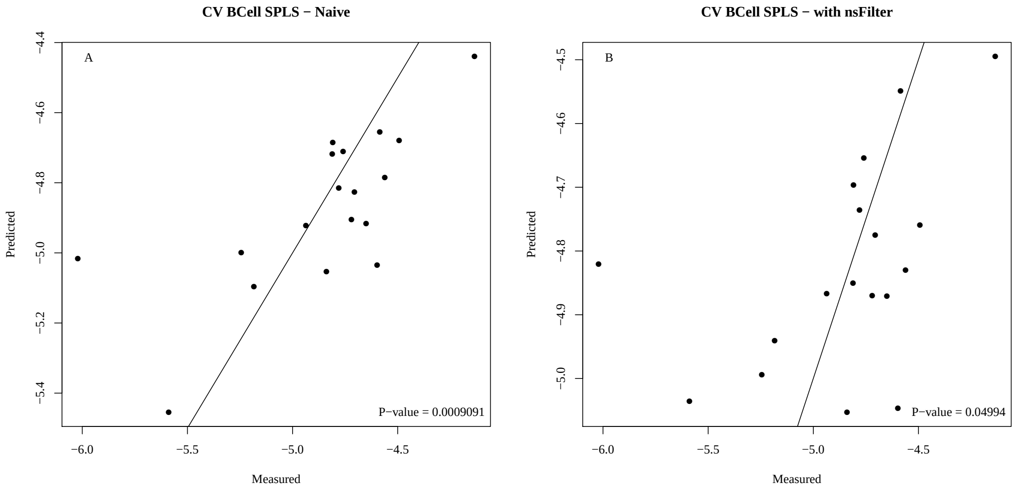

Figure 13.3: Predicted resistance index vs. the measured resistance index. The left panel shows CV after filtering. The right panel shows predictions where filtering is performed each time a cell line is left out.

13.2 Establishment of the Gene Expression Signature

In the previous section various values were determined through CV. In this section these values are used for fitting a gene expression signature. For this purpose the function `spls` from the package `spls` is used.

The gene expression signature is established and saved for later predictions.

```
> BCell.fit.spls <- spls(x      = t(GEPBCell),
                         y      = BCellResistanceindex,
                         K      = K,
                         eta    = eta,
                         do.SIS = TRUE,
                         pval   = pval,
                         adjust = adjust)
> save(BCell.fit.spls,
       file = file.path("Generated data", "BCell", "BCell.fit.spls.Rdata"))
```

13.3 Investigation of the chosen Gene Expression Profile

Only 19 gene expression profiles are used in the signature. In the code chunk below the gene symbols for the 19 gene expression profiles are looked up. Furthermore, in order to see if these gene profiles have been observed in other papers studying chemoresistance the following search is conducted on PubMed.

```
> resTable  <- lookUpPubmed(BCell.fit.spls, n.coef = 3)
```

The result of the search is shown in Table 13.2. In order to investigate how the identified probes relate to chemoresistance marginally correlations are plotted in Section 13.4.

```
> BCellCoef <- coef(BCell.fit.spls)
> BCellCoef <- BCellCoef[BCellCoef != 0, ]
> BCellCoef <- round(BCellCoef[order(BCellCoef)], 4)
> corm <- GEPBCell[names(BCellCoef), ]
> for(i in 1:length(BCellCoef)){
      pdf(paste(figure.output, "/", i, "BCellcorrelations.pdf", sep = ""),
          width = 7, height = 7)

      plot(BCellResistanceindex ~ corm[i, ],
           ylab = "Resistance index",
           xlab = "Gene expression",
           type = "n")

      title(unlist(lookUp(rownames(corm)[i],
                          "hgu133plus2", "SYMBOL")))
```

U133 ID	Gene Symbol	Mean	SD	Location	Weight	PMID
205990_s_at	WNT5A	10.719	0.719	3p21-p14	−0.065	3
203708_at	PDE4B	6.272	1.110	1p31	−0.053	2
201990_s_at	CREBL2	5.980	0.667	12p13	−0.046	0
218751_s_at	FBXW7	7.539	1.022	4q31.3	−0.044	3
201889_at	FAM3C	6.752	1.808	7q31	−0.039	0
206405_x_at	USP6	9.859	0.935	17p13	−0.038	0
219049_at	CSGALNACT1	8.712	0.867	8p21.3	−0.037	0
205862_at	GREB1	5.326	0.953	2p25.1	−0.034	2
219748_at	TREML2	6.556	2.162	6p21.1	−0.033	0
204786_s_at	IFNAR2	10.851	0.905	21q22.1, 21q22.11	−0.033	4
204204_at	SLC31A2	4.451	1.450	9q31-q32	−0.025	0
217825_s_at	UBE2J1	8.450	0.877	6q15	−0.020	0
213555_at	RWDD2A	5.133	1.933	6q14.2	−0.019	0
212122_at	RHOQ	9.410	0.813	2p21	−0.016	0
203895_at	PLCB4	7.099	2.446	20p12	−0.015	0
202043_s_at	SMS	7.162	1.033	Xp22.1	0.011	48
217104_at	ST20	6.569	1.862	15q25.1	0.012	0
212055_at	C18orf10	7.653	0.776	18q12.2	0.025	0
221210_s_at	NPL	4.953	1.076	1q25	0.032	0

Table 13.2: Location and weight for the 19 probes identified by use of the BCell panel in combination with SPLS regression.

```
    text(corm[i, ], BCellResistanceindex,
         names(unlist(lookUp(colnames(corm), "hgu133plus2",
                             "SYMBOL"))), cex = 0.5)
    legend("bottomleft",
           title = paste("     Cor =", as.character(round(
           cor(BCellResistanceindex, corm[i, ]), 2))),
           legend = "", bty = "n", cex = 0.5)
    dev.off()
}
```

13.4 Plots of Marginal Associations

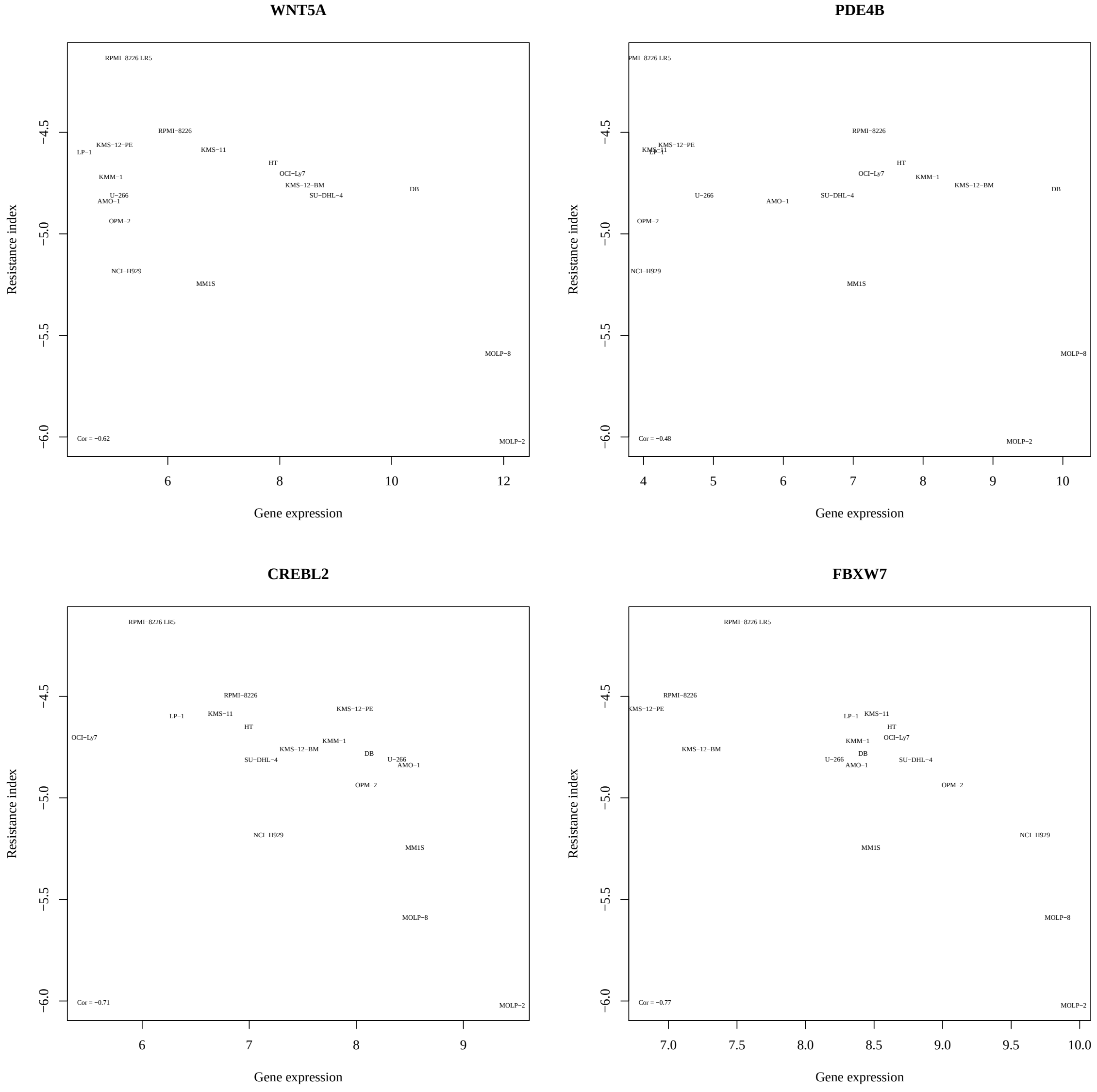

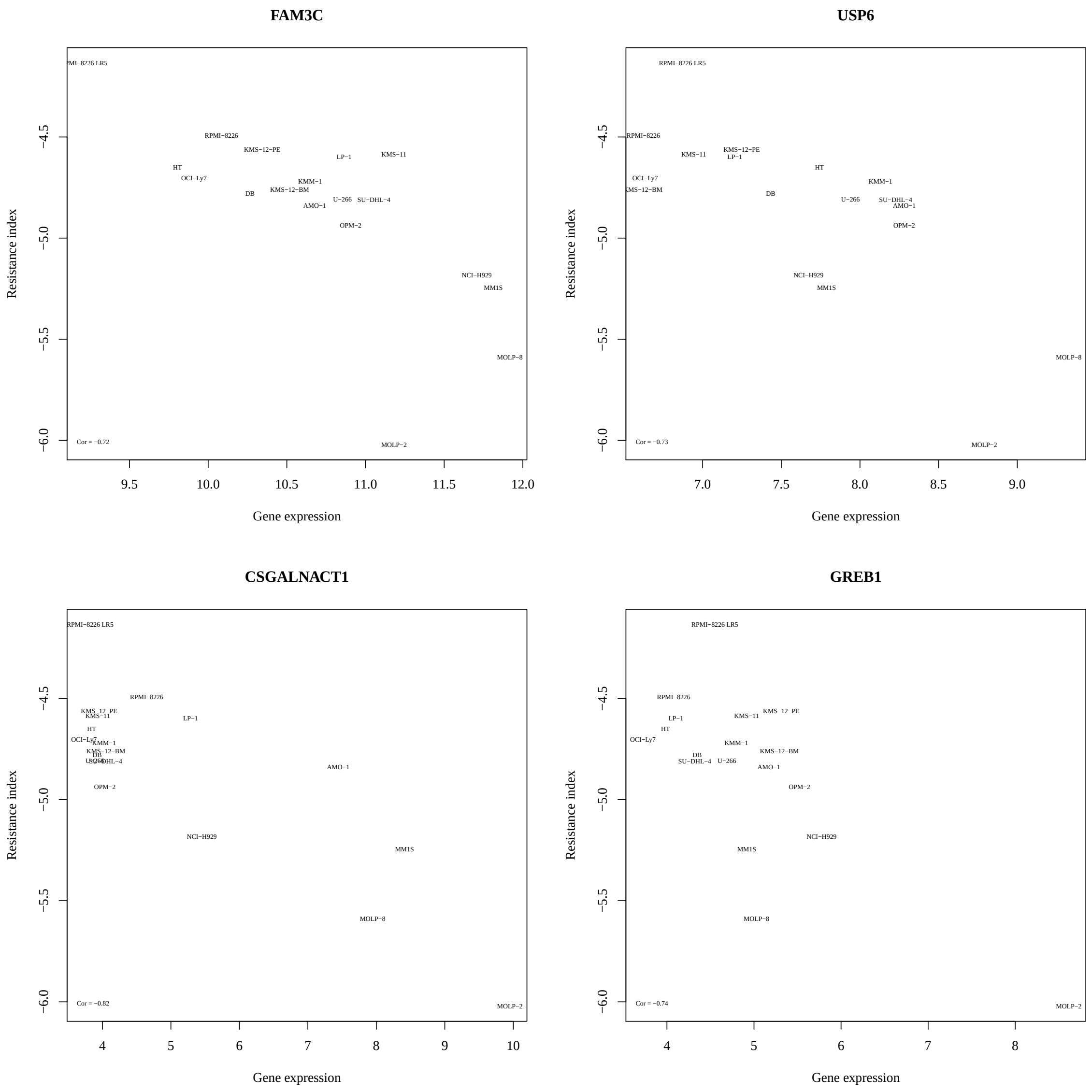
FAM3C
USP6
CSGALNACT1
GREB1
Resistance index
Gene expression
Cor = −0.72
Cor = −0.73
Cor = −0.82
Cor = −0.74
RPMI−8226 LR5
RPMI−8226
KMS−12−PE
KMS−11
LP−1
HT
OCI−Ly7
KMM−1
KMS−12−BM
DB
U−266
SU−DHL−4
AMO−1
OPM−2
NCI−H929
MM1S
MOLP−8
MOLP−2

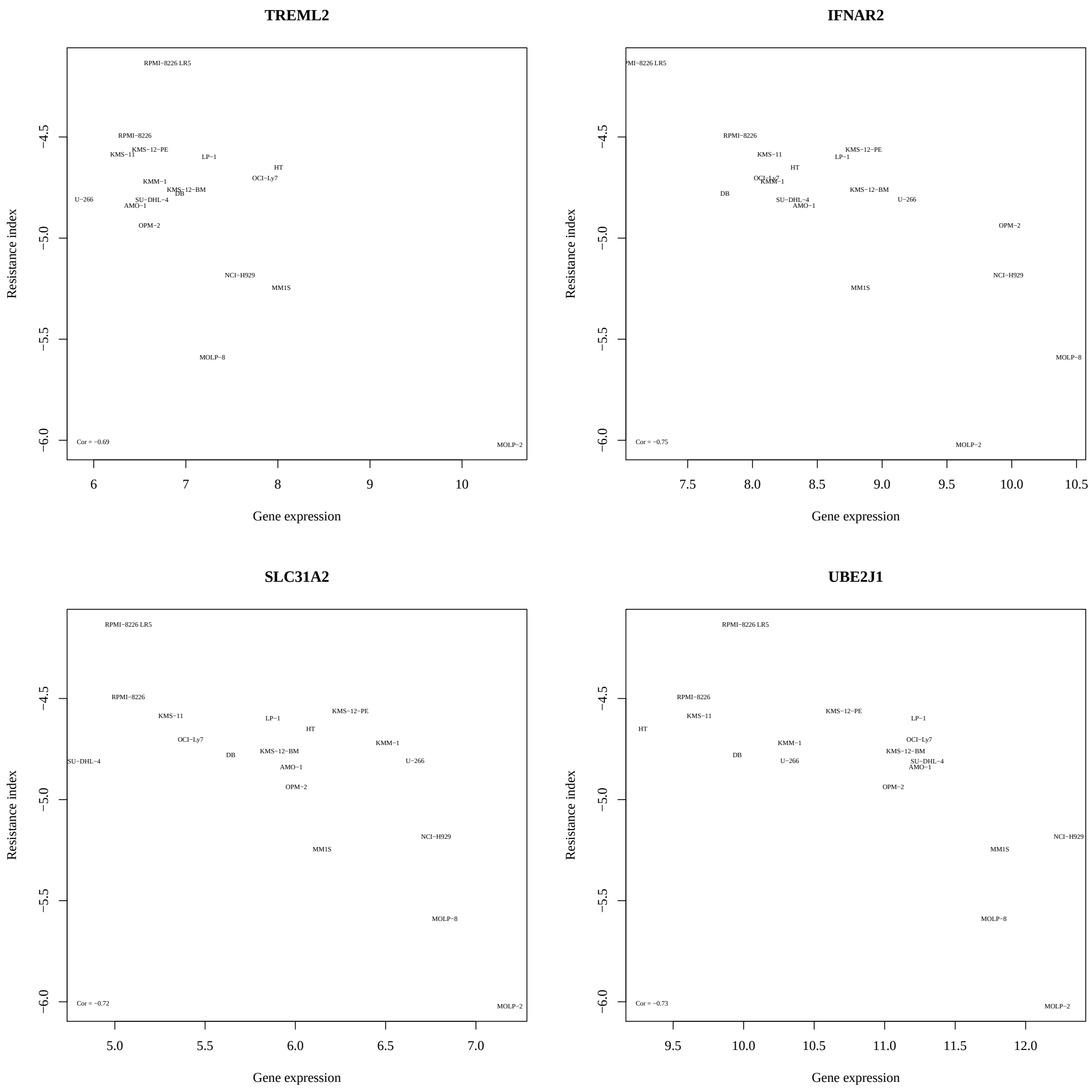
TREML2
Resistance index
Gene expression
RPMI−8226 LR5
RPMI−8226
KMS−12−PE
KMS−11
LP−1
HT
OCI−Ly7
KMM−1
KMS−12−BM
DB
U−266
SU−DHL−4
AMO−1
OPM−2
NCI−H929
MM1S
MOLP−8
Cor = −0.69
MOLP−2
IFNAR2
Resistance index
Gene expression
RPMI−8226
KMS−11
KMS−12−PE
LP−1
HT
DB
KMS−12−BM
SU−DHL−4
AMO−1
U−266
OPM−2
NCI−H929
MM1S
MOLP−8
Cor = −0.75
MOLP−2
SLC31A2
Resistance index
Gene expression
RPMI−8226 LR5
RPMI−8226
KMS−11
LP−1
KMS−12−PE
HT
OCI−Ly7
KMM−1
SU−DHL−4
DB
KMS−12−BM
U−266
AMO−1
OPM−2
NCI−H929
MM1S
MOLP−8
Cor = −0.72
MOLP−2
UBE2J1
Resistance index
Gene expression
RPMI−8226 LR5
RPMI−8226
KMS−11
KMS−12−PE
LP−1
HT
OCI−Ly7
KMM−1
DB
KMS−12−BM
U−266
SU−DHL−4
AMO−1
OPM−2
NCI−H929
MM1S
MOLP−8
Cor = −0.73
MOLP−2

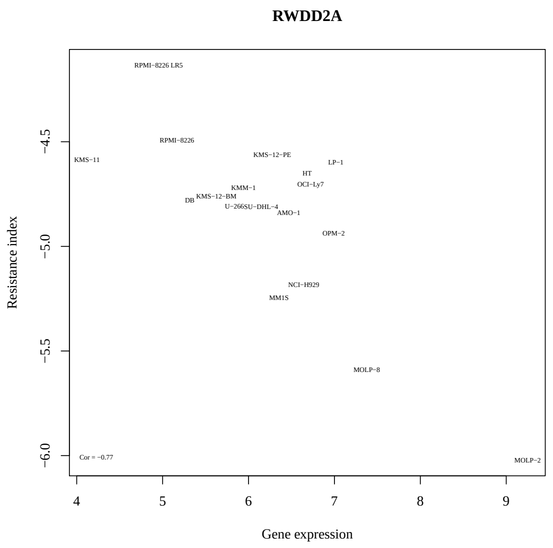
RWDD2A
RPMI−8226 LR5
RPMI−8226
KMS−11
KMS−12−PE
LP−1
HT
OCI−Ly7
KMM−1
KMS−12−BM
DB
U−266
SU−DHL−4
AMO−1
OPM−2
NCI−H929
MM1S
MOLP−8
MOLP−2
Cor = −0.77
Resistance index
Gene expression
−4.5
−5.0
−5.5
−6.0
4
5
6
7
8
9

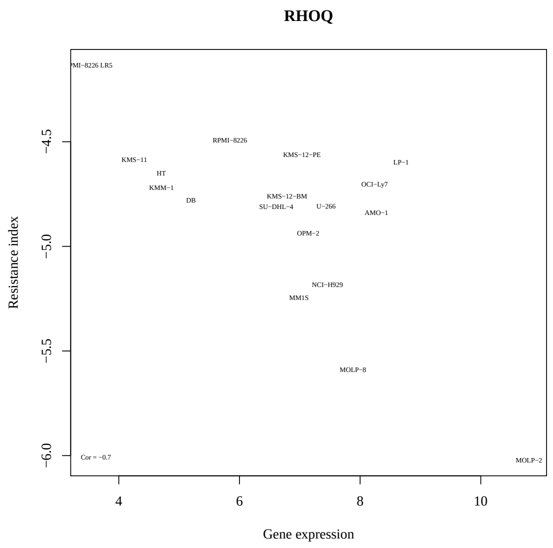
RHOQ
RPMI−8226 LR5
RPMI−8226
KMS−11
KMS−12−PE
LP−1
HT
OCI−Ly7
KMM−1
KMS−12−BM
DB
SU−DHL−4
U−266
AMO−1
OPM−2
NCI−H929
MM1S
MOLP−8
MOLP−2
Cor = −0.7
Resistance index
Gene expression
−4.5
−5.0
−5.5
−6.0
4
6
8
10

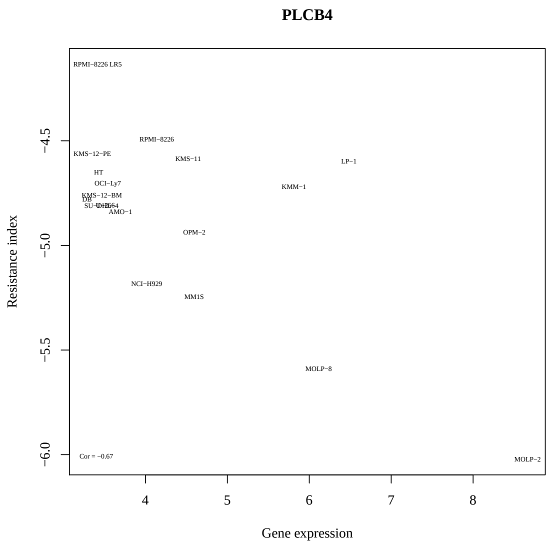
PLCB4
RPMI−8226 LR5
RPMI−8226
KMS−12−PE
KMS−11
LP−1
HT
OCI−Ly7
KMS−12−BM
KMM−1
AMO−1
OPM−2
NCI−H929
MM1S
MOLP−8
MOLP−2
Cor = −0.67
Resistance index
Gene expression
−4.5
−5.0
−5.5
−6.0
4
5
6
7
8

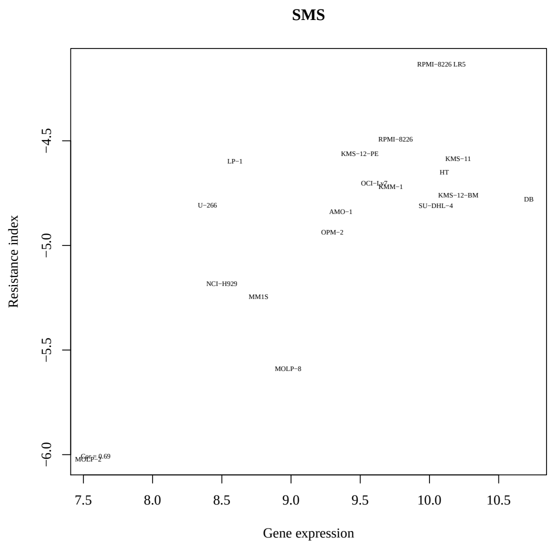
SMS
RPMI−8226 LR5
RPMI−8226
KMS−12−PE
KMS−11
LP−1
HT
OCI−Ly7
KMM−1
KMS−12−BM
DB
U−266
SU−DHL−4
AMO−1
OPM−2
NCI−H929
MM1S
MOLP−8
Cor = 0.69
Resistance index
Gene expression
−4.5
−5.0
−5.5
−6.0
7.5
8.0
8.5
9.0
9.5
10.0
10.5

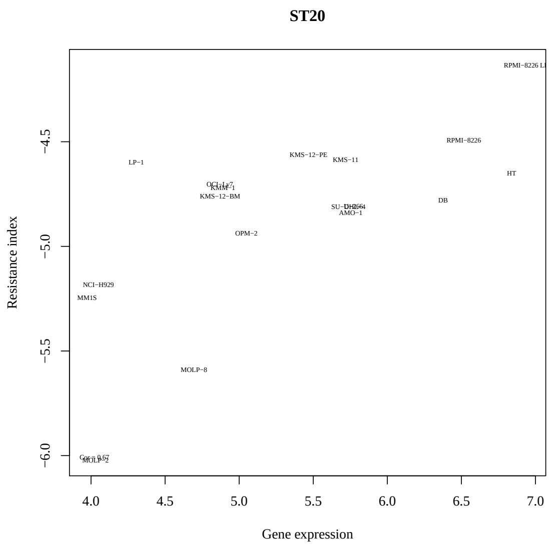
ST20
Resistance index
Gene expression
−4.5
−5.0
−5.5
−6.0
4.0
4.5
5.0
5.5
6.0
6.5
7.0

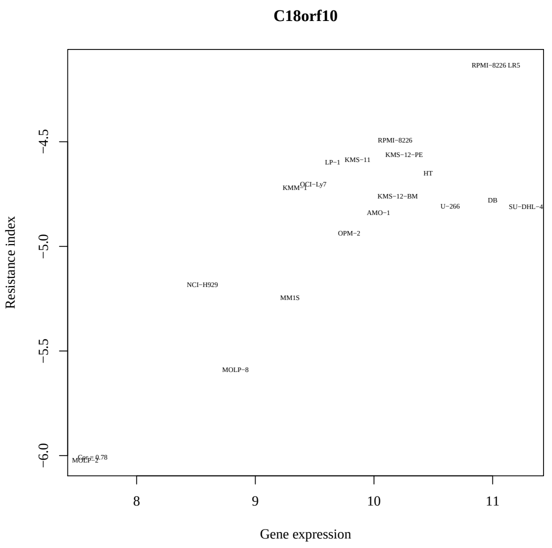
C18orf10
Resistance index
Gene expression
−4.5
−5.0
−5.5
−6.0
8
9
10
11

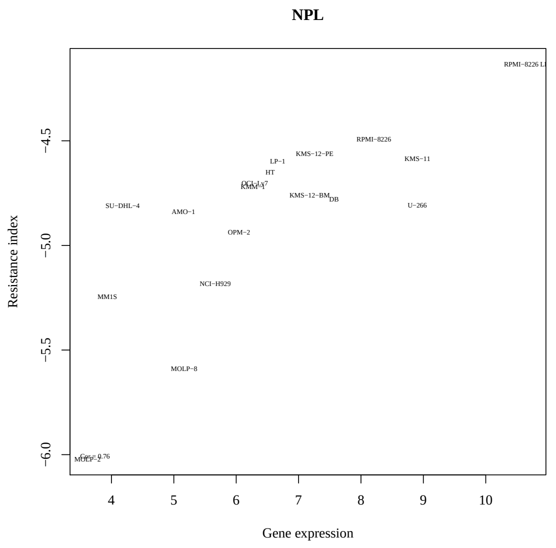
NPL
Resistance index
Gene expression
−4.5
−5.0
−5.5
−6.0
4
5
6
7
8
9
10

14 Testing the *BCell* SPLS Resistance Index

14.1 Predicting the Melphalan Resistance in the *Arkansas* data

Firstly, the expression data are extracted from the expression set.

```
> arkansas.matrix <- exprs(GEPArkansas)
```

Next, the probes which were removed by `nsFilter` with `var.cutoff` set equal to 0.74 are discarded from the matrix.

```
> BCellArkansasTest <-
    arkansas.matrix[featureNames(BCell.filtered),]
```

Finally, the signature is used to predict each patient's resistance index.

```
> BCellArkansasindex <- as.vector(predict(BCell.fit.spls,
                                           newx = t(BCellArkansasTest)))
```

14.1.1 Association between the Resistance Index and OS

Kaplan-Meier Survival Curves

Kaplan-Meier survival curves are constructed for each of the groups sensitive, intermediate and resistant. The 25% with the lowest predicted melphalan resistance index are categorized as sensitive and the 25% with the highest predicted melphalan resistance index are categorized as resistant. The remaining subjects are characterised as having intermediate resistance.

The Kaplan-Meier survival curves are plotted with the code chunk below and the result is shown in Figure 14.1.

```
> pdf(file.path(figure.output,"BCellarkansasOSKMplot.pdf"))
> BCellarkansasOSKM.P <-
      PlotKM(BCellArkansasindex, metadataArkansas$OS,
             cut.points = cut.points,
             xlab       = "Time (months)",
             ylab       = "OS ratio",
             xmax       = 110,
             main       = "Arkansas \n BCell Kaplan-Meier OS curves")
> dev.off()
```

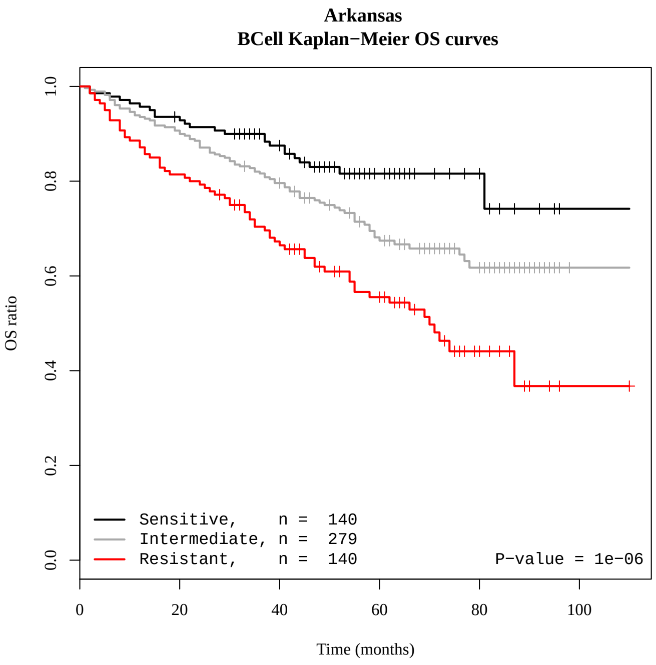

Figure 14.1: Kaplan-Meier survival curves based on the *BCell* resistance index. The logrank test comparing the survival curves results in a P-value of 1e-06.

Cox Proportional Hazards

The HR and its 95% confidence interval are calculated between the sensitive and resistant classes.

```
> HR <- CalcHR(BCellArkansasindex, metadataArkansas$OS..months,
         metadataArkansas$OS.censor == 1)
> HR
```

```
[1] "HR = 2.9 (2.41,3.35)"
```

A Cox proportional hazards model is fitted where the association between log relative hazard and the resistance index is modelled by a spline with four knots.

```
> n.knots   <- 4
> coxfit.os <- coxph(metadataArkansas$OS ~ rcs(BCellArkansasindex, n.knots))
```

Table 14.1 summarizes three tests for no association between the log relative hazard and the resistance index.
The code chunk below produces a plot of the log relative hazard as a function of the *BCell* resistance index. The result is depicted in Figure 14.2.

Test	Test	D.o.F	P-value
Likelihood ratio test	29.7	3	$1.62e-06$
Wald test	27.9	3	$3.79e-06$
Score (log rank) test	29.4	3	$1.87e-06$

Table 14.1: A summary of three tests for no association between the log relative hazard and the resistance index.

.

```
> pdf(file.path(figure.output,"BCellarkansasOSPredictors.pdf"))
> par(mfrow = c(1, 1))
> d <- datadist(BCellArkansasindex)
> options(datadist = "d", width = 150)
> BCellf <- cph(metadataArkansas$OS ~ rcs(BCellArkansasindex, n.knots))
> plot(BCellf, xlab = "Fitted BCell resistance index")
> title("Arkansas \n BCell - OS Cox Proportional Hazards")
> legend("bottomright", "", bty = "n",
         title = paste("P-value = ",
         as.character(signif(unlist(BCellf)$stats.P, 1)),
         sep = ""))
> dev.off()
```

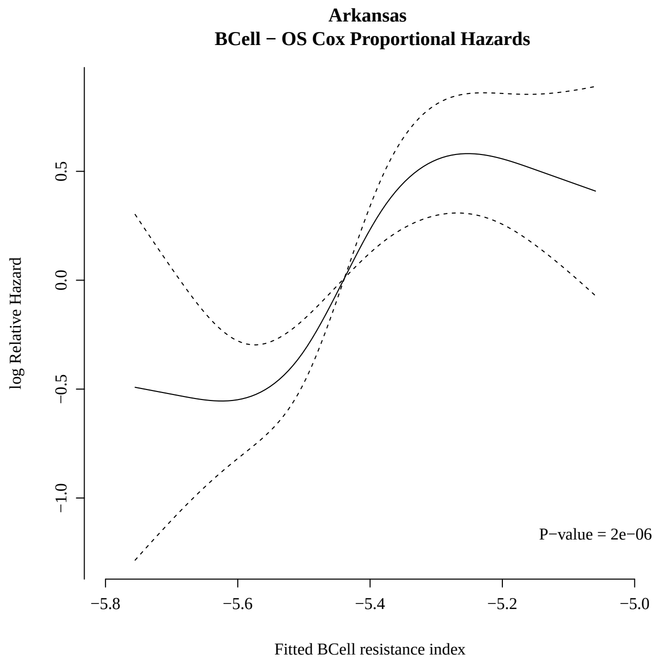

Figure 14.2: The log relative hazard as a function of the *BCell* resistance index. The P-value is the likelihood ratio test for no RCS-association between log relative hazard and resistance index. The dashed lines represent 95% confidence intervals.

14.1.2 Association between the Resistance Index and EFS

Kaplan-Meier Survival Curves

Similarly to the previous section Kaplan-Meier survival curves are made with the patients grouped to be sensitive, intermediate or resistant, however, EFS is used as endpoint instead of OS.

The Kaplan-Meier survival curves are plotted with the code chunk below and the result is shown in Figure 14.3.

```
> pdf(file.path(figure.output,"BCellarkansasEFSKMplot.pdf"))
> BCellarkansasEFSKM.P <-
      PlotKM(BCellArkansasindex,metadataArkansas$EFS,
             cut.points = cut.points,
             xlab       = "Time (months)",
             ylab       = "EFS ratio",
             xmax       = 110,
             main       = "Arkansas \n BCell Kaplan-Meier EFS curves"
             )
> dev.off()
```

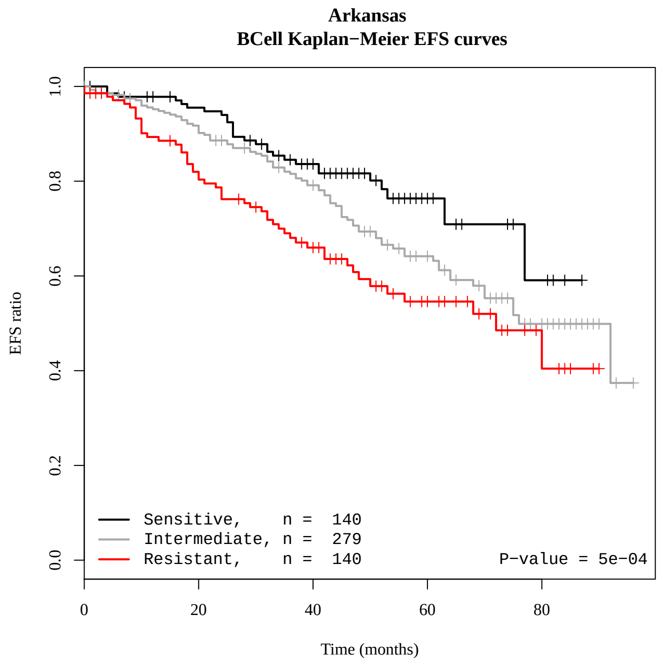

Figure 14.3: Kaplan-Meier survival curves based on the *BCell* resistance index. The logrank test comparing the survival curves results in a P-value of 5e-04.

Cox Proportional Hazards

The HR and its 95% confidence interval are calculated between the sensitive and resitant classes.

```
> HR <- CalcHR(BCellArkansasindex, metadataArkansas$EFS.months,
         metadataArkansas$EFS.censor == 1)
> HR
```

```
[1] "HR = 2.2 (1.75,2.67)"
```

A Cox proportional hazards model is fitted where the association between log relative hazard and the resistance index is modelled by a spline with four knots.

```
> n.knots   <- 4
> coxfit.os <-  coxph(metadataArkansas$EFS ~ rcs(BCellArkansasindex, n.knots))
```

Table 14.2 summarizes three tests for no association between the log relative hazard and the resistance index.
The code chunk below produces a plot of the log relative hazard as a function of the *BCell* resistance index. The result is depicted in Figure 14.4.

```
> pdf(file.path(figure.output,"BCellarkansasEFSPredictors.pdf"))
> par(mfrow=c(1,1))
```

Test	Test	D.o.F	P-value
Likelihood ratio test	17.9	3	0.000453
Wald test	17.0	3	0.000719
Score (logrank) test	17.7	3	0.000517

Table 14.2: A summary of three tests for no association between log relative hazard and the predicted resistance index.

.

```
> d <- datadist(BCellArkansasindex)
> options(datadist="d", width=150)
> BCellf <- cph(metadataArkansas$EFS ~ rcs(BCellArkansasindex, n.knots))
> plot(BCellf, xlab="Fitted BCell resistance index")
> title("Arkansas \n BCell - EFS Cox Proportional Hazards")
> legend("bottomright", "", bty = "n",
         title = paste("P-value = ",
         as.character(signif(unlist(BCellf)$stats.P, 1)),
         sep=""))
> dev.off()
```

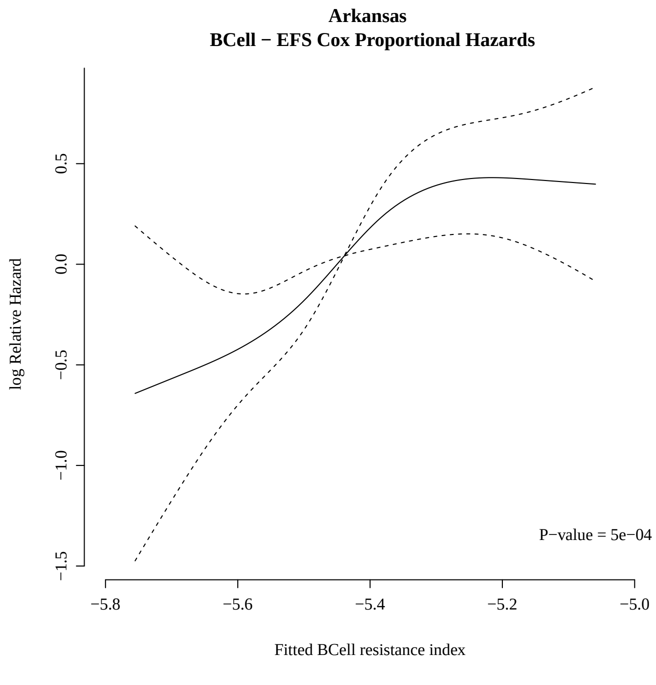

Figure 14.4: The log relative hazard as a function of the *BCell* resistance index. The P-value is the likelihood ratio test for no RCS-association between log relative hazard and resistance. The dashed lines represent 95% confidence intervals.

14.2 Predicting Melphalan Resistance in the *Hummel* Data

Firstly, the expression data are extracted from the expression set.

```
> hummel.matrix <- exprs(GEPHummel)
```

Next, the probes which were removed by `nsFilter` with `var.cutoff` set equal to 0.74 are discarded for the matrix.

```
> BCellHummelTest <-  hummel.matrix[featureNames(BCell.filtered),]
```

Finally, the signature is used to predict the patients resistance index.

```
> BCellHummelindex <- as.vector(predict(BCell.fit.spls,
                                        newx = t(BCellHummelTest)))
```

14.2.1 Association between the Resistance Index and OS

Kaplan-Meier Survival Curves

Kaplan-Meier survival curves are made with the patients grouped into sensitive, intermediate and resistant. The 25% with the lowest predicted melphalan resistance index are categorized as sensitive and the 25% with the highest predicted melphalan resistance index are categorized as resistant. The remaining subjects are characterised as having intermediate resistance.

The Kaplan-Meier survival curves are plotted with the code chunk below and the result is shown in Figure 14.5.

```
> pdf(file.path(figure.output, "BCellhummelOSKMplot.pdf"))
> BCellhummelOSKM.P <-
      PlotKM(BCellHummelindex, metadataHummel$OS,
             cut.points = cut.points,
             xlab       = "Time (months)",
             ylab       = "OS ratio",
             xmax       = 110,
             main       = "Hummel \n BCell Kaplan-Meier OS curves"
             )
> dev.off()
```

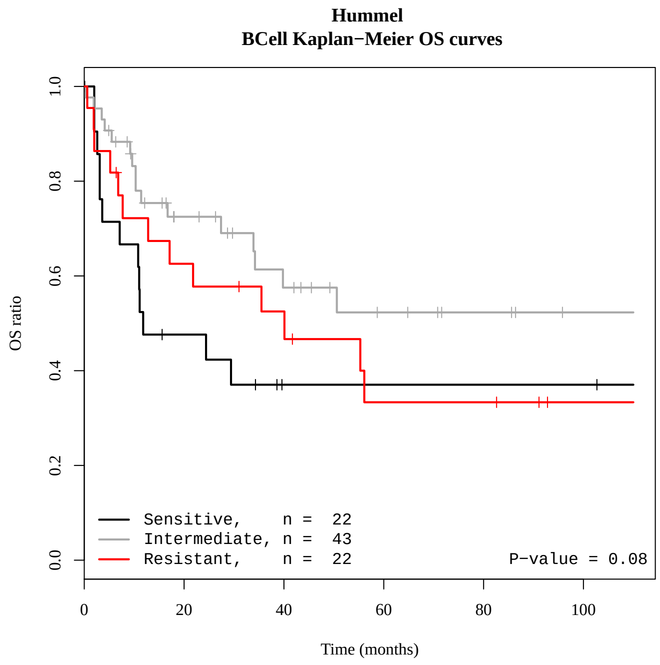

Figure 14.5: Kaplan-Meier survival curves based on the *BCell* resistance index. The logrank test comparing the survival curves results in a P-value of 0.08.

Cox Proportional Hazards

A Cox proportional hazards model is fitted where the association between log relative hazard and the resistance index is modelled by a spline with four knots.

```
> n.knots <- 4
> coxfit.os <-
    coxph(metadataHummel$OS ~ rcs(BCellHummelindex, n.knots))
```

Table 14.3 summarizes three tests for no association between log relative hazard and the predicted resistance index.

Test	Test	D.o.F	P-value
Likelihood ratio test	2.45	3	0.484
Wald test	2.81	3	0.423
Score (log rank) test	2.89	3	0.409

Table 14.3: A summary of three tests for no association between log relative hazard and the predicted resistance index.

The code chunk below produces a plot of the log relative hazard as a function of the *BCell* resistance index. The result is depicted in Figure 14.6.

```
> pdf(file.path(figure.output,"BCellhummelOSPredictors.pdf"))
> par(mfrow=c(1,1))
> d <- datadist(BCellHummelindex)
> options(datadist="d", width=150)
> BCellf <- cph(metadataHummel$OS ~ rcs(BCellHummelindex,4))
> plot(BCellf,
       xlab="Fitted BCell resistance index")
> title("Hummel \n BCell - OS Cox Proportional Hazards")
> legend("bottomright", "", bty = "n",
         title = paste("P-value = ",
         as.character(signif(unlist(BCellf)$stats.P, 1)),
         sep = ""))
> dev.off()
```

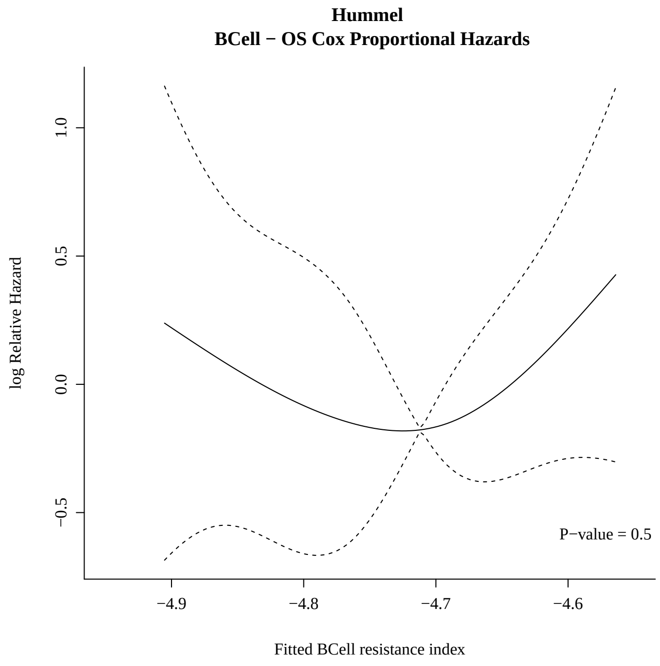

Figure 14.6: This figure shows the log relative hazard as a function of the *BCell* resistance index. The P-value is the likelihood ratio test for no RCS-association between log Relative Hazard and resistance. The dashed lines represent 95% confidence intervals.

14.3 Regularisation Paths for the BCell Panel

Set seed for repeatability

```
> set.seed(1000)
```

Settings for the repeated datasets

```
> cutoff <- cutoff
> newGEP.filtered <- nsFilter(GEPBCell133a,
                              var.cutoff = cutoff)$eset
> newGEP.matrix   <- t(scale(t(exprs(newGEP.filtered))))

> top.correlated <- 100
> n.chosen       <-  20

> genchem.list <- corFilter(exprs.matrix = newGEP.matrix,
                            chemoindex   = BCellResistanceindex,
                            alpha        = 0.85)

> n.repeats <- 100

> BCell.reg.path.res <- data.frame(sens = rep(0, n.repeats),
                                   spec = rep(0, n.repeats),
                                   fdr  = rep(0, n.repeats),
                                   eta  = rep(0, n.repeats),
                                   K    = rep(0, n.repeats))
```

Perform the repeated analyses.

```
> BCell.reg.path.res.files <- file.path(BCell.gen.dir, "BCell.reg.path.res.Rdata")
> if(file.exists(BCell.reg.path.res.files)){
      load(BCell.reg.path.res.files)
  }else{
      for(reg.n in 1:n.repeats){

          print(reg.n)

          fixed.list  <- sample(rownames(genchem.list[1 : top.correlated, ]),
                                n.chosen,replace=FALSE)
          name.list   <- rownames(newGEP.matrix)
          perm.list   <- setdiff(name.list, fixed.list)

          sample.list <-  sample(1:dim(newGEP.matrix)[2], replace=FALSE)
          perm.matrix <- rbind(newGEP.matrix[fixed.list, ],
                               newGEP.matrix[perm.list, sample.list])

          eta <- seq(0.2, 0.99, length.out = 200)
          K   <- c(1, 2, 3)
          fit.spls.cv <-
              cv.spls(x       = t(perm.matrix),
                      y       = BCellResistanceindex,
                      fold    = n.BCell, eta = eta, K = K,
```

```
            plot.it = FALSE,
            do.SIS  = TRUE,
            pval    = pval,
            adjust  = adjust)

eta.opt <- fit.spls.cv$eta.opt
K.opt   <- fit.spls.cv$K.opt

BCell.reg.path.res$eta[reg.n] <- eta.opt
BCell.reg.path.res$K[reg.n]   <- K.opt

coef.container <- matrix(0,ncol=length(eta), nrow =
                         length(perm.matrix[, 1]))
colnames(coef.container) <- rownames(fit.spls.cv$mspemat)
rownames(coef.container) <- rownames(perm.matrix)

colidx <- 1
for(i in eta){
    print(i)

curr.fit.spls <- spls(x       = t(perm.matrix),
                      y       = BCellResistanceindex,
                      K       = K.opt,
                      eta     = i,
                      do.SIS  = TRUE,
                      pval    = pval,
                      adjust  = adjust)

    coefi <- coef(curr.fit.spls)
    coef.container[row.names(coefi), colidx] <- coefi

    colidx <- colidx + 1
}

## Sensitivity and specificity

all.coef    <-  as.vector(coef.container[ , eta == eta.opt])
all.pos     <-  as.vector(coef.container[fixed.list, eta == eta.opt])

n.truepos   <- length(all.pos [all.pos  != 0])
n.falsepos  <- length(all.coef[all.coef != 0]) - n.truepos

## Sensitivity
```

```
        BCell.reg.path.res$sens[reg.n] <- n.truepos / n.chosen

        ## Specificity

        BCell.reg.path.res$spec[reg.n] <-
            (length(coef.container[, 1]) - n.falsepos) /
             length(coef.container[, 1])

        ## False discovery rate

        BCell.reg.path.res$fdr[reg.n] <- 1 - n.truepos /
            length(all.coef[all.coef != 0])

        print(BCell.reg.path.res)

    }
    save(BCell.reg.path.res, coefi, fixed.list, coef.container,
         file = BCell.reg.path.res.files)
}
```

The resulting regularisation paths of the last simulation are shown in Figure 14.7.

```
> ts.col              <- rep("darkgrey", dim(coef.container)[1])
> ts.type             <- rep(2, dim(coef.container)[1])
> names(ts.col)       <- rownames(coef.container)
> names(ts.type)      <- rownames(coef.container)
> ts.col[fixed.list]  <- "black"
> ts.type[fixed.list] <- 1
> sparse.idx          <- apply(coef.container, 1, sum) != 0
> sparse.container    <- coef.container[sparse.idx, ]
> ts.col              <- ts.col[sparse.idx]
> ts.type             <- ts.type[sparse.idx]

> all.coef    <-  as.vector(coef.container[ , eta ==
                    BCell.reg.path.res$eta[nrow(BCell.reg.path.res)]])
> all.pos     <- as.vector(coef.container[fixed.list, eta ==
                    BCell.reg.path.res$eta[nrow(BCell.reg.path.res)]])
> n.truepos   <- length(all.pos [all.pos  != 0])
> n.falsepos  <- length(all.coef[all.coef != 0]) - n.truepos

> pdf(file.path(figure.output,"regularizationpath.pdf"))
> eta <- seq(0.2, 0.99, length.out = 200)
> plot(eta, sparse.container[1, ],
       type = "n",
       xlab = expression(eta),
```

```
       ylab = "Weight",
       main = "Regularization Path",
       xlim = c(0.65, 1),
       ylim = c(min(sparse.container), max(sparse.container)))
> for(i in 1:length(sparse.container[, 1])){
    if (ts.col[i] == "darkgrey")
      lines(eta, sparse.container[i, ],
            col = ts.col[i],
            lty = ts.type,
            lwd = 2)
  }
> for(i in 1:length(sparse.container[ , 1])){
    if (ts.col[i] == "black")
      lines(eta, sparse.container[i, ],
            col = ts.col[i],
            lty = ts.type,
            lwd = 2)
  }
> legend("topleft",
         legend = c("True", "False"),
         title  = "Probesets",
         bty    = "n",
         lty    = c(1, 1),
         col    = c( "black", "darkgrey"),
         lwd    = 2)
> dev.off()
```

Summary of the simulations are calculated and summarised i Table 14.4

```
> accuracy       <- matrix(NA, nrow = 2, ncol = 5)
> accuracy[1, ] <- round(apply(BCell.reg.path.res, 2, mean), 3)
> accuracy[2, ] <- round(apply(BCell.reg.path.res, 2, sd) /
                         sqrt(n.repeats - 1), 3)
```

Accuracy	sens	spec	fdr	eta	K
Mean	0.536	0.987	0.673	0.800	2.370
SD	0.039	0.002	0.024	0.012	0.071

Table 14.4: Summary of the repeated simulations.

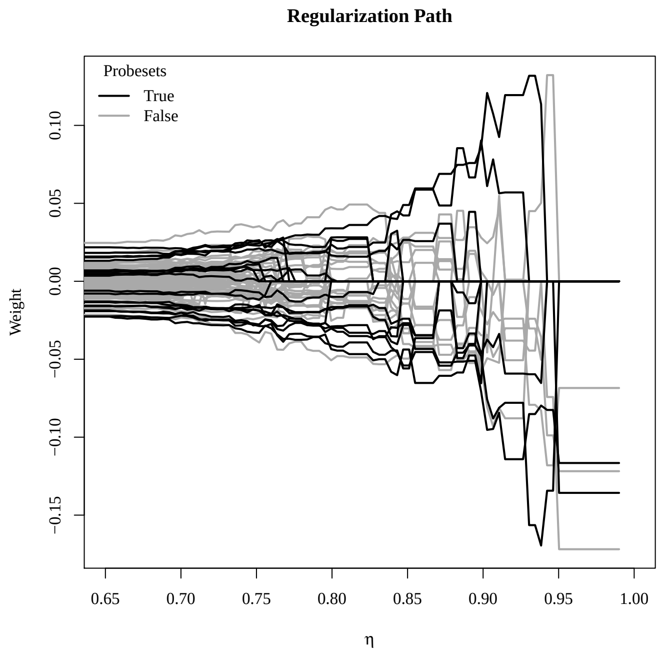

Figure 14.7: Regularization paths for the optimal number of partial least squares component $K = 3$ and regularization parameter $\eta = 0.827$ are shown. The true probesets have black lines and the false probesets are illustrated with grey lines. The sensitivity is 0.55, the specificity is 0.994 and the false discovery rate is 0.633.

15 Simple Analysis

In this section a resistance index based on the two cell lines *MOLP-2* and *RPMI-8226 LR5* is developed. Firstly, the duplicated Entrez Ids and control probes are removed using the `nsFilter` function `var.cutoff` set to 0.001.

```
> influential.data <- nsFilter(GEPBCell133a, var.cutoff = 0.001)$eset
```

The gene expressions of the two cell lines are extracted and stored in the object `influential.matrix`.

```
> influential.data <- influential.data[, c("MOLP-2", "RPMI-8226 LR5")]
> influential.matrix <- exprs(influential.data)
```

The data are sorted according to differences in the gene expressions between the two cell lines.

```
> diff.list <- apply(influential.matrix, 1, diff)
> sort.diff.list <- sort(diff.list)
> n.greatest <- 50
```

and the 100 most differentially expressed genes are used to establish the gene expression signature.

```
> len.list    <- length(sort.diff.list)
> most.diff.exprs <-
    sort.diff.list[c(1:n.greatest, (len.list - n.greatest + 1):len.list)]
> names.most <- names(most.diff.exprs)
> save(most.diff.exprs,
         file=file.path(BCell.gen.dir, "BCell.fit.simple.Rdata"))
```

The gene expressions are summarized in Table 15.3.

15.1 Predicting Melphalan Resistance in the *Arkansas* data

A melphalan resistance index is constructed by means of the gene expressions established in the previous section and used to predict the resistance index of the patients in the Arkansas data. Notice, the absolute difference is used as weight.

```
> BCellArkansasTest <-
    arkansas.matrix[names.most,]
> BCellArkansasindex <- my.predict(most.diff.exprs, BCellArkansasTest)[1,]
```

15.1.1 Association between the Melphalan Resistance Index and OS

Kaplan-Meier Survival Curves

Kaplan-Meier survival curves are constructed for the sensitive, intermediate and resistant groups. The 25% with the lowest predicted resistance index are categorized as sensitive and the 25% with the highest predicted melphalan resistance index are categorized as resistant. The remaining subjects are characterised as having intermediate resistance.

The Kaplan-Meier survival curves are plotted with the code chunk below and the result is shown in Figure 15.1.

```
> pdf(file.path(figure.output, "BCellarkansasOSKMplot_simple.pdf"))
> BCellarkansasOSKM_simple.P <-
      PlotKM(BCellArkansasindex, metadataArkansas$OS,
             cut.points = cut.points,
             xlab       = "Time (months)",
             ylab       = "OS ratio",
             xmax       = 110,
             main       = "Arkansas \n Kaplan-Meier OS curves")
> dev.off()
```

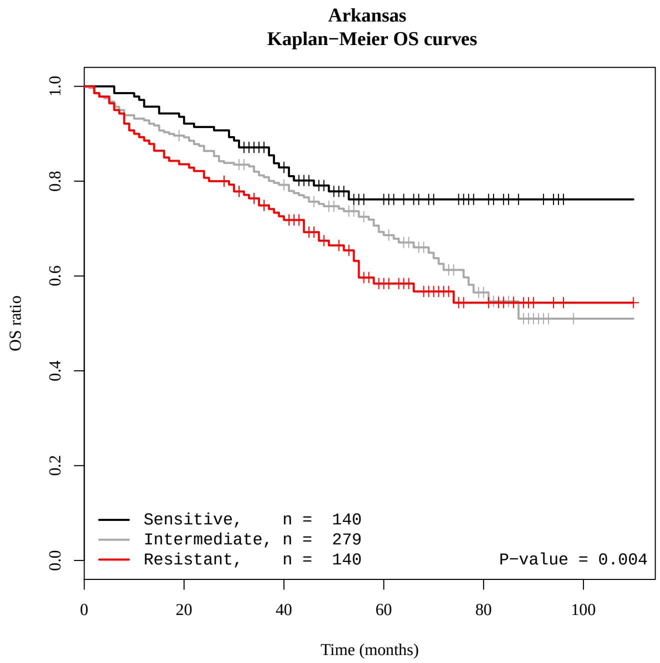

Figure 15.1: Kaplan-Meier survival curves based on the two cell lines resistance index. The logrank test comparing the survival curves results in a P-value of 0.004.

Cox Proportional Hazards

A Cox proportional hazards model is fitted were the association between log relative hazard and the two cell line resistance index is modelled by a spline with four knots.

```
> n.knots   <- 4
> coxfit.os <- coxph(metadataArkansas$OS ~ rcs(BCellArkansasindex, n.knots))
>
```

Table 15.1 summarizes three tests for no association between log relative hazard and the predicted resistance index.

Test	Test	D.o.F	P-value
Likelihood ratio test	7.41	3	0.0599
Wald test	8.06	3	0.0447
Score (logrank) test	8.28	3	0.0406

Table 15.1: A summary of three tests for no association between log relative hazard and the predicted resistance index.

.

The code chunk below produces a plot of the log relative hazard as a function of the two cell line resistance index. The result is depicted in Figure 15.2.

```
> pdf(file.path(figure.output, "BCellarkansasOSPredictors_simple.pdf"))
> par(mfrow=c(1,1))
> d <- datadist(BCellArkansasindex)
> options(datadist="d", width=150)
> BCellf <- cph(metadataArkansas$OS ~ rcs(BCellArkansasindex, n.knots))
> plot(BCellf,
       xlab="Fitted resistance index")
> title("Arkansas - OS Cox Proportional Hazards")
> legend("bottomright","",bty="n",
         title=paste("P-value = ",
         as.character(signif(unlist(BCellf)$stats.P,1)),"   ",sep=""))
> dev.off()
```

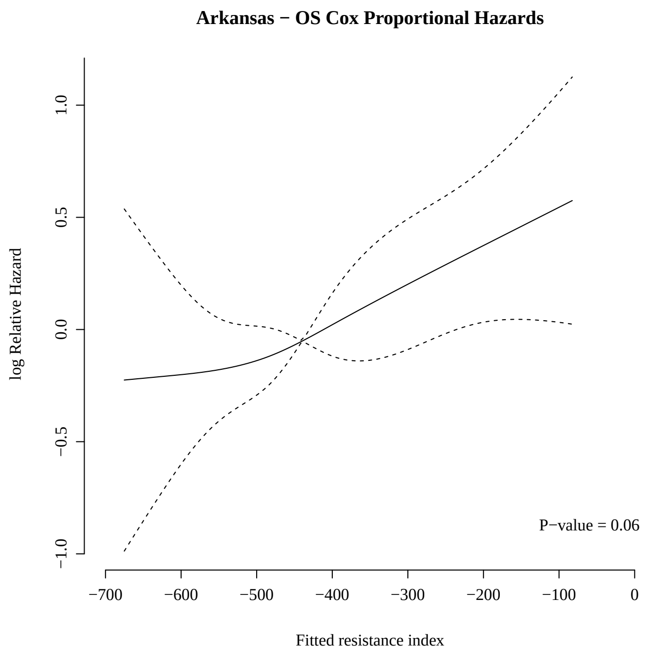

Figure 15.2: This figure shows the log relative hazard as a function of the two cell line resistance index. The P-value is the likelihood ratio test for no RCS-association between log relative hazard and resistance. The dashed lines represent 95% confidence intervals.

15.1.2 Association between the Melphalan Resistance Index and EFS

Kaplan-Meier Survival Curves

Similarly to the previous section Kaplan-Meier survival curves are made with the data grouped into sensitive, intermediate and resistant, however, EFS is used as endpoint instead of OS.

The Kaplan-Meier survival curves are plotted with the code chunk below and the result is shown in Figure 15.3.

```
> pdf(file.path(figure.output, "BCellarkansasEFSKMplot_simple.pdf"))
> BCellarkansasEFSKM_simple.P <-
      PlotKM(BCellArkansasindex,metadataArkansas$EFS,
             cut.points = cut.points,
             xlab       = "Time (months)",
             ylab       = "EFS ratio",
             xmax       = 110,
             main       = "Arkansas \n Kaplan-Meier EFS curves")
> dev.off()
```

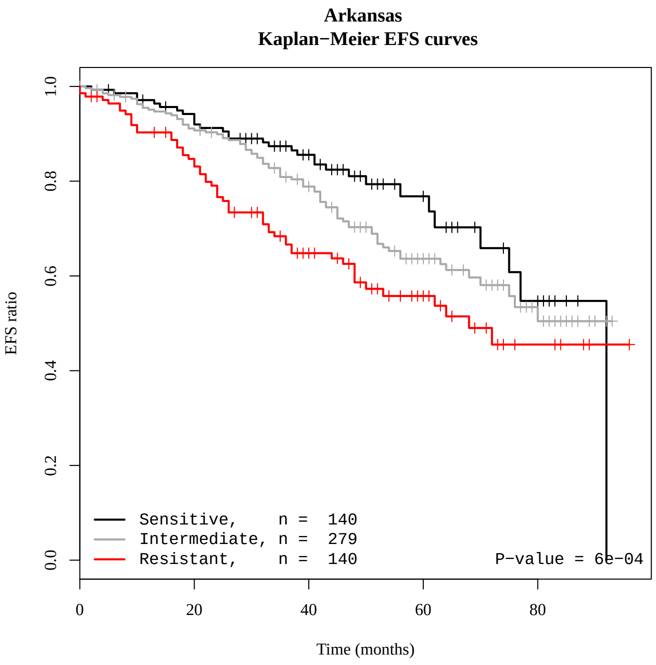

Figure 15.3: Kaplan-Meier survival curves based on the two cell lines resistance index. The logrank test comparing the survival curves results in a P-value of 6e-04.

Cox Proportional Hazards

A Cox proportional hazards model is fitted with the two cell line resistance index modelled using a spline with four knots.

```
> n.knots    <- 4
> coxfit.os <- coxph(metadataArkansas$EFS ~ rcs(BCellArkansasindex, 4))
```

Table 15.2 summarizes three tests for no association between log relative hazard and the predicted resistance index.

Test	Test	D.o.F	P-value
Likelihood ratio test	12.4	3	0.00620
Wald test	12.7	3	0.00533
Score (log rank) test	13.1	3	0.00449

Table 15.2: A summary of three tests for no association between log Relative Hazard and the predicted resistance index.

The code chunk below produces a plot of the log relative hazard as a function of the two cell line resistance index. The result is depicted in Figure 15.4.

```
> pdf(file.path(figure.output, "BCellarkansasEFSPredictors_simple.pdf"))
> par(mfrow=c(1,1))
> d <- datadist(BCellArkansasindex)
> options(datadist="d", width=150)
> BCellf <- cph(metadataArkansas$EFS ~ rcs(BCellArkansasindex, n.knots))
> plot(BCellf,
      xlab="Fitted resistance index")
> title("Arkansas BCell - EFS Cox Proportional Hazards")
> legend("bottomright","",bty="n",
         title=paste("P-value = ",
         as.character(signif(unlist(BCellf)$stats.P,1)),"   ",sep=""))
> dev.off()
```

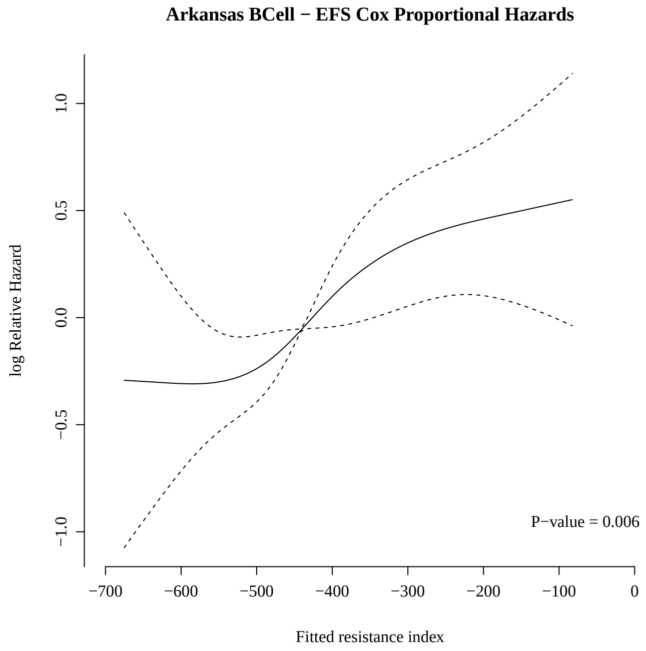

Figure 15.4: This figure shows the log relative hazard as a function of the two cell line resistance index. The P-value is the likelihood ratio test for no RCS-association between log relative hazard and resistance. The dashed lines represent 95% confidence intervals.

Finally, a table is created with the most differentially expressed genes, containing gene symbols and weights.

```
> genes.most    <- as.vector(unlist(lookUp(names.most, "hgu133plus2", "SYMBOL")))
> xx            <- matrix(NaN, nrow = n.greatest, ncol = 6)
> xx[, c(1, 4)] <- "as.vector<-"(xx[, c(1 ,4)],
                                 gsub("_", "\\\\textunderscore ", names.most))
> xx[, c(2, 5)] <- "as.vector<-"(xx[, c(2, 5)], genes.most)
> xx[, c(3, 6)] <- "as.vector<-"(xx[, c(3, 6)], round(most.diff.exprs, 3))
> colnames(xx) <- rep(c("U133a ID", "Gene Symbol", "Weight"), 2)
> latex.default(xx,
                title       = "Cell line",
                file        = "",
                booktabs    = TRUE,
                ctable      = FALSE,
                longtable   = TRUE,
                lines.page  = Inf,
                table.env   = TRUE,
                digits      = 3,
                label       = "tab:influentialnames",
                caption     = paste("Table of the gene expressions used in the two cell 
```

Table 15.3: Table of the gene expressions used in the two cell line resistance index.

U133a ID	Gene Symbol	Weight	U133a ID	Gene Symbol	Weight
205174_s_at	QPCT	-7.893	210942_s_at	ST3GAL6	5.596
205590_at	RASGRP1	-7.89	215189_at	KRT86	5.599
213831_at	HLA-DQA1	-7.591	212473_s_at	MICAL2	5.687
212122_at	RHOQ	-7.303	209735_at	ABCG2	5.69
210587_at	INHBE	-7.032	218502_s_at	TRPS1	5.717
39248_at	AQP3	-6.988	214023_x_at	TUBB2B	5.721
205990_s_at	WNT5A	-6.854	212724_at	RND3	5.752
202609_at	EPS8	-6.797	203474_at	IQGAP2	5.767
219159_s_at	SLAMF7	-6.793	204014_at	DUSP4	5.772
213938_at	ERC2	-6.724	213524_s_at	G0S2	5.774
204118_at	CD48	-6.706	217996_at	PHLDA1	5.797
207191_s_at	ISLR	-6.422	206897_at	PAGE1	5.824
205801_s_at	RASGRP3	-6.387	218736_s_at	PALMD	5.835
212998_x_at	HLA-DQB1	-6.351	204734_at	KRT15	5.978
212063_at	CD44	-6.321	214039_s_at	LAPTM4B	6.004
220132_s_at	CLEC2D	-6.251	204533_at	CXCL10	6.053
221558_s_at	LEF1	-6.186	205098_at	CCR1	6.107
219073_s_at	OSBPL10	-6.165	221698_s_at	CLEC7A	6.122

Table 15.3: *(continued)*

U133a ID	Gene Symbol	Weight	U133a ID	Gene Symbol	Weight
219667_s_at	BANK1	-6.147	202551_s_at	CRIM1	6.174
204971_at	CSTA	-6.137	203666_at	CXCL12	6.201
219049_at	CSGALNACT1	-6.13	204066_s_at	AGAP1	6.228
201998_at	ST6GAL1	-5.816	44790_s_at	C13orf18	6.246
206150_at	CD27	-5.799	204105_s_at	NRCAM	6.259
211990_at	HLA-DPA1	-5.789	220415_at	TNNI3K	6.366
219926_at	POPDC3	-5.752	209803_s_at	PHLDA2	6.427
208683_at	CAPN2	-5.736	202350_s_at	MATN2	6.463
207734_at	LAX1	-5.673	203917_at	CXADR	6.479
220129_at	SOHLH2	-5.534	207979_s_at	CD8B	6.512
202252_at	RAB13	-5.527	200606_at	DSP	6.739
205671_s_at	HLA-DOB	-5.512	212599_at	AUTS2	6.791
200999_s_at	CKAP4	-5.435	202677_at	RASA1	6.806
213638_at	PHACTR1	-5.405	201445_at	CNN3	6.812
202746_at	ITM2A	-5.381	204151_x_at	AKR1C1	6.908
209942_x_at	MAGEA3	-5.351	204485_s_at	TOM1L1	6.909
203708_at	PDE4B	-5.323	219181_at	LIPG	6.931
209366_x_at	CYB5A	-5.277	212192_at	KCTD12	7.011
203895_at	PLCB4	-5.25	221210_s_at	NPL	7.063
213170_at	GPX7	-5.208	205549_at	PCP4	7.135
221297_at	GPRC5D	-5.158	209699_x_at	AKR1C2	7.169
201925_s_at	CD55	-5.155	201667_at	GJA1	7.485
221571_at	TRAF3	-5.155	210095_s_at	IGFBP3	7.581
219371_s_at	KLF2	-5.132	206336_at	CXCL6	7.688
205884_at	ITGA4	-5.09	205898_at	CX3CR1	7.769
201952_at	ALCAM	-5.018	212094_at	PEG10	7.778
206609_at	MAGEC1	-5.014	201427_s_at	SEPP1	8.534
203397_s_at	GALNT3	-4.979	209348_s_at	MAF	8.855
202947_s_at	GYPC	-4.966	209395_at	CHI3L1	9.056
205632_s_at	PIP5K1B	-4.953	209160_at	AKR1C3	9.105
210889_s_at	FCGR2B	-4.935	205336_at	PVALB	9.142
209619_at	CD74	-4.926	211719_x_at	FN1	9.776

16 Auxiliary Functions

16.1 Dose Response Functions

Function for extracting members of x which are not present in y.

```
> "%w/o%" <- function(x, y) x[!x %in% y]

> "as.vector<-" <- function(xx, y){
      d <- nrow(xx) * ncol(xx) - length(y)
      if(d != 0){y <- c(y, rep("", d))}
      h <- 0:(ncol(xx) - 1) * nrow(xx) + 1
      h2 <- c(h[ - 1] - 1, length(y))
      for(i in 1:ncol(xx)){
          xx[,i] <-  y[h[i]:h2[i]]
      }
      return(xx)
  }
```

NCI60 dose extraction.

```
> getNSC <- function(filename, target, path = NULL) {
      ## Eat the extra final spaces
      fixup <- function(x) {
          x <- as.character(x)
          x <- sub(" $", "", x)
          factor(x)
      }
      ## Load the data and clean it up
      if(!is.null(path)) {
          filename <- file.path(path, filename)
      }
      rt <- read.table(filename,
                       sep=',', header = TRUE, row.names = NULL,
                       quote = '', comment.char = '')
      rt$NSC           <- factor(rt$NSC)
      rt$LCONC         <- factor(rt$LCONC)
      temp             <- as.character(rt$STDDEV)
      temp             <- gsub(" ", '', temp)
      temp[temp=="."] <- NA
      rt$STDDEV        <- as.numeric(temp)
      rt$PANEL         <- fixup(rt$PANEL)
      rt$CELL          <- fixup(rt$CELL)
      rt[, "Key"]      <- factor(paste(as.character(rt$NSC),
```

```
                                as.character(rt$LCONC), sep='L'))
    len <- table(rt$CELL, rt$Key)

    # Use groupedData and gsummary to average over meaningless replicates
    form       <- dummy ~ 1|NSC/LCONC/CELL
    form[[2]] <- as.name(target)
    gd         <- groupedData(form, data = rt)
    simple     <- gsummary(gd, mean, na.rm = TRUE)
    result     <- tapply(simple[, target], list(simple$CELL, simple$Key),
                         mean, na.rm = TRUE)
    result
}
```

Loading .DBF files into R

```
> readDBFMeta <- function(path = getwd(), metadata, remove.edge = TRUE){

    metadata$h2 <- paste(metadata$Name, metadata$Drug.s., sep=":")
    h4           <- (aggregate(rep(1, length(metadata$Drug.s.)),
                          metadata[, c("Name", "Drug.s.")], sum))

    for(i in metadata$h2){
        metadata$platerep[metadata$h2==i] <-
            1:h4$x[paste(h4$Name, h4$Drug.s., sep=":")==i]
    }

    chemoIndex <- vector()
    celllines  <- vector()
    drug       <- vector()
    rep        <- vector()
    content    <- vector()
    colnum     <- vector()
    wellnum    <- vector()
    plate      <- vector()
    plateset   <- vector()
    time       <- vector()
    platerep   <- vector()
    disease    <- vector()
    for(i in 1:length(metadata[metadata$Drug.s.])){

        file0  <- metadata$Data.file.day.1[i]
        file48 <- metadata$Data.file.day.3[i]

        ## Plate 1 is read into R
```

```
cell0        <-  read.dbf(paste(path, "/", file0, ".dbf", sep=""))
cell0        <-  cell0[-(1:2), ]
cell0$COLNUM <- factor(substr(cell0$WELLNUM, 2, 3))
cell0$ROW    <- factor(substr(cell0$WELLNUM, 1, 1))
cell0 <- cell0[cell0$COLNUM != "01" &
               cell0$COLNUM != "12", ]
if(remove.edge == TRUE){
    cell0 <- cell0[cell0$ROW    != "A"  &
                   cell0$ROW    != "H",  ]
}

cell0 <- cell0[order(cell0$CONTENT), ]

if(remove.edge == TRUE){
    cell0$REP <- rep(1:3, 20)
}else{
    cell0$REP <- rep(1:4, 20)
}

## Plate 2 is read into R
cell48        <- read.dbf(paste(path, "/", file48, ".dbf", sep=""))
cell48        <- cell48[ - (1:2), ]
cell48$COLNUM <- factor(substr(cell48$WELLNUM, 2, 3))
cell48$ROW    <- factor(substr(cell48$WELLNUM, 1, 1))
cell48 <- cell48[cell48$COLNUM != "01" &
                 cell48$COLNUM != "12", ]

if(remove.edge == TRUE){

    cell48    <- cell48[cell48$ROW != "A"  &
                        cell48$ROW != "H", ]
}

cell48 <- cell48[order(cell48$CONTENT), ]

if(remove.edge == TRUE){
    cell48$REP <- rep(1:3, 20)
}else{
    cell48$REP <- rep(1:4, 20)
}



n <- length(cell0 [, "M1"])
```

```
        m <- length(cell48[, "M1"])

        chemoIndex <- c(chemoIndex, cell0[, "M1"], cell48[, "M1"])
        celllines  <- c(celllines,  rep(paste(metadata$Name[i]),    n + m))
        drug       <- c(drug,       rep(paste(metadata$Drug.s.[i]), n + m))
        disease    <- c(disease,    rep(paste(metadata$Disease[i]), n + m))
        rep        <- c(rep,        cell0[, "REP"], cell48[, "REP"])
        content    <- c(content,    as.character(cell0 [, "CONTENT"]),
                                    as.character(cell48[, "CONTENT"]))
        colnum     <- c(colnum,     as.character(cell0 [, "COLNUM"]),
                                    as.character(cell48[, "COLNUM"]))
        time       <- c(time,       rep(1, n), rep(3, m))
        wellnum    <- c(wellnum,    as.character(cell0 [, "WELLNUM"]),
                                    as.character(cell48[, "WELLNUM"]))
        plate      <- c(plate,      rep(paste(i * 2 - 1), n), rep(i * 2, m))
        plateset   <- c(plateset,   rep(i, ( n + m)))
        platerep   <- c(platerep,   rep(metadata$platerep[i], (n + m)))


    }

    chemoIndex <- data.frame(index    = chemoIndex, drug     = drug,
                             name     = celllines,  rep      = rep,
                             wellnum  = wellnum,    content  = content,
                             colnum   = colnum,     time     = time,
                             plate    = plate,      plateset = plateset,
                             platerep = platerep,   disease  = disease)


  }
>
```

Expressing dose in molaer

```
> molaer <- function(x){
      log10(1000*x/305.2/1000000)
  }
```

Calculate means using the bootstrap.

```
> meanBootstrap <- function(x){
      n <- length(x)
      b <- sample(x,n,replace=TRUE)
      mean(b)
  }
```

```
> logistic <- function(x,a,b){

    2*(10^(a+b*x)/(1+10^(a+b*x))) - 1

    2*exp(-a*x^2) - 1
  }
```

Determine whether or not there are outliers.

```
> grubbsTestPValue <- function(X){
      test <- grubbs.test(X)
      Grubbs.P.value <- test$p.value
      return(Grubbs.P.value)
  }
```

Extract the replicates which need to be excluded.

```
> grubbsTestHL <- function(X){
      test <- grubbs.test(X)
         x <- strsplit(test$alternative, " ")
         y <- as.numeric(x[[1]][3])
      return(y)
  }
```

Perform Grubbs test on the data.

```
> addGrubbs <- function(X, p = 0.05, grubbs = 1){

      grubbs.p.value <-  aggregate(X$index,
                                   X[, c("plate", "content")],
                                   grubbsTestPValue )

      names(grubbs.p.value)[3] <- "grubbs.p.value"

      grubbs.hl <-  aggregate(X$index,
                              X[, c("plate", "content")],
                              grubbsTestHL)

      names(grubbs.hl)[3] <- "grubbs.hl"

      X <- merge(X, grubbs.p.value, by = c("plate", "content"))
      X <- merge(X, grubbs.hl,      by = c("plate", "content"))

      X$outlier <- ifelse(X$index == X$grubbs.hl &
                          X$grubbs.p.value < p, 1, 0)
```

```
    excluded <- dim(X[X$outlier == 1, ])[1]
    X2 <- X
    X <- X[X$outlier == 0, ]

    if(grubbs == 2){
      grubbs.p.value <-  aggregate(X$index,
                                 X[, c("plate", "content")],
                                   grubbsTestPValue )

      names(grubbs.p.value)[3] <- "grubbs.p.value2"

      grubbs.hl <-  aggregate(X$index,
                           X[, c("plate", "content")], grubbsTestHL)
      names(grubbs.hl)[3] <- "grubbs.hl2"

      X <- merge(X, grubbs.p.value, by=c("plate", "content"))
      X <- merge(X, grubbs.hl,      by=c("plate", "content"))

      X$outlier2 <- ifelse(X$index == X$grubbs.hl2 &
                           X$grubbs.p.value2 < p, 1, 0)

      excluded <- c(excluded, dim(X[X$outlier2 == 1, ])[1])
      X3 <- X
      X <- X[X$outlier2 == 0, ]

    }
    if(grubbs == 1){X3 <- X}
    return(list(X, excluded, X2, X3))
}
```

Function for background correction of the plates.

```
> backgroundCorrected <- function(X, Bootstrap = FALSE, bcormethod = "difference"){

    y <- vector()
    mean.all.b <- mean(X$index[X$outlier == 0 &
                             X$content == "B"])

    if(Bootstrap == FALSE){
        for(i in unique(X$plate)){
            mean.b <- mean(X$index[X$outlier == 0 &
                                   X$content == "B" &
                                   X$plate   == i])
```

```
            if(bcormethod == "difference"){
                y <- c(y, (X$index[X$plate == i] - mean.b))
            }else{
                y <- c(y, (X$index[X$plate == i] / mean.b))
            }

        }
    }else{
        for(i in unique(X$plate)){
            mean.b <- meanBootstrap(X$index[X$outlier == 0 &
                                            X$content == "B" &
                                            X$plate   == i])

            if(bcormethod == "difference"){
                y <- c(y, (X$index[X$plate == i] - mean.b))
            }else{
                y <- c(y, (X$index[X$plate == i] / mean.b))
            }

        }
    }
    X$backgroundCorrected <- y
    X$backgroundCorrected[X$content == "B"] <- X$index[X$content == "B"]

    return(X)
}
```

Ensure that the absorbance values lie within a certain interval.

```
> diffsetCorrection <- function(X, int.cor=c(0.2, 1.2), model = "R2"){

    X <- X[X$backgroundCorrected >= int.cor[1] & X$backgroundCorrected <= int.cor[2], [illegible]

    if(model %in% c("D1", "R1")){
        for(i in unique(X$plateset)){

            diffset  <- setdiff(X$content[X$plateset == i & X$time == 1],
                                X$content[X$plateset == i & X$time == 3])

            diffset2 <- setdiff(X$content[X$plateset == i & X$time == 3],
                                X$content[X$plateset == i & X$time == 1])

            diffset  <- c(diffset, diffset2)
```

```
            X <- X[!(X$content %in% diffset & X$plateset == i), ]
        }
    }
    return(X)
  }

> D1 <- function(x, y, z) {
    res <- ifelse(x >= 0, x / (y-z[1]), x / z[1])
    return(res)
  }
>
```

This function calculates the growth inhibition through various models. It sets the model to be used for calculating GI: MA uses moving average to smooth the curves giving a more stable result. If the Bootstrap options is TRUE the function performs bootstrap at the well level.

```
> GI <- function(X, model = "R2", MA = FALSE, Bootstrap = FALSE) {

    X <- X[X$content != "B", ]

    ## Calculate the mean or the replicated wells
    if(Bootstrap == FALSE) {
        X2 <- aggregate(X$backgroundCorrected,
                        X[, c("plate", "content")],
                        mean)
    }else{
        X2 <- aggregate(X$backgroundCorrected,
                        X[, c("plate", "content")],
                        meanBootstrap)
    }

    ## Merge the datasets
    X <- merge(X2, X, by=c("plate", "content"), all=T)

    ## Use only use one of the replicates
    X2 <- aggregate(X$rep,
                    X[, c("plate", "content")],
                    min)

    X2 <- rename.vars(X2, "x", "keep")
    X  <- merge(X2, X, by = c("plate", "content"), all = T)
    X  <- X[X$keep == X$rep, ]
```

```
X$plate  <- as.numeric(X$plate)

GI       <- vector()
GIname   <- vector()
drug     <- vector()
plateset <- vector()
platerep <- vector()
disease  <- vector()
t        <- vector()
name     <- vector()

X <- X[order(X$plate), ]

for(i in unique(X$plateset)) {
    drug.i <- paste(X$drug[X$plateset == i][1])
    if(model == "D1"){
              X0  <-       X$x[X$plateset == i & X$time == 1]
        names(X0) <- X$content[X$plateset == i & X$time == 1]
              X1  <-       X$x[X$plateset == i & X$time == 3]
        names(X1) <- X$content[X$plateset == i & X$time == 3]

        diff      <- X1 - X0[c("X1")]
        GIres     <- D1(diff, X1[c("X1")], X0)
    }


    if(model == "D3"){
              X0  <-       X$x[X$plateset == i & X$time == 1]
        names(X0) <- X$content[X$plateset == i & X$time == 1]
              X1  <-       X$x[X$plateset == i & X$time == 3]
        names(X1) <- X$content[X$plateset == i & X$time == 3]
              # The first six are used as reference on plate 1
              x0  <- mean(X0[c(paste("X",1:6,sep = ""))])
              x1  <- mean(X1[c(paste("X",1:4,sep = ""))])
              diff      <- X1 - x0
              # The first four are used as reference on plate 2
        GIres     <- ifelse(diff >= 0, diff / (x1 - x0), diff / x0)
    }

    if(model == "D4"){
        sets <- unique(X$plateset[X$name == X$name[X$plateset == i]])
              X0  <-       X$x[X$plateset == i & X$time == 1]
        names(X0) <- X$content[X$plateset == i & X$time == 1]
```

```
        X1  <-       X$x[X$plateset == i & X$time == 3]
  names(X1) <- X$content[X$plateset == i & X$time == 3]
        # The first six are used as reference on plate 1
        x0  <- mean(X0[c(paste("X",1:6,sep = ""))])
        x1  <- mean(X1[c(paste("X",1:4,sep = ""))])
  diff      <- X1 - x0
        # The first four are used as reference on plate 2
  GIres     <- ifelse(diff >= 0, diff / (x1 - x0), diff / x0)
}


if(model == "D2"){
        X0  <-       X$x[X$plateset == i & X$time == 1]
  names(X0) <- X$content[X$plateset == i & X$time == 1]
        X1  <-       X$x[X$plateset == i & X$time == 3]
  names(X1) <- X$content[X$plateset == i & X$time == 3]

  diff      <- X1 - X0[1]
  GIres     <- D1(diff, X1[c("X1")], X0)
}

if(model == "R1"){
        X0  <-       X$x[X$plateset == i & X$time == 1]
  names(X0) <- X$content[X$plateset == i & X$time == 1]
        X1  <-       X$x[X$plateset == i & X$time == 3]
  names(X1) <- X$content[X$plateset == i & X$time == 3]

  diff      <- X1/X0
  GIres     <- diff/diff[c("X1")]

}

if(model == "R2"){
        X1  <-       X$x[X$plateset == i & X$time == 3]
  names(X1) <- X$content[X$plateset == i & X$time == 3]

  diff      <- X1
  GIres     <- X1/X1[c("X1")]
}

if(model == "R3"){
        X1  <-       X$x[X$plateset == i & X$time == 3]
  names(X1) <- X$content[X$plateset == i & X$time == 3]
```

```
        diff      <- X1
        GIres     <- X1/mean(X1[c(paste("X", 1:4, sep = ""))])
    }

    GI        <- c(GI, GIres)
    h1        <- substr(names(diff[names(diff) != "B"]), 2, 10)
    h1        <- as.numeric(h1) -1
    GIname    <- c(GIname, h1)
    plateset <- c(plateset, rep(i, length(diff[names(diff) != "B"])))
    platerep <- c(platerep, rep(paste(X$platerep[X$plateset == i][1]),
                                length(diff[names(diff) != "B"])))
    drug      <- c(drug, rep(drug.i, length(diff[names(diff) != "B"])))
    disease   <- c(disease, rep(paste(X$disease[X$plateset == i][1]),
                                length(diff[names(diff) != "B"])))
    name      <- c(name, rep(paste(X$name[X$plateset == i][1]),
                            length(diff[names(diff) != "B"])))
    t         <- c(t, as.numeric(metadata[i, paste("C", h1, sep="")]))
}

X <- data.frame(name     = name,     drug     = drug,
                plateset = plateset, platerep = platerep,
                disease  = disease,  GIname   = GIname,
                GI       = GI,       t=t,  t2 = log10(t))

X <- X[order(X$t), ]
X <- X[order(X$plateset), ]
X <- X[X$GIname!="0", ]
X <- X[order(X$name), ]
X <- X[order(X$disease), ]
X <- X[order(X$drug), ]

X$name    <- as.factor(as.character(X$name))
X$disease <- as.factor(as.character(X$disease))
X$drug    <- as.factor(as.character(X$drug))

if(MA!=FALSE){
    X$trial <- paste(X$drug, X$name, X$platerep, sep=":")
    ta      <- tapply(X$GI, c(X$trial), pSMA)
    names   <- dimnames(ta)
    MAGI    <- vector()

    for(i in (names[[1]])){
        MAGI <- c(MAGI, eval(parse(text=paste("ta$", "'", i,
                                  "'", sep=""))))
```

```
        }

        X$MAGI <- MAGI
    }
    return(X)
}
```

This function is used for the establishment of the GI_{50} values by use of functions previously defined.

```
> createGIData <- function(X      = chemo.index, grubbs     = 1,
                           b.cor  = TRUE,        int.cor    = FALSE,
                           model  = "R2",        MA         = FALSE,
                           p      = 0.01,        Bootstrap  = FALSE,
                           log    = FALSE,       bcormethod = "difference"){

      pSMA <- function(x)
      {
          SMA(x,  n=MA)
      }

      if(grubbs == 1){
                 x <- addGrubbs(X, p = p, grubbs = 1)
          excluded <- x[[2]]
                 X <- x[[1]]
      }

      if(grubbs == 2){
                 x <- addGrubbs(X, p = p, grubbs = 2)
          excluded <- x[[2]]
                 X <- x[[1]]
      }

      if(grubbs == FALSE){
          X$outlier <- 0
          excluded  <- 0
      }

      #if(log == TRUE){X$index <- log(X$index)}
      if(b.cor == TRUE){
          X <- backgroundCorrected(X, Bootstrap = Bootstrap,
                                      bcormethod = bcormethod)
      }else{
          X <- X[X$content!="B", ]
```

```
        X$backgroundCorrected <- X$index
    }

    if(int.cor != FALSE){
        X <- diffsetCorrection(X, int.cor = int.cor)
    }

    if(log == TRUE){X$index <- log(X$index)}

    X <- GI(X, model = model, MA = MA,
            Bootstrap = Bootstrap)

    return(list(X,excluded))
}
```

Function for extracting the GI_{50} values

```
> findGI <- function(x, y, GI = 0.5){
    if(y[1] <- GI){
        res <- x[1]
    }else{
        res <- Inf
    }
    n <- length(y)
    i <- 1
  repeat{
    if(y[i + 1] < GI & i < (n-1)){
      a <- (y[i + 1] - y[i]) /(x[i + 1] - x[i])
      b <- - 1 * a * x[i] + y[i]
      res <- (GI - b) / a
      break()
    }
    i <- i + 1
  }
  res
}
```

16.2 Model Building Functions

16.3 Functions used for the SPLS Analysis

The function `SIS` defined below performs Sure Independence Screening. The output of the function is the P-value for the null-hypothesis of no correlation. The P-values may be adjusted through the following metods:

holm, hochberg, hommel, bonferroni, BH, BY, fdr, none.

```
> SIS <- function(exprs, index, adjust = p.adjust.methods){

      n <- dim(exprs)[2]
      p <- dim(exprs)[1]

      if(class(index)  ==  "matrix"){
          names <- row.names(index)
      }else{
          names <- names(index)
      }

      if(class(exprs)  ==  "ExpressionSet"){
          exprs <- exprs(exprs)
      }

      x <- t(scale(t(exprs)))
      y <- scale(index)

      sis.score <- (x %*% y)[, 1] / (n - 1)
      sis.cor  <- sis.score / sqrt((1 - sis.score^2) / (n - 2))
      sis.pval  <- 2 * pt( - 1 * abs(sis.cor), n - 2)
      p.adjust <- p.adjust(sis.pval, method = adjust)

      return(p.adjust)
  }
```

The function SISCV performs SIS in a way suitable for CV.

```
> SISCV <- function(exprs, index, adjust = p.adjust.methods,
                    fold = length(index), foldi = "gen"){

      n <- dim(exprs)[2]
      p <- dim(exprs)[1]

      if(class(index) == "matrix"){
          names <- row.names(index)
      }else{
          names <- names(index)
      }

      if(class(exprs) == "ExpressionSet"){
          exprs <- exprs(exprs)
      }
```

```
    if(foldi[1] == "gen"){foldi <- split(sample(1:n), rep(1:fold, length = n))}

    sis.cv <- matrix(0, ncol = fold, nrow = p)
    sis.cv.adjust <- matrix(0, ncol = fold, nrow = p)

    rownames(sis.cv) <- row.names(exprs)
    rownames(sis.cv.adjust) <- row.names(exprs)

    cname <- vector()
    for (j in 1:fold) {
        cname[j] <-  paste(names[foldi[[j]]], collapse = ", ")
        omit <- foldi[[j]]
        x <- t(scale(t(exprs[, - omit])))
        y <- scale(index[ - omit])
        sis.cv[,j] <- SIS(exprs = x, index = y, adjust = "none")
        sis.cv.adjust[,j] <- SIS(exprs = x, index = y, adjust = adjust)
    }
    colnames(sis.cv) <- cname
    colnames(sis.cv.adjust) <- cname
    class(sis.cv) <- "SISCV"
    class(sis.cv.adjust) <- "SISCV"
    return(list(sis.cv,sis.cv.adjust))
  }

> summary.SISCV <- function(SISCV, p.value = 0.05){
    nSignificant <- function(x){
        length(x[x <= p.value])
    }
    return(apply(SISCV, 2, nSignificant))
  }

> View.SISCV <- function(SISCV){
    class(SISCV) <- "matrix"
    View(SISCV)
  }
```

Construction of a filter function which only finds the correlation coefficient between genes and chemosensitivity, P-value, and FDR for the hypothesis of no correlation

```
> corFilter <- function(exprs.matrix,chemoindex,alpha=0.01){
    cor.filter <- function(x){
        fit <- cor.test(x,chemoindex)
        return(c(fit$estimate,fit$p.value))
    }
```

```
  # FDR is per default set to 0.01
    probelist <- genefilter(exprs.matrix,cor.filter)
    probelistLength <- length(probelist[1,])
    probelistNumber <- 1:probelistLength
    probelist <- rbind(probelist,0)
    row.names(probelist) <- c("cor","pValue","reject")
    probelist <- t(probelist)
    probelist <- probelist[order(probelist[,2]),]
    bool <- probelist[,2] - alpha * probelistNumber/probelistLength
    probelist[,3] <- bool<=0
    a <- 1:length(probelist[,3])
    if(length(a[bool<=0])>0){
        b <- max(a[bool<=0])
        probelist[,3][1:b] <- 1
    }
    return(probelist)
}
```

In order to use the SPLS functions together with SIS the three functions `spls`, `cv.spls` and `predict.spls` from the package `spls` are recoded.

```
> spls <- function (x, y, K, eta, kappa = 0.5, select = "pls2", fit = "simpls",
                    scale.x = TRUE, scale.y = FALSE, eps = 1e-04, maxstep = 100,
                    trace = FALSE, do.SIS = FALSE, pval = 0.05, adjust = p.adjust.method
  {
    if(do.SIS){
        sis <- SIS(t(x), y, adjust = adjust)
        sis.names <- names(sis[sis <= pval])
        if(length(sis.names) < max(K)){
            sis.names <- names(sort(sis)[1:max(K)])
        }
        x <- x[, sis.names]
    }
    x <- as.matrix(x)
    n <- nrow(x)
    p <- ncol(x)
    ip <- c(1:p)
    y <- as.matrix(y)
    q <- ncol(y)
    one <- matrix(1, 1, n)
    mu <- one %*% y/n
    y <- scale(y, drop(mu), FALSE)
    meanx <- drop(one %*% x)/n
    x <- scale(x, meanx, FALSE)
```

```
if (scale.x) {
    normx <- sqrt(drop(one %*% (x^2))/(n - 1))
    if (any(normx < .Machine$double.eps)) {
        stop("Some of the columns of the predictor matrix have zero variance.")
    }
    x <- scale(x, FALSE, normx)
} else {
    normx <- rep(1, p)
}
if (scale.y) {
    normy <- sqrt(drop(one %*% (y^2))/(n - 1))
    if (any(normy < .Machine$double.eps)) {
        stop("Some of the columns of the response matrix have zero variance.")
    }
    y <- scale(y, FALSE, normy)
}  else {
    normy <- rep(1, q)
}
betahat <- matrix(0, p, q)
betamat <- list()
x1 <- x
y1 <- y
type <- correctp(x, y, eta, K, kappa, select, fit)
eta <- type$eta
K <- type$K
kappa <- type$kappa
select <- type$select
fit <- type$fit
if (is.null(colnames(x))) {
    xnames <- c(1:p)
}  else {
    xnames <- colnames(x)
}
new2As <- list()
if (trace) {
    cat("The variables that join the set of selected variables at each step:\n")
}
for (k in 1:K) {
    Z <- t(x1) %*% y1
    what <- spls.dv(Z, eta, kappa, eps, maxstep)
    A <- unique(ip[what != 0 | betahat[, 1] != 0])
    new2A <- ip[what != 0 & betahat[, 1]  ==  0]
    xA <- x[, A, drop = FALSE]
    plsfit <- plsr(y ~ xA, ncomp = min(k, length(A)), method = fit,
```

```
                  scale = FALSE)
    betahat <- matrix(0, p, q)
    betahat[A, ] <- matrix(coef(plsfit), length(A), q)
    betamat[[k]] <- betahat
    pj <- plsfit$projection
    if (select  ==  "pls2") {
        y1 <- y - x %*% betahat
    }
    if (select  ==  "simpls") {
        pw <- pj %*% solve(t(pj) %*% pj) %*% t(pj)
        x1 <- x
        x1[, A] <- x[, A, drop = FALSE] - x[, A, drop = FALSE] %*%
            pw
    }
    new2As[[k]] <- new2A
    if (trace) {
        if (length(new2A) <= 10) {
            cat(paste("- ", k, "th step (K = ", k, "):\n",
                      sep = ""))
            cat(xnames[new2A])
            cat("\n")
        }
        else {
            cat(paste("- ", k, "th step (K = ", k, "):\n",
                      sep = ""))
            nlines <- ceiling(length(new2A)/10)
            for (i in 0:(nlines - 2)) {
                cat(xnames[new2A[(10 * i + 1):(10 * (i + 1))]])
                cat("\n")
            }
            cat(xnames[new2A[(10 * (nlines - 1) + 1):length(new2A)]])
            cat("\n")
        }
    }
}
if (!is.null(colnames(x))) {
    rownames(betahat) <- colnames(x)
}
if (q > 1 & !is.null(colnames(y))) {
    colnames(betahat) <- colnames(y)
}
object <- list(x = x, y = y, betahat = betahat, A = A, betamat = betamat,
               new2As = new2As, mu = mu, meanx = meanx, normx = normx,
               normy = normy, eta = eta, K = K, kappa = kappa, select = select,
```

```
                 fit = fit, projection = pj)
    class(object) <- "spls"
    object
}

> cv.spls <- function (x, y, fold = 10, K, eta, kappa = 0.5, select = "pls2",
                     fit = "simpls", scale.x = TRUE, scale.y = FALSE,
                     plot.it = TRUE, do.SIS = FALSE, pval = 0.05,
                     adjust = p.adjust.methods) {

    x <- as.matrix(x)
    n <- nrow(x)
    p <- ncol(x)
    ip <- c(1:p)
    y <- as.matrix(y)
    q <- ncol(y)
    type <- correctp(x, y, eta, K, kappa, select, fit)
    eta <- type$eta
    K <- type$K
    kappa <- type$kappa
    select <- type$select
    fit <- type$fit
    foldi <- split(sample(1:n), rep(1:fold, length = n))
    mspemat <- matrix(0, length(eta), length(K))
    if(do.SIS == TRUE){
        cat(paste("SIS", "\n"))
        sis.cv <- SISCV(exprs  = t(x),
                        index  = y,
                        adjust = adjust,
                        fold   = fold,
                        foldi  = foldi)[[2]]
    }
    for (i in 1:length(eta)) {
        cat(paste("eta = ", eta[i], "\n"))
        mspemati <- matrix(0, fold, length(K))
        for (j in 1:fold) {
            omit <- foldi[[j]]

            if(do.SIS == TRUE){
                sis <- sis.cv[, j]
                sis.names <- names(sis[sis <= pval])
                if(length(sis.names) < max(K)){
                    sis.names <- names(sort(sis)[1:max(K)])
                }
```

```
            }else{
                sis.names <- colnames(x)
            }


            object <- spls(x[-omit, sis.names, drop = FALSE], y[-omit,
                                  , drop = FALSE], eta = eta[i], kappa = kappa,
                           K = max(K), select = select, fit = fit, scale.x = scale.x,
                           scale.y = scale.y, trace = FALSE,
                           do.SIS = FALSE, pval = pval, adjust = adjust)

            newx <- x[omit, colnames(object$x), drop = FALSE]
            newx <- scale(newx, object$meanx, object$normx)
            betamat <- object$betamat
            for (k in K) {
                pred <- newx %*% betamat[[k]] + matrix(1, nrow(newx),
                                                       1) %*% object$mu
                mspemati[j, (k - min(K) + 1)] <- mean(apply((y[omit,
                                                               ] - pred)^2, 2, mean))
            }
        }
        mspemat[i, ] <- apply(mspemati, 2, mean)
    }
    minpmse <- min(mspemat)
    rownames(mspemat) <- eta
    colnames(mspemat) <- K
    mspecol <- apply(mspemat, 2, min)
    msperow <- apply(mspemat, 1, min)
    K.opt <- min(K[mspecol  ==  minpmse])
    eta.opt <- max(eta[msperow  ==  minpmse])
    cat(paste("\nOptimal parameters: eta = ", eta.opt, ", ",
            sep = ""))
    cat(paste("K = ", K.opt, "\n", sep = ""))
    if (plot.it) {
        heatmap.spls(t(mspemat), xlab = "K", ylab = "eta", main = "CV MSPE Plot",
                     coln = 16, as = "n")
    }
    rownames(mspemat) <- paste("eta = ", eta)
    colnames(mspemat) <- paste("K = ", K)
    cv <- list(mspemat = mspemat, eta.opt = eta.opt, K.opt = K.opt)
    invisible(cv)
}

> predict.spls <- function (object, newx, type = c("fit", "coefficient"), ...)
```

```
{
    type    <- match.arg(type)
    betahat <- object$betahat
    x       <- object$x
    A       <- object$A
    cols    <- colnames(x)
    p       <- ncol(x)
    if (type == "fit") {
        if (missing(newx)) {
            pred <- x %*% betahat + matrix(1, nrow(x), 1) %*%
                object$mu
        }else {
            newx <- newx[, cols, drop = FALSE]
            if (ncol(newx) != p & ncol(newx) != length(A)) {
                stop("The dimension of test dataset is inappropprite!")
            }
            if (ncol(newx) == p) {
                newx <- newx[, A, drop = FALSE]
            }
            newx <- scale(newx, object$meanx[A], object$normx[A])
            pred <- newx %*% betahat[A, , drop = FALSE] + matrix(1,
                nrow(newx), 1) %*% object$mu
        }
    }
    if (type == "coefficient") {
        pred <- betahat
    }
    invisible(pred)
}

> trace.mspe <- function(fit.cv, header = "", col = our.colscheme,
                         ylim = "gen", xlim = "gen",
                         lty = rep(1, dim(fit.cv$mspemat)[2])){
    colMap <- colors()[rep(c(272, 177, 297, 201, 321, 225, 418, 355), 2)]
    X      <- fit.cv$mspemat
    eta    <- as.numeric(substring(rownames(X), first = 6))
    dimX   <- dim(X)

    if(ylim[1] == "gen"){ylim   <-  c(min(X), max(X))}
    if(xlim[1] == "gen"){xlim   <-  c(min(eta), max(eta))}

    plot(eta, X[, 1], ylim = ylim, xlim = xlim,
         xlab = expression(eta),
         ylab = "CV MSPE", type = "n", main = header)
```

```
    for(i in 1:dimX[2]){
        lines(eta, X[, i], lty = lty[i], col = col[i], lwd = 1.5)
    }

    legend("bottomright", bty = "n",
           title  = "k",
           legend = as.character(1:dimX[2]),
           lty    = lty,
           col    = col)
}
```

16.4 Functions used in the Simple Analysis

Function to make simple sensitivity predictions

```
> my.predict <- function(x,y){
    x %*% y
  }
```

16.5 Annotation and Plotting Tools

```
> lookUpPubmed <- function(spls.fit, n.coef = 3,
                           query.1 = "std"){
      Coef <- coef(spls.fit)
      Coef <- Coef[Coef != 0, ]
      Coef <- round(Coef[order(Coef)], n.coef)
      resTable  <- data.frame(geneSymbol = as.character(
                              unlist(lookUp(names(Coef),
                                         "hgu133plus2","SYMBOL"))))

      rownames(resTable) <- gsub("_","\\\\textunderscore ", names(Coef))

      anTable <- aafTableAnn(names(Coef), "hgu133a2.db", aaf.handler())
      band    <- rep("", length(resTable[, 1]))

      for(i in 1:length(resTable[, 1])){
          band[i] <- paste(anTable[[8]][[i]]@band, collapse = ", ")
      }

          resTable$mean   <- round(spls.fit$meanx[spls.fit$A], n.coef)
          resTable$sd     <- round(spls.fit$normx[spls.fit$A], n.coef)
          resTable$Locus  <- band
          resTable$weight <- Coef
```

```
        resTable$pmid   <- 0

    for(i in 1:length(resTable[, 1])){
        if(query.1 == "std"){
            query    <-
                paste(resTable[i,]$geneSymbol,
                      ' AND chemotherapy AND (resistance OR sensitivity)',
                      sep = "")

        }else{
            query <- query.1
        }

        query    <- gsub('\\s+' ,'+', query)

        url      <-
            "http://eutils.ncbi.nlm.nih.gov/entrez/eutils/esearch.fcgi?retmax=50000"

        url      <- paste(url, "&db=pubmed&term=", query, sep = "")

        datafile <- tempfile(pattern = "pub")
        try(download.file(url, destfile = datafile, method = "internal",
                          mode = "wb", quiet = TRUE), silent = TRUE)

        xml <- xmlTreeParse(datafile, asTree = TRUE)
        nid <- xmlValue(xmlElementsByTagName(xmlRoot(xml), "Count")[[1]])
        #lid <- xmlElementsByTagName(xmlRoot(xml), "IdList", recursive = TRUE)[[1]]

        #pmidlist <- unlist(lapply(xmlElementsByTagName(lid, "Id"), xmlValue))

        resTable[i, ]$pmid <- as.numeric(nid)#length(pmidlist)
    }
    colnames(resTable) <- c("Gene Symbol", "Mean", "SD", "Location", "Weight", "PMID" ,
    return(resTable)
}

> PlotKM.sda <- function(index,surv.object,
                         col    = c("black", "darkgrey", "red"),
                         legend = c("Sensitive", "Intermediate", "Resistant"),
                         col.leg = col,
                         lty    = c(1, 1, 1),
                         main   = "",
                         ylab   = "",
                         xlab   = "",
```

```
                   xmax   = 110,
                   ...) {

levels <- levels(index)

plot(survfit(surv.object ~ index),
     col  = col,
     lwd  = 2,
     xlab = xlab,
     ylab = ylab,
     xmax = xmax,
     main = main)


logRankTest <- survdiff(surv.object ~ index)

nchar <- max(nchar(legend)) - nchar(legend) + 1
spaces <- vector()

for(i in 1:length(legend)){
    spaces[i] <- paste(rep(" ", nchar[i]), sep = "", collapse = "")
}
xx <- as.matrix(table(predictDLDAArkansas$class))
xx <- xx[legend,]
nchar <- max(nchar(xx)) - nchar(xx) + 1
spaces2 <- vector()

for(i in 1:length(legend)){
    spaces2[i] <- paste(rep(" ", nchar[i]), sep = "", collapse = "")
}


old.par <- par(no.readonly = TRUE)
on.exit(par(old.par))

par(family = 'mono')

legend("bottomleft",
       legend  = paste(legend, ",", spaces, "n = ", spaces2,
       xx, sep = ""),
       bty     = "n",
       col     = col.leg,
       lty     = lty,
       lwd     = 2)
```

```
    legend("bottomright",
           bty = "n",
           legend = paste("P-value = ",
           as.character(signif(1-pchisq(logRankTest$chisq, 1), 1)), sep = ""))

    return(signif(1-pchisq(logRankTest$chisq, 1), 1))

  }

> CalcHR <- function(index,surv.data,surv.ind){

    threshold <- cut(index,
                     c(min(index) - 1,
                       quantile(index, cut.points),
                       max(index) + 1 ))

    uthres    <- levels(threshold)[c(1,3)]

    thres     <- as.character(threshold[threshold == uthres[1] |
                                        threshold == uthres[2]])

    new.index <- index[threshold == uthres[1] |
                       threshold == uthres[2]]

    new.surv     <- surv.data[threshold == uthres[1] |
                              threshold == uthres[2]]
    new.ind      <- surv.ind[threshold == uthres[1] |
                             threshold == uthres[2]]

    new.surv.object <- Surv(as.numeric(new.surv),new.ind)

    fit <- coxph(new.surv.object ~ as.factor(thres))

    coef.m <- -1*fit$coef
    std.m  <- sqrt(fit$var)
    return(
           paste("HR = ",signif(exp(coef.m),2), " (",
                 signif(exp(coef.m)-1.96*std.m,3),",",
                 signif(exp(coef.m)+1.96*std.m,3),")",
                 sep="")
           )
  }
>
```

```
> PlotKM <- function(index,surv.object,
                    cut.points   = c(1/3, 2/3),
                    our.colscheme = c("black", "darkgrey", "red"),
                    legend     = c("Sensitive", "Intermediate", "Resistant"),
                    lty      = c(1, 1, 1),
                    main      = "",
                    ylab = "",
                    xlab = "",
                    ...) {

      # Function to perform KM plot

      threshold <- cut(index,
                      c(min(index) - 1,
                        quantile(index, cut.points),
                        max(index) + 1 ))

      levels <- levels(threshold)

      plot(survfit(surv.object ~ threshold),
           col  = our.colscheme,
           lwd  = 2,
           xlab = xlab,
           ylab = ylab,
           main = main, ...)

      logRankTest <- survdiff(surv.object ~ threshold)

      nchar <- max(nchar(legend)) - nchar(legend) + 1
      spaces <- vector()

      for(i in 1:length(legend)){
          spaces[i] <- paste(rep(" ", nchar[i]), sep = "", collapse = "")
      }

      nchar <- max(nchar(summary(threshold))) - nchar(summary(threshold)) + 1
      spaces2 <- vector()

      for(i in 1:length(legend)){
          spaces2[i] <- paste(rep(" ", nchar[i]), sep = "", collapse = "")
      }


      old.par <- par(no.readonly = TRUE)
```

```
    on.exit(par(old.par))

    par(family = 'mono')
    legend("bottomleft",
           legend = paste(legend, ",", spaces, "n = ", spaces2,
           summary(threshold), sep = ""),
           bty   = "n",
           col   = our.colscheme,
           lty   = lty,
           lwd   = 2)

    legend("bottomright",
           bty = "n",
           legend = paste("P-value = ",
           as.character(signif(1-pchisq(logRankTest$chisq, 1), 1)), sep = ""))

    return(signif(1-pchisq(logRankTest$chisq, 1), 1))

}
```
